# Supplementary material for: Mixed-methods study on pharmacies as contraception providers to Kenyan young people: who uses them and why?
Source: BMJ Open. 2020 Jul 8;10(7):e034769. doi: 10.1136/bmjopen-2019-034769 (PMC7348460; doi:10.1136/bmjopen-2019-034769)
Supplement: Supplementary data [file bmjopen-2019-034769supp002.pdf]

S2. In-Depth Interview guide for young contraception purchasers

Warm-up

- Tell me about what life is like for young people (people your age) in this community.
- What are some of the challenges that young people face?

As you told us earlier, you recently purchased family planning from a chemist shop nearby. I want to ask you about this experience

- Tell me about what your experience was like when purchasing FP from the chemist – how did it go, from beginning to end? *[looking for information on environment, interaction with chemists, how they were treated]*
  - How did you feel at each step?
  - What was the most difficult part of the experience?
  - What was the easiest part of the experience?
- Describe your interaction with the chemist attendant *[probe on: how were you treated? Did they give you advice]*
  - How did he/she react to your request
- Tell me about the information you were given by the chemist *[probe on: counselling, life advice, side effects, referrals other FP]*
- Tell me about what else was going on in the chemist shop while you were purchasing FP.
- How did you feel after you left the shop?
- Given the experience you've just described to me, how did that compare with what you *thought* would happen when you first walked in the chemist shop? *

Thinking about the time that you purchased family planning at the chemist, help me understand how you made that decision:

- What situation made you decide that you needed family planning? *[Probe on whether others were involved in this decision]*
- How did you decide what kind of family planning you wanted?
- Why did you decide to go to a chemist for family planning instead of other places?
  - Why did you select that particular chemist?
- How did this experience compare with other times you have bought family planning?

As a young person who has purchased FP from a chemist, I am interested to hear your ideas for how chemist shops could be improved for young people:

- Were there any parts of your experience that you liked?
- Based on your experience, was there anything you would've <u>liked</u> to happen that didn't?
  - *Probe (if necessary):* Based on these, is there any part of the experience that you want changed?
- If you worked at the chemist, what would you do to make young clients buying family planning feel comfortable?
- If you worked at the chemist, how could you improve the shop to be more friendly to young people needing family planning?

- What else do young people need to feel comfortable getting FP from chemists?
