# Supplementary material for: Mixed-methods study on pharmacies as contraception providers to Kenyan young people: who uses them and why?
Source: BMJ Open. 2020 Jul 8;10(7):e034769. doi: 10.1136/bmjopen-2019-034769 (PMC7348460; doi:10.1136/bmjopen-2019-034769)
Supplement: Supplementary data [file bmjopen-2019-034769supp003.pdf]

S3. Key Informant Interview guide (for a person working in a pharmacy)

Group 1 (Background – Personal)

- Tell me about yourself and how you came to work in the chemist?
  - Probe if they are from the area
  - What is their current title?
- Tell me about the roles and responsibilities of your job - describe a typical day of work
- What are the things that you enjoy about your job?
- What are the things you do not enjoy about your job?

Group 2 (Background – Shop)

- Tell me about who else works at this chemist
  - Probe: what are their roles and how are they different from yours?
- Describe how the chemist shop is organized?
- When are you busiest?
  - Probe: opening hours

Group 3 (Family planning)

- Tell me about the family planning in this chemist shop
  - Probe: what kinds are available, most popular, price
- Tell me about the kinds of people from the community who buy these family planning
  - Probe: Describe them, what they are looking for
- Why are chemist shops like yours important in providing family planning to the community?
  - Probe: How is this job different from health facilities that also have family planning?
- If a young person comes in asking for family planning, what are some of things you look at that help you decide what to recommend?
- What are the rules for dispensing family planning?
  - Probe: are there any exceptions to these rules?
- Describe the kinds training (either from your boss or from previous training) you received about family planning?

Group 4 (Feelings about selling family planning to young people)

- Think about the last time that young person (18-24) came to this chemist for some kind of family planning. Can you describe the interaction, from beginning to end?
  - Probe: what happens, what would they say, what would you say, what do you give them?
- How do young customers feel coming to ask for family planning (Probe: what do they say)
- What could chemist shops like this one do to improve the comfort of young people in the community who need family planning?
- When a young customer comes in asking for family planning, how do you feel?

    - Are things you would like to tell them?
- If you had the power, what would you do to improve the confidence of chemists to provide family planning to young people?
