# Supplementary material for: Mixed-methods study on pharmacies as contraception providers to Kenyan young people: who uses them and why?
Source: BMJ Open. 2020 Jul 8;10(7):e034769. doi: 10.1136/bmjopen-2019-034769 (PMC7348460; doi:10.1136/bmjopen-2019-034769)
Supplement: Supplementary data [file bmjopen-2019-034769supp004.pdf]

S4. Tool 2 – Survey Instrument (Excerpt)

SOCIO-DEMOGRAPHIC INFORMATION. First we're going to talk about who you are.

1. What is your sex? Mark ONLY ONE.

    0 Male
    1 Female

2. What is your birthdate?

    Day |__|__| Month |__|__| Year |__|__|__|__|

3. What is the highest level of school you attended? Mark ONLY ONE.

    0 I've never gone to school
    1 Primary school
    2 Secondary school
    3 Post-secondary education **– GO TO 5**

4. What is the highest grade you completed at that level?

    |__|__| grade/form/level **– GO TO 6**

5. What type of post-secondary education did you attend/are you attending? Mark ONLY ONE.

    1 Technical post-secondary education
    2 University education

6. Who do you currently live with? Mark ALL possible options.

    0 I live alone
    1 Father/stepfather
    2 Mother/stepmother
    3 Siblings
    4 Grandparents
    5 Other relatives

6 Husband or wife **– *NOTE: Be sure to ask whether husband/wife or cohabiting partner.***
7 Cohabiting partner
8 In-laws
9 Children
10 Friends

7. What is your current relationship status?
   0 Single
   1 Friends with benefits
   2 Dating
   3 Cohabiting
   4 Engaged
   5 Married
   6 Other (specify)

8. How many children do you have?

   0 I have no children **– GO TO 10**

   |__|__| child/children

9. How old were you when you had your first child?

   |__|__| years old

…
[SURVEY CONTINUES]

ARMADILLO-RELATED BEHAVIORS. Now we're going to talk about sexual activity in order to gain a better understanding of some important life issues. Let me assure you again that your answers are completely confidential and will not be told to anyone.

29. How old were you when you had sexual intercourse for the very first time?

   |__|__| years old

   0 I have never had sexual intercourse **– GO TO 46**

30. Have you ever used any method to prevent pregnancy? By use, I mean that either you, yourself, have used the method or that a partner of yours used the method when having sex with you.

YES 1
NO...0
DON'T KNOW..8
REFUSED 9

31. When was the last time you had sex?

|__|__| days ago

|__|__| weeks ago

|__|__| months ago

|__|__| years ago

32. The last time you had sex, what was your relationship to this person with whom you had sexual intercourse?

1 Boyfriend not living with respondent
2 Girlfriend not living with respondent
3 Male cohabiting partner
4 Female cohabiting partner
5 Husband
6 Wife
7 Male casual acquaintance
8 Female casual acquaintance
9 Male sex worker
10 Female sex worker
11 Female client (respondent is male sex worker)
12 Male client (respondent is female sex worker)
13 Male relative
14 Female relative

33. The last time you had sex, did you or your partner use a contraceptive method?

0 No **– GO TO 45**
1 Yes

34. The last time you had sex, which contraceptive method did you or your partner use?

1 Male condom
2 Female condom
3 Birth control pill
4 Injectable
5 Implant
6 Intrauterine device (IUD)
7 Emergency contraception (the morning after pill)
8 Female sterilization
9 Male sterilization (vasectomy)
a. Withdrawal **– GO TO 44**
10 Rhythm method **– GO TO 44**

35. The last time you had sex, where did you or your partner obtain the contraceptive method you used?

1 A pharmacy or chemist
2 County Hospital
3 Health centres
4 A NGO
5 A private doctor or clinic
6 A shop/market
7 A community-based distributor
8 A peer educator
9 A traditional healer **– GO TO 44**
10 A friend or relative **– GO TO 44**
11 A partner **– GO TO 44**
12 Other **– GO TO 44**

36. When you obtained your [MOST RECENT CONTRACEPTIVE METHOD], were you told by the provider about side effects of problems you might have with a method to delay or avoid getting pregnant?
1 Yes
2 No
37. Were you told what to do if you experienced side effects or problems?
1 Yes
2 No

38. At that time, were you told by the family planning provider about methods of family planning other than [MOST RECENT CONTRACEPTIVE METHOD] that you could use?
1 Yes
2 No

39. During that visit did you obtain the method you wanted to delay or avoid getting pregnant?
1 Yes **– GO TO 42**

2 No

40. Why didn't you obtain the method you wanted?
    1 Method out of stock that day
    2 Method not available at all
    3 Provider not trained to provide the method
    4 Provider recommended a different method
    5 Not eligible for method
    6 Decided not to adopt a method
    7 Too costly
    8 Other

41. During that visit who made the final decision about what method you got?
    1 You alone
    2 Provider
    3 Partner
    4 You and provider
    5 You and partner
    6 Other

42. Would you return to this provider?
    1 Yes
    2 No

43. Would you refer your relative or friend to this provider/facility?
    1 Yes
    2 No

44. How important were each of the following characteristics to you in deciding which birth control method to use? *(read item, asking)* Would you say: not at all important, slightly important, quite important or extremely important to you in choosing a method?

	Not at all important	Slightly important	Quite important	Extremely important
A. It is very effective at preventing pregnancy				
B. It has a low cost.				

C. It is easy to use.				
D. It doesn't contain hormones.				
E. It is acceptable to my partner<br>F. It doesn't interrupt sex.				
G. It is effective at preventing HIV or STIs.				
