# Supplementary material for: Mixed-methods study on pharmacies as contraception providers to Kenyan young people: who uses them and why?
Source: BMJ Open. 2020 Jul 8;10(7):e034769. doi: 10.1136/bmjopen-2019-034769 (PMC7348460; doi:10.1136/bmjopen-2019-034769)
Supplement: Supplementary data [file bmjopen-2019-034769supp005.pdf]

Supplementary Table 1 Selected characteristics of young people purchasing contraception at a pharmacy, shop, or any other source

	Pharmacy (N=153)	Shop (N=29)	Any other source (N=61)
Age			
18-19	27 (18%)	6 (21%)	10 (16%)
20+	126 (82%)	23 (79%)	51 (84%)
Sex			
Male	80 (52%)	24 (83%)	28 (46%)
Female	73 (48%)	5 (17%)	33 (54%)
Education			
Primary or below	60 (39%)	7 (24%)	29 (48%)
Secondary or above	93 (61%)	22 (76%)	32 (52%)
Relationship status			
Single	46 (30%)	10 (34%)	25 (41%)
Dating	86 (56%)	18 (62%)	11 (18%)
Cohabiting/Married	21 (14%)	1 (3%)	25 (41%)
Children			
No	139 (91%)	28 (97%)	37 (61%)
Yes	14 (9%)	1 (3%)	24 (39%)
Living situation			
Lives alone	30 (20%)	3 (10%)	6 (10%)
Lives with family (dependent)	113 (74%)	25 (86%)	32 (53%)
Lives with child or partner	10 (7%)	1 (3%)	23 (38%)
Contraception purchased			
Condom	120 (78%)	28 (97%)	33 (54%)
ECP	24 (16%)	1 (3%)	5 (8%)
Pills/Injections	9 (6%)	0 (0%)	23 (38%)

Gonsalves L, *et al. BMJ Open* 2023; 10:e034769. doi: 10.1136/bmjopen-2019-034769
