# Supplementary material for: Mixed-methods study on pharmacies as contraception providers to Kenyan young people: who uses them and why?
Source: BMJ Open. 2020 Jul 8;10(7):e034769. doi: 10.1136/bmjopen-2019-034769 (PMC7348460; doi:10.1136/bmjopen-2019-034769)
Supplement: Author's manuscript [file bmjopen-2019-034769.draft_revisions.pdf]

BMJ Open

BMJ Open is committed to open peer review. As part of this commitment we make the peer review history of every article we publish publicly available.

When an article is published we post the peer reviewers' comments and the authors' responses online. We also post the versions of the paper that were used during peer review. These are the versions that the peer review comments apply to.

The versions of the paper that follow are the versions that were submitted during the peer review process. They are not the versions of record or the final published versions. They should not be cited or distributed as the published version of this manuscript.

BMJ Open is an open access journal and the full, final, typeset and author-corrected version of record of the manuscript is available on our site with no access controls, subscription charges or pay-per-view fees (http://bmjopen.bmj.com).

If you have any questions on BMJ Open's open peer review process please email info.bmjopen@bmj.com

BMJ Open

Mixed-methods study on pharmacies as contraception providers to Kenyan young people: who uses them and why?

Journal:	*BMJ Open*
Manuscript ID	bmjopen-2019-034769
Article Type:	Original research
Date Submitted by the Author:	04-Oct-2019

Complete List of Authors:	Gonsalves, Lianne; World Health Organization, Department of Reproductive Health and Research including UNDP/UNFPA/UNICEF/WHO/World Bank Special Programme of Research, Development and Research Training in Human Reproduction (HRP); Swiss Tropical and Public Health Institute<br>Wyss, Kaspar; Swiss Tropical and Public Health Institute; University of Basel<br>Cresswell , Jenny; World Health Organization, Department of Reproductive Health and Research including UNDP/UNFPA/UNICEF/WHO/World Bank Special Programme of Research, Development and Research Training in Human Reproduction (HRP)<br>Waithaka, Michael; International Centre for Reproductive Health Kenya<br>Gichangi, Peter; International Centre for Reproductive Health Kenya; University of Nairobi, Department of Human Anatomy<br>Martin Hilber, Adriane; Swiss Tropical and Public Health Institute; University of Basel

Keywords:	REPRODUCTIVE MEDICINE, Community child health < PAEDIATRICS, PUBLIC HEALTH, QUALITATIVE RESEARCH

SCHOLARONE™
Manuscripts

For peer review only - http://bmjopen.bmj.com/site/about/guidelines.xhtml

BMJ

I, the Submitting Author has the right to grant and does grant on behalf of all authors of the Work (as defined in the below author licence), an exclusive licence and/or a non-exclusive licence for contributions from authors who are: i) UK Crown employees; ii) where BMJ has agreed a CC-BY licence shall apply, and/or iii) in accordance with the terms applicable for US Federal Government officers or employees acting as part of their official duties; on a worldwide, perpetual, irrevocable, royalty-free basis to BMJ Publishing Group Ltd ("BMJ") its licensees and where the relevant Journal is co-owned by BMJ to the co-owners of the Journal, to publish the Work in this journal and any other BMJ products and to exploit all rights, as set out in our licence.

The Submitting Author accepts and understands that any supply made under these terms is made by BMJ to the Submitting Author unless you are acting as an employee on behalf of your employer or a postgraduate student of an affiliated institution which is paying any applicable article publishing charge ("APC") for Open Access articles. Where the Submitting Author wishes to make the Work available on an Open Access basis (and intends to pay the relevant APC), the terms of reuse of such Open Access shall be governed by a Creative Commons licence – details of these licences and which Creative Commons licence will apply to this Work are set out in our licence referred to above.

Other than as permitted in any relevant BMJ Author's Self Archiving Policies, I confirm this Work has not been accepted for publication elsewhere, is not being considered for publication elsewhere and does not duplicate material already published. I confirm all authors consent to publication of this Work and authorise the granting of this licence.

For peer review only

Mixed-methods study on pharmacies as contraception providers to Kenyan young people: who uses them and why?

Lianne Gonsalves[123], Kaspar Wyss[23], Jenny A Cresswell[1], Michael Waithaka[4], Peter Gichangi[456], Adriane Martin Hilber[23]

[1]Department of Reproductive Health and Research including UNDP/UNFPA/UNICEF/WHO/World Bank Special Programme of Research, Development and Research Training in Human Reproduction (HRP), World Health Organization, Geneva, Switzerland
[2]Swiss Tropical and Public Health Institute (Swiss TPH), Basel, Switzerland
[3]University of Basel, Basel, Switzerland
[4]International Centre for Reproductive Health Kenya, Mombasa, Kenya
[5]Department of Human Anatomy, University of Nairobi, Nairobi, Kenya
[6]Ghent University, Ghent, Belgium

Corresponding author information:

Lianne Gonsalves
Address: World Health Organization, Avenue Appia 20, 1211 Geneva, Switzerland
Telephone: +41 22 791 29 70
Email address: gonsalvesl@who.int

For peer review only

Word count, excluding title page, abstract, references, figures and tables

3961

Abstract: 300 words

Background

Public sector contraceptive services often struggle to meet the needs of young people around the world. Instead, private pharmacies have been demonstrated to be a relied-upon source of modern contraception for young people.

Objectives

This study sought to answer two questions: 1) what are the characteristics of young Kenyans aged 18-24 who use contraception obtained at pharmacies, and 2) why are pharmacies appealing sources of contraception?

Design and Setting

This was a mixed-methods study in one peri-urban part of Kwale County, Kenya. Methods included: cross-sectional survey (N=740); six focus group discussions; 18 in-depth interviews; and 25 key informant interviews. Quantitative data analysis identified factors pushing young people to pharmacies for contraception versus other sources. Qualitative data analysis identified reasons pharmacies were perceived to be appealing to young clients.

Participants

Participants were: 1) young people aged 18-24 from the study area, including a a subset who had recently purchased contraception from a pharmacy; or 2) pharmacy personnel and pharmacy stakeholders.

Results

Among surveyed participants, 59% had used contraception purchased from a pharmacy at last sex. In multivariable analysis, participants who had used a condom or emergency contraception as well as those living alone were significantly more likely to get contraception from pharmacies. Pharmacies were valued for their: convenience; privacy; non-judgmental and personable staff; service speed; and predictable, affordable prices.

Conclusions

Our findings indicate a higher percentage of young people than previously reported use pharmacies for contraception. Our inclusion of emergency contraception users and young men partially explain this. Additionally, pharmacies were perceived to be everything that health facilities were not: fast, private and non-limiting. Policymakers should recognize the role of pharmacies as contraception providers and look for opportunities to link pharmacies to the public health system. This would create a network of accessible and appealing contraception services for young people.

Article Summary

- The phrasing of survey questions affected our ability to distinguish differences between young men versus young women who obtain contraception.
- One participant group (young people who had recently purchased contraception from a pharmacy) was recruited from five purposively selected pharmacies: this may limit the generalizability of the findings.
- This study is strengthened by its mixed methods design and inclusion of both pharmacy personnel and young people to triangulate research findings on a sensitive subject.

For peer review only

INTRODUCTION

Young people need access to contraception: however, around the world, and in low- and middle-income countries in particular, public sector contraceptive services are not meeting this need. Indeed, young people are often reluctant to access contraception at public health facilities where they may encounter a lack of privacy, biased providers, and limited contraceptive options, in addition to broader financial, legal, social, and cultural barriers. [1, 2]

Data from 61 low- and middle-income countries estimated that 33 million young women aged 15-24 had an unmet need for family planning. [3] In Kenya, where this study took place, the 2014 Demographic and Health Survey found that among currently married women, 23% of 15-19 year-olds and 19% of 20-24 year-olds have an unmet need for family planning. [4] Among sexually active unmarried women, 50% of 15-19 year-olds and 31% of 20-24 year-olds were not using any contraceptive method. [4]

Other parts of the health system may be able to step in to help fill this gap. In Kenya and in the region, private pharmacies have been demonstrated to be a relied-upon source of modern contraception for young people [5-8]. Additional research has indicated that when contraception is introduced in pharmacies, access improved for young people.[9, 10] An analysis of 33 sub-Saharan African countries found that commercial drug sellers, including pharmacies, were the source of the most recent contraceptive method for nearly one in five young people between 15-24 years of age. [9] When also factoring in other informal and non-medical providers, including shops, these sources together serviced nearly half of women age 15-19. [9]

Kenya's National Family Planning Guidelines allow for the provision of several barrier methods and short-acting forms of contraception to be dispensed in private retail pharmacies [11](colloquially referred to as 'chemists'). These include male and female condoms, emergency contraception (ECP), oral contraceptive pills, and injectable contraception (which can be dispensed but not administered). However, despite their demonstrable popularity among young people, there is little data on the individual-level circumstances or characteristics of young people that would drive them to pharmacies for contraception. Therefore, this mixed methods study sought to answer two questions: 1) what are the characteristics of young people who use contraception obtained at pharmacies, and 2) why are pharmacies appealing sources of contraception to young people?

METHODS

This analysis was part of a broader, mixed-methods study describing how young people (aged 18-24) in Kwale County obtain contraception from pharmacies. Kwale County is one of six counties in Kenya's former Coast region: the study itself took place in the peri-urban area of Kwale Town and Ukunda, as well as the stretch of highway connecting the two. Young people between the ages of 15-24 were projected to make up 19% of the County's population by 2018.[12] In 2014, contraception prevalence in the county was 38%, lower than the national average of 53%. [13]

This study was partly-nested in the ARMADILLO randomized controlled trial (RCT)[14], assessing the effect of an unrelated digital health intervention on SRH-related outcomes for young people aged 18-24. Data collection took place between October 2017 and March 2018. We used several methods (captured in Table 1) to triangulate from the perspectives of pharmacy personnel and young people themselves, an understanding of what kinds of young people purchase contraception from pharmacies and why pharmacies are considered appealing sources of contraception.

Table 1 Study Methods

For peer review only - http://bmjopen.bmj.com/site/about/guidelines.xhtml

Method	N	Elgibility criteria	Relevant topics addressed
**Cross-sectional survey***	**740**	• Age 18-24<br>• Literate<br>• Have their own mobile phone (with them at time of recruitment) and report regular use<br>• Report current use of text messaging	• Contraception used at last sex and source<br>• Demographic and behavioral characteristics
**Focus group discussions***	**6 (58 participants)**	• Age 18-24<br>• Community members	• Sources of contraception for young people<br>• Characteristics of young people who use each source
**In-depth interviews**	**18**	• Age 18-24<br>• Recently purchased contraception at pharmacy	• Reasons for having purchased contraception from pharmacy<br>• What was valued (and not valued) about experience
**Key-informant interviews**	**19 (pharmacy personnel)<br>6 (stakeholders)**	• Age 18+<br>• Pharmacy personnel (any role) OR<br>• Pharmacy-related stakeholder (Ministry of Health; regulatory agency; professional association; non-governmental organization)	• Characteristics of young people who purchase contraception<br>• What clients appreciate about experience

***** Methods which were nested in the broader ARMADILLO Study, a digital health intervention RCT. Inclusion/exclusion criteria for these nested methods were determined by ARMADILLO's objectives.

To capture the perspectives of young people, a cross-sectional survey of 740 young people age 18-24 captured demographic information and contraceptive use patterns, including source of last contraception (these questions were one section of a broader survey conducted as part of the baseline assessment for the ARMADILLO trial). The sample size was calculated based on the ARMADILLO trial's primary outcome – the full protocol for the trial has been previously published[14], along with details of participants recruited.[15] In October 2017, data collectors enumerated all households with young people in the study area using a map provided by the Kenya National Bureau of Statistics. In February 2018, a random selection of households and random selection of one youth per household was generated for the purposes of participant recruitment.

Additionally, six Focus Group Discussions were conducted with young people age 18-24, purposively recruited from the community by data collectors. Finally, we conducted in-depth interviews with 18 young people aged 18-24 who had recently purchased contraception from pharmacies. We identified these young participants in one of two ways. First, we stationed a young data collector outside of well-trafficked pharmacies over three evenings, who recruited young people purchasing contraception. Second, several pharmacists in the study area were provided with leaflets with study information and requested to provide these to young contraception purchasers at the end of a transaction.

For peer review only - http://bmjopen.bmj.com/site/about/guidelines.xhtml

To capture the perspectives of pharmacy personnel, data collectors mapped all the private, retail pharmacies in the study area using a digital form with an embedded geolocator. A random subset of pharmacies was generated, and data collectors visited these to conduct key informant interviews with pharmacy personnel. Data collectors were instructed to interview the first person behind the counter they met, regardless of rank or level of training – 19 interviews in total were conducted. An additional six key-informant interviews were conducted with stakeholders from the Pharmacy and Poisons Board, Ministry of Health, professional associations, and non-governmental organizations: these were conducted in Ukunda, Mombasa, or Nairobi.

Data collection

We obtained informed consent from all participants prior to participation. All data was collected in English, Swahili, or a mix of the two, depending on participants' preference. Quantitative surveys were completed using webforms on a tablet. Data collectors entered responses save for the questions related to participants' sexual and contraceptive use history; here, to reduce potential discomfort, participants entered their own responses. Interviews and FGDs used semi-structured guides. Qualitative data collection ceased upon reaching saturation. All qualitative methods used audio-recording (with participant permission). All study activities were conducted in a private location. Data collectors, speaking both English and Swahili, were recruited from the study area and specifically trained for this study.

This study received ethics approval from the Ethikkommission Nordwest- und Zentralschweiz (EKNZ) (Req-2017-00389) in Basel, Switzerland, as well as the University of Nairobi/Kenyatta National Hospital in Nairobi, Kenya (P274/05/2017). The ARMADILLO RCT also received ethics approval from the World Health Organization (Protocol WHO A65892), and is registered with the ISRCTN Registry (ISRCTN85156148).

Patient and public involvement

• How was the development of the research question and outcome measures informed by patients' priorities, experience, and preferences?

The qualitative outcomes of this study directly reflect young people's experience and preference (we present WHY young people find pharmacies appealing).

• How did you involve patients in the design of this study?

Young people were directly involved in parts of the study's design. We relied on their insight and lived experience to determine how young people would feel most comfortable being recruited. Based on this, we jointly designed our recruitment and consenting procedures.

• Were patients involved in the recruitment to and conduct of the study?

Our survey data collection team consisted of young people recruited from the study area (Kwale County). Qualitative method data collectors were also young people recruited from both Kwale and Mombasa Counties.

• How will the results be disseminated to study participants?

No specific dissemination to participants was budgeted for. A dissemination meeting involving local, county, and national stakeholders (including some adult study participants) took place in June, 2019. At

For peer review only - http://bmjopen.bmj.com/site/about/guidelines.xhtml

For peer review only

the meeting, several young data collectors were invited to attend and they provided commentary on the findings.

Analysis

Quantitative data was analyzed in Stata Version 14. The subject of the analyses (as described in Figure 1) were survey participants who reported using one of four contraception commodities available in pharmacies (either male or female condom, ECP, daily contraceptive pills, or injectable contraception) at last sex *and* who reported their source. Excluded were those participants who had not used contraception at last sex, who had not used a contraceptive commodity (withdrawal method, calendar days), who could not remember where they had obtained their method and/or who had obtained it from a partner or friend. We developed a dichotomous 'source of family planning' outcome, distinguishing between 'pharmacy' and 'any other source'. The latter included any public or private health facility, community-based distributors, non-governmental organizations, shops, schools, supermarkets. Following descriptive statistics, bivariate log binomial regressions assessed the association between the outcome and each behavioral/sociodemographic variable of interest. Any analysis showing a p<.2 moved the variable into a multivariable Poisson regression model with robust 95% CIs.

Figure 1 Flow Diagram of Study Participants

All qualitative data was transcribed verbatim and then translated (if necessary) into English. We adopted an iterative approach to data collection and analysis, allowing question guides to be modified based on emerging themes. Qualitative analysis was conducted in Atlas.ti Version 8 and relied on thematic analysis, with inductive and deductive (the latter based on the research objective) coding of a subset of transcripts to develop and refine a coding framework.

RESULTS

Survey sample characteristics

As seen in Table 2, of the 740 young people aged 18-24 who participated in the cross-sectional survey, 512 (69%) had ever had sex. Male condoms were the single most popular form of contraception purchased, used by 190 of the 274 (69%) participants who used contraception at last sex. Of the participants indicating that they used a modern contraceptive at last sex (N=263), 154 (59%) had obtained it from a private, retail pharmacy (hereafter, 'pharmacy'). Of the 512 participants, 259 (51%) indicated they used a contraceptive method that is available in pharmacies at last sex. A majority of these participants (59%) also obtained their contraception from a pharmacy, while an additional 11% obtained it from a shop. Those obtaining it from a public health facility (dispensary, health centre or hospital) comprised another 18% in total.

Table 2 Baseline characteristics

**All surveyed participants (N=740)**	
Ever had sex	512/740 (69%)
Used any contraception at last sex	274/512 (54%)
Used a modern contraceptive at last sex	263/512(51%)
Used pharmacy-available contraception*	259/512 (51%
Where contraception was obtained **(N=259)**	
Pharmacy	59%
Shop	11%

For peer review only - http://bmjopen.bmj.com/site/about/guidelines.xhtml

Public dispensary or health centre	10%
Hospital	8%
NGO, private doctor	4%
Community-based distributor, school, supermarket	2%
*Other person***	*3%*
*Other source (not specified)/Don't know ***	*3%*
**Included participants using pharmacy-available contraception (N=243)**	
Age	
18-19	18%
20-24	82%
Sex	
Male	54%
Female	46%
Education (highest level attended)	
Primary or below	40%
Secondary	47%
Post-secondary	14%
Relationship status	
Single	33%
Dating	47%
Married/Cohabiting	19%
Any children	
No	84%
Yes	16%
Living situation	
Lives alone	16%
Lives with family (dependent)	70%
Lives with child or partner	14%
Contraception used***	
Male condom	72%
Female condom	2%
ECP	12%
Daily contraceptive pills	3%
Injection	10%

*these included male or female condom, emergency contraception (ECP), daily contraceptive pills, and injectable contraception
**these were excluded from analysis
***Participants could enter one contraceptive method

Of the 243 participants who were included in bivariate and multivariable analyses, 54% were male, 61% had attended secondary school or higher, and 70% were dependents (living with parents, grandparents, or other older family members). Among participants included in the analysis, male condoms remained the most popular (72%), followed by ECP (12%), and injection (10%). Supplementary Table 1 presents selected characteristics of the 243 participants disaggregated by whether they obtained contraception at a pharmacy, shop, or any other source: most shop users were male and purchased condoms.

For peer review only - http://bmjopen.bmj.com/site/about/guidelines.xhtml

Characteristics of young people who use a pharmacy to access contraception

Bivariate analyses (Table 3) indicated there was no evidence of an association between either age, sex, or education and a young person's contraception being from a pharmacy. There was however an association between pharmacy-purchased contraception and a participant's relationship status, and whether they had children. The greatest predictors of whether a young person had visited a pharmacy, were the type of contraception they purchased and with whom they lived. Following multivariate analysis (Table 3), there remained strong evidence of an association between pharmacy purchase of contraception and a young person's living situation as well as the type of contraception they used. Young people living alone were almost twice as likely to have sourced contraception from a pharmacy as those living with a child or partner (Adjusted PR 1.96, 95% CI [1.07-3.59]). Young people using condoms were more likely to have visited a pharmacy as compared with pill/injection users (Adjusted PR 1.87, 95% CI [1.02- 3.43]). However, use of ECP remained the greatest predictor of a pharmacy purchase (Adjusted PR 2.27 as compared with pill/injection use 95% CI [1.21-4.27]).

Table 3 Bivariate and multivariable analysis to identify personal characteristics that may be associated with a young person obtaining contraception from a pharmacy (vs any other source)

	Purchased contraception from pharmacy	Unadjusted Prevalence Ratio (PR) [95% CI]	p-value*	Adjusted Prevalence Ratio (PR) [95% CI]	p-value
All	153/243 (63%)				
Age					
18-19	27/43 (63%)	Ref			
20-24	126/200 (63%)	1.00 [0.78-1.29]	0.979		
Sex					
Male	80/132 (61%)	Ref			
Female	73/111 (66%)	1.09 [0.90-1.32]	0.405		
Education					
Primary or below	60/96 (63%)	Ref			
Secondary or above	93/147 (63%)	1.01 [0.83-1.23]	0.904		
Relationship status					
Single	46/81 (57%)	1.27 [0.88-1.84]	0.0013	0.78 [0.54-1.14]	0.0284
Dating	86/115 (75%)	1.67 [1.20-2.34]		1.04 [0.74-1.48]	
Married/Cohabiting	21/47 (45%)	Ref		Ref	
Children					
No	139/204 (68%)	1.89 [1.24-2.92]	0.003	1.25 [0.80-1.97]	0.318
Yes	14/39 (36%)	Ref		Ref	
Living situation					
Lives alone	30/39 (77%)	2.62 [1.51-4.53]	0.0024	1.96 [1.07-3.59]	0.0119
Lives with family (dependent)	113/170 (66%)	2.26 [1.33-3.85]		1.53 [0.84-2.82]	
Lives with child or partner	10/34 (29%)	Ref		Ref	
Contraception used					
Condom (m/f)	120/181 (66%)	2.36 [1.34-4.14]	0.0014	1.87 [1.02- 3.43]	
ECP	24/30 (80%)	2.84 [1.59-5.09]		2.27 [1.21-4.27]	0.0224

For peer review only - http://bmjopen.bmj.com/site/about/guidelines.xhtml

Pills/Injection	9/32 (28%)	Ref		Ref	

*any variable with p-values <.2 in bivariate analysis were included in the multivariable analysis

Why are pharmacies appealing?

Participants indicated that it was a combination of the pharmacy *facilities,* the pharmacy *personnel* themselves, and the *services* provided by the pharmacy which together made these establishments the preferred source of contraception for many young people (Table 4).

Table 4 Reasons why pharmacies are appealing (selected excerpts from qualitative data)

**Facility appeal**	
Convenience (locations and hours)	"The chemist is near and whenever you want it [family planning] you can access it, anytime." Female pharmacy purchaser: injection<br><br>"The good thing with chemist is that they are many of them…when you missed a certain contraceptive at a certain chemist you can go to the next chemist because they are several of them, not like the hospital" – Female community member (FGD)<br><br>"Yes, majority of them [young people] don't live near health centres. Second, health centres are usually busy. And it's not every day they [can be] attended to: there are specific days they have clinics… [The client] won't be able to make it there…even if the treatment was free. But there is a chemist - [they] can go for similar services." – Pharmacist
Privacy	"At the chemist there are not many people. I may go to Diani dispensary [a local public health facility], and there is someone who knows me and I go for family planning. I saw it would be better to go the chemist because I know that will be my secret and the attendant." Female pharmacy purchaser: emergency contraception<br><br>"When you go to the facility, when you go to the FP room, everyone knows that you've gone to get FP. For young people [especially] because no one will want to see me - I'm 18, I'm 16 and I'm already using family planning. I'm not supposed to be sexually active. The kind of population that is in those FP areas, around those FP areas it's your mothers who are either breastfeeding, or they're pregnant and have gone for ANC." – Ministry of Health official, County level
**Personnel appeal**	
Interpersonal relationship	"the chemist is just within the neighborhood and I know the guy he is my friend outside job so it wasn't stressful for me in fact it was really fast and easy." – Male pharmacy purchaser: ECP and condoms<br><br>"The person in charge is my friend, I can go to him with my problems and he would assist me, he is not that far for me to reach him with my phone - he is my neighbor I could have a problem even at night and be able to reach out to him." -Male pharmacy purchaser: ECP
Seen as part of the community	"I chose it because it has been there for many years even before I was born till the time I finished school. The attendants are just normal. Many people get help from there so I saw it good to also go there." – Female pharmacy purchaser: ECP and injection

For peer review only

For peer review only - http://bmjopen.bmj.com/site/about/guidelines.xhtml

	"What I had said about the hospital, when you get there you will find the person who served you before is transferred but when you come to the chemist you will find the person that served you before." – Female community member (FGD)
Non-judgmental	"I thought at the chemist they will understand me and I would talk to them [better] than at the hospital where they will say I do not need to use those things or even talk to me harshly." –Male pharmacy purchaser: ECP and condoms<br><br>"At the chemist, that person wants - since it is a business – [to] just give, as compared to the hospital where when you get there you will find nurses who are arrogant or other doctors who will insult you." Male community member (FGD)
**Service appeal**	
Speed	You know at the dispensary it is a must you meet with the doctor for more explanation. And maybe there is a service you need to pay for, the expenses are many at the dispensary unlike the chemist where everything is fast, when you get there you get what you want and leave. – Youth female, has purchased ECP and condoms<br><br>"You get in a hospital, there are so many people queueing outside that are waiting to see a doctor. Here comes a young lady who is in a hurry. That particular person will find it more convenient to go to a chemist shop rather than going to a hospital." – Pharmacist
Cost	It is not easy for the government hospital. It is best, if you have money, you go to private hospitals. Now that is why you see if someone does not have money, or us the young people, we just go to the chemist because there is no cash to see a doctor for Ksh 600. At the chemist you just go direct and you are served. – Male pharmacy purchaser: ECP and condoms<br><br>Chemists are not expensive like hospitals. In hospital you can be told it is a government hospital but you end up being asked to give out a lot of money. In [the] chemist the money you get asked is for[paying for] P2 [an emergency contraceptive], yah but in hospital you will be told to do some test because we think it is this and this.– Female pharmacy purchaser: ECP<br><br>Free does not always mean free. Sometimes, something will be free, but by the time you get it, the process is a lot. Because for us, we don't just offer family planning, we do [mandatory] counselling. The person who is going to a chemist is someone who has made up his or her mind. But in the public facilities, you are counselled, you are explained to, you are told the different methods, then you are given a chance to make an informed choice. So, I think that…is a barrier somehow. – Ministry of Health Official, County level

Pharmacy facilities – that is, the environments themselves – were deemed appealing because of the convenience and privacy they offered young clients. Pharmacies were located where young people lived, worked, and spent time, making them easy points of family planning access. If one pharmacy lacked what a young person was looking for, it was an easy trip to the next one. 'Convenience' also extended to the days and hours pharmacies were open. This made them especially important on days where health facilities were known to be busy, or evening and weekend hours when young people might need contraception.

For peer review only - http://bmjopen.bmj.com/site/about/guidelines.xhtml

Additionally, the relative privacy offered by pharmacies was especially important to young clients. Participants perceived pharmacies, with interactions limited to a pharmacy attendant and a client, to be far more discreet than similar services offered at public health facilities. Public health facilities had public waiting areas where young people may see someone they knew. Additionally, services in the health facility might be categorized by service type (for example, contraceptive services separated from immunization services, etc). This left young clients feeling particularly exposed should they need to walk up to a labeled 'family planning' window or step forward if a public announcement about contraceptive services was made.

The individuals behind the counter, and how they interacted with young people, were additional reasons young people preferred to obtain contraception from pharmacies. Pharmacy personnel were perceived to be established, fellow community members. Young clients appreciated seeing the same familiar faces, with less of the personnel turnover associated with public health facilities. When personnel were a similar age to young clients (a very strong preference of all young participants), many reported being able to communicate openly with pharmacy personnel and being more comfortable interacting with them.

Pharmacy personnel were perceived to be non-judgmental compared with those working in health facilities. There was a perception that a trip to a facility would result in difficult questions, and a possible refusal to provide the desired contraceptive. Pharmacy personnel, by contrast, would treat young people well and would provide the desired contraceptive. Several participants speculated that the for-profit aspect of pharmacies could be a reason that they were treated better and not refused services.

Finally, pharmacy contraception services themselves were appreciated for being fast and cheap. Participants routinely referenced the queueing for services and long wait times driving young people away from health facilities and into pharmacies instead. Additionally, services were perceived to be cheaper than both private health facility services as well as public health facility services. Services at private health facilities were considered out of financial reach for most young people – making a chemist a more affordable option. At public health facilities, where contraception-related services are meant to be free, participants indicated that this was often not the case in practice. Expenses related to travel, or 'tests' (for example, a pregnancy test) ordered by health care providers prior to dispensing contraception made actual costs related to public services difficult to gauge. Finally, as one government official acknowledged, even when services were free, the time and processes required could deter young people who knew what they wanted from going to facilities.

DISCUSSION

This mixed-method study determined pharmacies to be the most popular source of contraception for young people in a peri-urban area of Kwale County, with 59% of study participants reporting use of contraception purchased at a pharmacy at last sex – substantially higher than previously reported for Kenya as a whole. [9] Multivariable analyses indicated that young people who were still living at home with family relied more heavily on pharmacies for contraception more than their peers. That said, the strongest predictor of young people's contraception coming from pharmacies was the type of contraception they used, specifically emergency contraception. Qualitative findings demonstrated that young people valued pharmacies for their convenience; privacy; non-judgmental and personable staff; service speed; and predictable, affordable prices.

This study had several limitations. In the survey, participants were asked to specify where they or their partner had obtained the contraception used at last sex. This question is standard in studies looking to establish contraception prevalence. However, our not further ascertaining whether it was the

For peer review only - http://bmjopen.bmj.com/site/about/guidelines.xhtml

respondent or their partner *who picked up the contraception* affected our ability to distinguish differences in preferred sources between young men who obtain contraception versus young women who obtain contraception. Second, to recruit young people who had recently purchased contraception from pharmacies, we relied on assistance from five pharmacies, purposively selected (for pharmacists' willingness to cooperate and – for two pharmacies – their high volume of customers). It is possible that young purchasers patronizing different pharmacies might have had very different experiences than those captured here. Finally, our youth participants in focus group discussions may have felt uncomfortable discussing contraceptive use in a group; we attempted to mediate this by structuring discussion around vignettes of 'typical' young people. This study is strengthened by its mixed methods design and its use of multiple qualitative methods, and inclusion of both pharmacy personnel and young people to triangulate research findings on a sensitive subject.

Our findings differ substantially from an analysis of Kenya's DHS data, which found that nationwide, 13% of Kenyan women aged 15-24 reported accessing contraception at a commercial drug seller. [9] There may be several reasons for this, in addition to the four years between the Kenya DHS and our own data collection. Our study area was a peri-urban setting while the DHS analysis uses nationwide data, and over 70% of Kenya's population is rural. [16] Additionally, our study's inclusion of emergency contraception is also a likely contributor: 12% of participants in this study used emergency contraception at last sex, and Kenya's 2014 DHS did not specifically capture emergency contraception use (responses would rather have been grouped under 'other modern method') [4]. How the DHS measures 'current use' of contraception in general has been previously critiqued for not being able to capture contraceptive methods which may be used periodically, including ECP.[17] One final reason is likely our inclusion of young men, who made up 54% of the participants in our analyses. In general, there has been little study of where young men obtain contraception; however, our study's findings are in line with one analysis from a cross-sectional survey of the general British population, which found that among young men aged 16-24, 60% reported using retail sources (including pharmacies and shops) for contraception, and retail sources were the preferred source of contraception for 43% of young men.[18]

The study's qualitative findings on why pharmacies were appealing as sources of contraception for young clients were largely in line with previous research. One systematic review featuring studies mostly from high-income countries (HICs) affirms that young people appreciate pharmacies for their convenience, speed of service and ease of contraception access.[10] However, this review also reported mixed evidence (all from HICs) as to whether pharmacy services were considered 'private'[10], while our study found an almost universal appreciation of pharmacies for their privacy. This difference may be a result of different dispensing protocols and establishment layouts in pharmacies and public health facilities in high-income and LMIC settings, and speaks to the value of LMIC-specific research to improve our understanding of how contraception is delivered through pharmacies in these settings. Additional, preliminary evidence from other LMICs corroborates our findings that among young people[19], and the general population[20], pharmacies' contraception services are appreciated for the privacy offered.

All told, this mixed methods study indicates that pharmacies provide a valued, private source of contraception for many young people who may face increased scrutiny or gatekeeping in health facilities. As compared with providers in health facilities, pharmacy personnel remained non-judgmental in dispensing to young people and did not limit access to contraception (the experience of which might make a client report a less-positive interaction [21]). For young people using condoms or ECP, the reported convenience and speed of service explains a preference for pharmacies. Following unprotected sex, a young person needing ECP would understandably prefer to pay for it at a nearby pharmacy instead of traveling to a health care facility, waiting in line, and speaking with a doctor to obtain it for

For peer review only - http://bmjopen.bmj.com/site/about/guidelines.xhtml

free. These findings are in line with earlier data from urban Kenya, which indicated that upwards of 96% of adult women needing emergency contraception obtained it at a pharmacy.[22] Studies from the UK and the US have noted an additional positive result of this over-the-counter access: fewer hours lapsing between unprotected sex and taking ECP as compared with prescription-only access or clinic access.[23, 24]

While this study focused on pharmacies, its findings also cover perceptions around how contraception services are delivered to young people in public health facilities. Pharmacies were naturally contrasted with health facilities when participants explained young people's preferences and were perceived to be everything that health facilities were not: fast, private and non-limiting. The extras 'procedures' required to obtain contraception in health facilities – which in many cases are unnecessary [25] and have been demonstrated in other settings to limit access[26, 27] - were especially unwelcome for young people, who were uninterested in extended counselling and wary of laboratory tests. As a result, pharmacy services were deemed more 'predictable' than those obtained in health facilities (public or private). Young people could not be certain what they would encounter in health facilities, and one might travel there to find a long line, a familiar face in a queue, an unavailable service, a different doctor, a harsh word, an order for expensive tests. Pharmacies, therefore, offered a more predictable experience in a more convenient location. That, in a country where contraceptive services in public health facilities are free, pharmacy services are appreciated for their comparative low cost speaks to the perceived unpredictability of facility services.

For Kenya, pharmacies are likely to remain a preferred choice of contraception as long as barrier methods and short-acting forms of contraception are popular with young people[4]. It is therefore critical that policymakers recognize the role that private retail pharmacies play as contraception providers to the community, especially its younger members. Finding ways to link the myriad licensed pharmacies to focal points in public health facilities could strengthen a network of accessible and appealing contraception services available to young people. A similar hub-and-spoke approach is used in the implementation of Kenya's broader Community Health Strategy, where community health volunteers are embedded within the community and report back to a facility-based community health extension worker.[28] Additionally, policy dialogues between key pharmacy stakeholders and Ministry of Health could improve the capacity of pharmacy personnel to deliver quality contraceptive services; and strengthen the regulation of the type of services provided. Finally, mandating or incentivizing basic data collection in pharmacies and integrating incoming data with other health data management platforms (in Kenya, the District Health Information System 2 or DHIS2), will improve the accuracy of national and sub-national family planning prevalence measurements and, therefore, the ability to plan for and respond to local contraceptive needs. [29]

It is worth noting that the survey revealed that shops were the second most popular source of contraception for young people. However, our study was not sufficiently powered to be able to analyze shops as a separate subgroup. The reliance on shops and lower-level drug dispensaries is seen elsewhere in the region: one survey in Nigeria found that among young people age 15 to 24, around half sourced their contraception from 'chemists/patent medicine shops' (a cadre of establishment below pharmacies, which does not exist in Kenya).[30] More research is needed to understand how to incorporate these more informal sources into contraception interventions. However the further lack of regulation will make this challenging: lower-level drug dispensers are only peripherally associated with the health system in many settings (in Nigeria, for example, there is no requirement for any health-related training or educational background[31]), while shops are not associated at all.

For peer review only - http://bmjopen.bmj.com/site/about/guidelines.xhtml

Young people in Coastal Kenya steadily rely on pharmacies for contraception and prefer them to health facility services. It should be noted that many of the qualities most appreciated by young participants are also hallmarks of youth-friendly health services, which should be available in both health facilities and pharmacies. [32, 33] Increased collaboration between Ministry of Health and the regulatory Pharmacy and Poisons Board (as well as professional associations of pharmacy personnel) and private retail pharmacies and health facilities at County levels, can exchange operational strengths between both types of providers, and increase the overall network of quality contraception providers for young people.

ACKNOWLEDGEMENTS

We appreciate the support of Jefferson Mwaisaka and Winnie Wangari. Our sincere thanks to all data collectors and participants. The manuscript represents the view of the named authors only.

FUNDING STATEMENT

This work was partially supported by the UNDP/UNFPA/UNICEF/WHO/World Bank Special Programme of Research, Development and Research Training in Human Reproduction (HRP).

COMPETING INTERESTS

None declared

DATA AVAILABILITY STATEMENT

The full deidentified quantitative dataset can be made available on request to corresponding author. Qualitative data cannot be shared publicly, as consent procedures for participants did not include making full interview and focus group discussion transcripts publicly available. However, transcript excerpts are available to researchers on request from the corresponding author and following approval from the University of Nairobi/Kenyatta National Hospital Ethics Committee (contact via uonknh_erc@uonbi.ac.ke).

AUTHOR CONTRIBUTORSHIP

LG conceived of the study and developed the protocol with heavy input from KW and AMH. PG, was Principal Investigator of the AMADILLO study and thereby supported LG in setting up this study's infrastructure in Kenya. LG trained and supervised data collectors, with guidance from PG. JAC and MW developed the statistical analysis plan. LG led the manuscript writing with heavy input from KW and AMH. All authors reviewed and edited drafts.

For peer review only - http://bmjopen.bmj.com/site/about/guidelines.xhtml

REFERENCES

1. Chandra-Mouli V, McCarraher DR, Phillips SJ, Williamson NE, Hainsworth G. Contraception for adolescents in low and middle income countries: needs, barriers, and access. Reproductive Health. 2014;11(1):1.
2. High-Impact Practices in Family Planning (HIPs). Adolescent-friendly contraceptive services: mainstreaming adolescent-friendly elements into existing contraceptive services. . USAID; 2015.
3. MacQuarrie KLD. Unmet need for family planning among young women: Levels and trends. Rockville, MD: ICF International; 2014.
4. Kenya National Bureau of Statistics, Ministry of Health/Kenya, National AIDS Control Council/Kenya, Kenya Medical Research Institute, Population NCf, Development/Kenya. Kenya Demographic and Health Survey 2014. Rockville, MD, USA; 2015.
5. (JSI) JSI. Private Shops and Pharmacies as Providers of Family Planning. 2016.
6. Corroon M, Kebede, E, Spektor G, Speizer I. Key Role of Drug Shops and Pharmacies for Family Planning in Urban Nigeria and Kenya. Global Health: Science and Practice. 2016;4(4):594-609.
7. High-Impact Practices in Family Planning (HIP). Drug Shops and Pharmacies: Sources for family planning commodities and information. Washington DC: USAID; 2013.
8. Ostola J, Corroon M, Speizer I, Kebede E, Spektor G. Private sector distribution of family planning: The role of drug shops and pharmacies in urban Kenya and Nigeria. Measurement, Learning & Evaluation Project for the Urban Reproductive Health Initiative; 2015.
9. Radovich E, Dennis ML, Wong KLM, Ali M, Lynch CA, Cleland J, et al. Who Meets the Contraceptive Needs of Young Women in Sub-Saharan Africa? Journal of Adolescent Health. 2018;62(3):273-80.
10. Gonsalves L, Hindin MJ. Pharmacy provision of sexual and reproductive health commodities to young people: a systematic literature review and synthesis of the evidence. Contraception. 2017;95(4):339-63.
11. Ministry of Health/Kenya. National family planning guidelines for service providers: Updated to reflect the 2009 Medical Eligibility Criteria of the World Health Organization. Nairobi, Kenya: Division of Reproductive Health; 2010.
12. Kwale County Government. Kwale County Integrated Development Plan (2018-2022): Continuing Kwale's Transformation Together 2018.
13. Kenya Ministry of Health. Kwale county: Health at a glance. 2015.
14. Gonsalves L, Hindin MJ, Bayer A, Carcamo CP, Gichangi P, Habib N, et al. Protocol of an open, three-arm, individually randomized trial assessing the effect of delivering sexual and reproductive health information to young people (aged 13–24) in Kenya and Peru via mobile phones: adolescent/youth reproductive mobile access and delivery initiative for love and life outcomes (ARMADILLO) study stage 2. Reproductive Health. 2018;15(1):126.
15. Gonsalves L, Njeri WW, Schroeder M, Mwaisaka J, Gichangi P. Research and Implementation Lessons Learned From a Youth-Targeted Digital Health Randomized Controlled Trial (the ARMADILLO Study). JMIR Mhealth Uhealth. 2019;7(8):e13005.
16. The World Bank. Rural population (% of total population): World Bank staff estimates based on the United Nations Population Division's World Urbanization Prospects: 2018 Revision. New York, USA: World Bank Group; 2018 [Available from: https://data.worldbank.org/indicator/SP.RUR.TOTL.ZS.
17. Marston C, Renedo A, Nyaaba GN, Machiyama K, Tapsoba P, Cleland J. Improving the Measurement of Fertility Regulation Practices: Findings from Qualitative Research in Ghana. Int Perspect Sexual Reprodud Health. 2017;43(3):111-9.

For peer review only - http://bmjopen.bmj.com/site/about/guidelines.xhtml

18. Geary RS, Tomes C, Jones KG, Glasier A, Macdowall W, Datta J, et al. Actual and preferred contraceptive sources among young people: findings from the British National Survey of Sexual Attitudes and Lifestyles. BMJ Open. 2016;6(9):e011966.
19. Cartwright A, Otai J, Maytan-Joneydi A, McGuire C, Sullivan E, Olumide A, et al. Access to family planning for youth: perspectives of young family planning leaders from 40 countries [version 1; peer review: 1 approved with reservations]. Gates Open Research. 2019;3(1513).
20. Peterson J, Brunie A, Diop I, Diop S, Stanback J, Chin-Quee D. Over the counter: The potential for easing pharmacy provision of family planning in urban Senegal [version 3; peer review: 1 approved, 2 approved with reservations]. Gates Open Research. 2019;2(29).
21. Hernandez JH, Mbadu MF, Garcia M, Glover A. The provision of emergency contraception in Kinshasa's private sector pharmacies: experiences of mystery clients. Contraception. 2018;97(1):57-61.
22. International Consortium for Emergency Contraception. Counting what counts: Tracking access to emergency contraception - Kenya. 2013.
23. Rubin AG, Gold MA, Kim Y, Schwarz EB. Use of emergency contraception by US teens: effect of access on promptness of use and satisfaction. Journal of pediatric and adolescent gynecology. 2011;24(5):286-90.
24. Lewington G, Marshall K. Access to emergency hormonal contraception from community pharmacies and family planning clinics. British Journal of Clinical Pharmacology. 2006;61(5):605-8.
25. World Health Organization. Selected practice recommendations for contraceptive use: Third edition. Geneva, Switzerland; 2016.
26. Grossman D, Ellertson C, Abuabara K, Blanchard K, Rivas FT. Barriers to contraceptive use in product labeling and practice guidelines. Am J Public Health. 2006;96(5):791-9.
27. Leeman L. Medical Barriers to Effective Contraception. Obstetrics and Gynecology Clinics of North America. 2007;34(1):19-29.
28. Oliver M, Geniets A, Winters N, Rega I, Mbae SM. What do community health workers have to say about their work, and how can this inform improved programme design? A case study with CHWs within Kenya. Global Health Action. 2015;8(1):27168.
29. Ndiritu M, Anyango R, Ombech E, Mwito CC; Gwer S. Family planning services in Kenya during a transition: Utilization trends across counties (Working Paper). Chapel Hill, NC, USA: MEASURE Evaluation; 2017.
30. Oye-Adeniran BA, Adewole IF, Umoh AV, Oladokun A, Gbadegesin A, Odeyemi KA, et al. Sources of Contraceptive Commodities for Users in Nigeria. PLOS Medicine. 2005;2(11):e306.
31. Oyeyemi AS, Ogunnowo BE, Odukoya OO. Patent Medicine Vendors in Rural Areas of Lagos Nigeria: Compliance with Regulatory Guidelines and Implications for Malaria Control. Tropical Journal of Pharmaceutical Research. 2014;13(1):163-9.
32. Erulkar AS, Onoka CJ, Phiri A;. What is youth-friendly? Adolescents' preferences for reproductive health services in Kenya and Zimbabwe. African Journal of Reproductive Health. 2005;9(3):51-8.
33. World Health Organization. Making health services adolescent friendly: developing national quality standards for adolescent friendly health services. Geneva, Switzerland: World Health Organization; 2012.

For peer review only

For peer review only - http://bmjopen.bmj.com/site/about/guidelines.xhtml

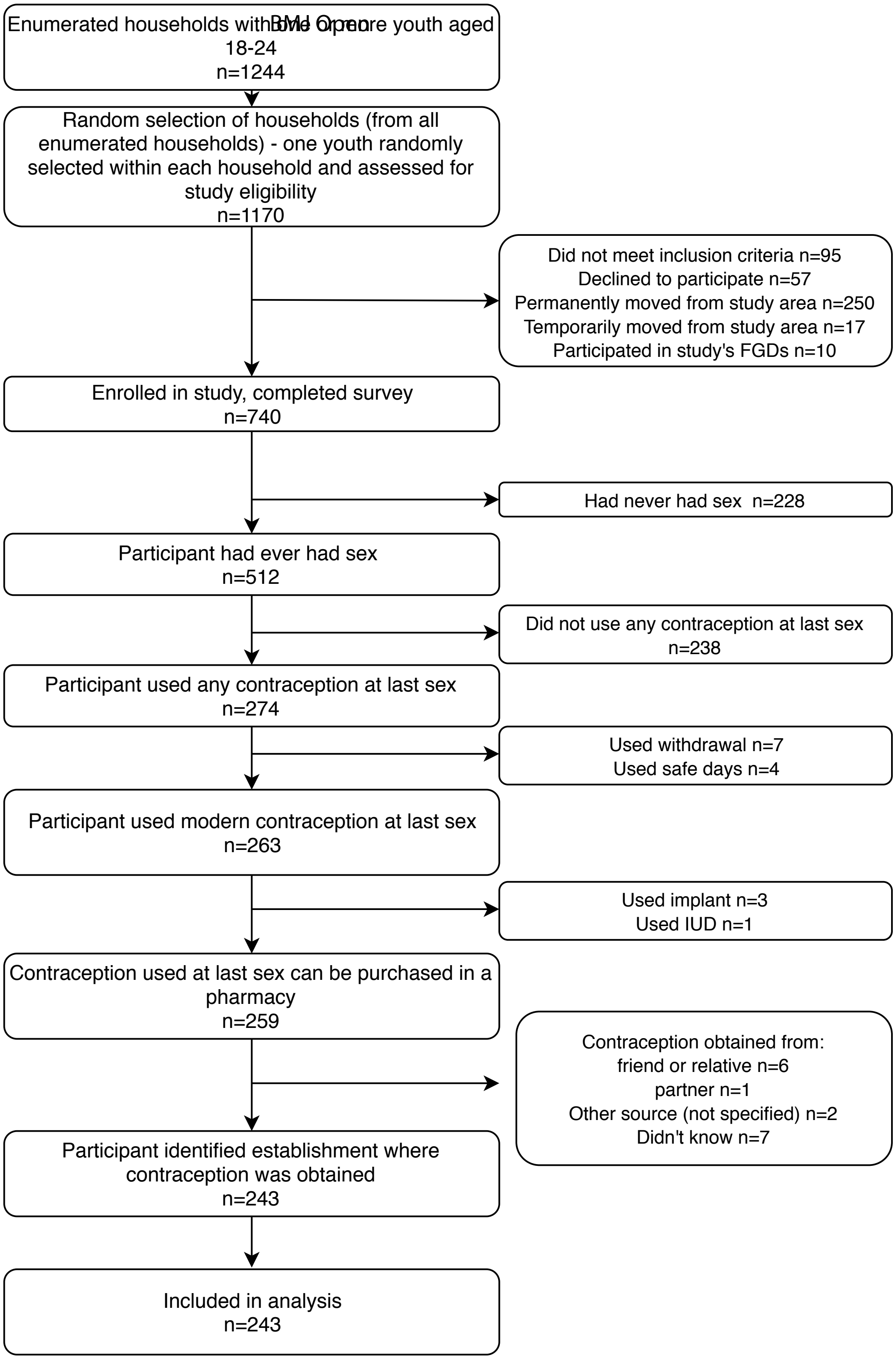
Enumerated households with one or more youth aged 18-24
n=1244
Random selection of households (from all enumerated households) - one youth randomly selected within each household and assessed for study eligibility
n=1170
Did not meet inclusion criteria n=95
Declined to participate n=57
Permanently moved from study area n=250
Temporarily moved from study area n=17
Participated in study's FGDs n=10
Enrolled in study, completed survey
n=740
Had never had sex n=228
Participant had ever had sex
n=512
Did not use any contraception at last sex
n=238
Participant used any contraception at last sex
n=274
Used withdrawal n=7
Used safe days n=4
Participant used modern contraception at last sex
n=263
Used implant n=3
Used IUD n=1
Contraception used at last sex can be purchased in a pharmacy
n=259
Contraception obtained from:
friend or relative n=6
partner n=1
Other source (not specified) n=2
Didn't know n=7
Participant identified establishment where contraception was obtained
n=243
Included in analysis
n=243

Supplementary Table 1 Selected characteristics of young people purchasing contraception at a pharmacy, shop, or any other source

	Pharmacy (N=153)	Shop (N=29)	Any other source (N=61)
Age			
18-19	27 (18%)	6 (21%)	10 (16%)
20+	126 (82%)	23 (79%)	51 (84%)
Sex			
Male	80 (52%)	24 (83%)	28 (46%)
Female	73 (48%)	5 (17%)	33 (54%)
Education			
Primary or below	60 (39%)	7 (24%)	29 (48%)
Secondary or above	93 (61%)	22 (76%)	32 (52%)
Relationship status			
Single	46 (30%)	10 (34%)	25 (41%)
Dating	86 (56%)	18 (62%)	11 (18%)
Cohabiting/Married	21 (14%)	1 (3%)	25 (41%)
Children			
No	139 (91%)	28 (97%)	37 (61%)
Yes	14 (9%)	1 (3%)	24 (39%)
Living situation			
Lives alone	30 (20%)	3 (10%)	6 (10%)
Lives with family (dependent)	113 (74%)	25 (86%)	32 (53%)
Lives with child or partner	10 (7%)	1 (3%)	23 (38%)
Contraception purchased			
Condom	120 (78%)	28 (97%)	33 (54%)
ECP	24 (16%)	1 (3%)	5 (8%)
Pills/Injections	9 (6%)	0 (0%)	23 (38%)

For peer review only

For peer review only - http://bmjopen.bmj.com/site/about/guidelines.xhtml

STROBE Statement—checklist of items that should be included in reports of observational studies

	Item No.	Recommendation	Page No.	Relevant text from manuscript
**Title and abstract**	1	(*a*) Indicate the study's design with a commonly used term in the title or the abstract	1	Title
		(*b*) Provide in the abstract an informative and balanced summary of what was done and what was found	1-2	Abstract
**Introduction**				
Background/rationale	2	Explain the scientific background and rationale for the investigation being reported	3	Paragraph 1-4 of Introduction
Objectives	3	State specific objectives, including any prespecified hypotheses	3	"Therefore, this mixed methods study sought to answer two questions…"
**Methods**				
Study design	4	Present key elements of study design early in the paper	3-4	"This analysis was part of a broader, mixed-methods study describing how young people (aged 18-24) in Kwale County obtain contraception from pharmacies." + Table 1
Setting	5	Describe the setting, locations, and relevant dates, including periods of recruitment, exposure, follow-up, and data collection	4-5	Table 1 + Methods text
Participants	6	(*a*) *Cohort study*—Give the eligibility criteria, and the sources and methods of selection of participants. Describe methods of follow-up<br>*Case-control study*—Give the eligibility criteria, and the sources and methods of case ascertainment and control selection. Give the rationale for the choice of cases and controls<br>*Cross-sectional study*—Give the eligibility criteria, and the sources and methods of selection of participants	4 (cross-sectional)	Table 1, "In October 2017, data collectors enumerated all households…"
		(*b*) *Cohort study*—For matched studies, give matching criteria and number of exposed and unexposed<br>*Case-control study*—For matched studies, give matching criteria and the number of controls per case		N/A

For peer review only - http://bmjopen.bmj.com/site/about/guidelines.xhtml

Variables	7	Clearly define all outcomes, exposures, predictors, potential confounders, and effect modifiers. Give diagnostic criteria, if applicable	6	Analysis section
Data sources/ measurement	8*	For each variable of interest, give sources of data and details of methods of assessment (measurement). Describe comparability of assessment methods if there is more than one group	N/A	Not included beyond primary outcome (to leave space to discuss qual methods)
Bias	9	Describe any efforts to address potential sources of bias	5	"Data collectors entered responses save…"
Study size	10	Explain how the study size was arrived at	4	"The sample size was calculated based on the ARMADILLO trial's primary outcome…"
Quantitative variables	11	Explain how quantitative variables were handled in the analyses. If applicable, describe which groupings were chosen and why	6	Analysis section – for primary outcome
Statistical methods	12	(*a*) Describe all statistical methods, including those used to control for confounding	6	Analysis section
		(*b*) Describe any methods used to examine subgroups and interactions	N/A	Based on primary outcome, no subgroups were examined
		(*c*) Explain how missing data were addressed	N/A	Not included (to leave space to discuss qual methods)
		(*d*) *Cohort study*—If applicable, explain how loss to follow-up was addressed<br>*Case-control study*—If applicable, explain how matching of cases and controls was addressed<br>*Cross-sectional study*—If applicable, describe analytical methods taking account of sampling strategy	N/A	N/A
		(*e*) Describe any sensitivity analyses	N/A	N/A
**Results**				
Participants	13*	(a) Report numbers of individuals at each stage of study—eg numbers potentially eligible, examined for eligibility, confirmed eligible, included in the study, completing follow-up, and analysed	N/A	Not included (to leave space to discuss qual methods – reference describing this in detail is included [15] on page 4)
		(b) Give reasons for non-participation at each stage	N/A	Not applicable (cross-section)

For peer review only

		(c) Consider use of a flow diagram	N/A	Not included (to leave space to discuss qual methods – reference to open source article with this information is included [15] on page 4)
Descriptive data	14*	(a) Give characteristics of study participants (eg demographic, clinical, social) and information on exposures and potential confounders	6-7	Table 2
		(b) Indicate number of participants with missing data for each variable of interest	N/A	None for primary outcome
		(c) *Cohort study*—Summarise follow-up time (eg, average and total amount)		
Outcome data	15*	*Cohort study*—Report numbers of outcome events or summary measures over time		
		Case-control study—Report numbers in each exposure category, or summary measures of exposure		
		Cross-sectional study—Report numbers of outcome events or summary measures	8	Table 3
Main results	16	(*a*) Give unadjusted estimates and, if applicable, confounder-adjusted estimates and their precision (eg, 95% confidence interval). Make clear which confounders were adjusted for and why they were included	8-9	Table 3
		(*b*) Report category boundaries when continuous variables were categorized	8-9	Table 3
		(*c*) If relevant, consider translating estimates of relative risk into absolute risk for a meaningful time period	N/A	N/A
Other analyses	17	Report other analyses done—eg analyses of subgroups and interactions, and sensitivity analyses	N/A	N/A
**Discussion**				
Key results	18	Summarise key results with reference to study objectives	11-12	Discussion paragraph 1
Limitations	19	Discuss limitations of the study, taking into account sources of potential bias or imprecision. Discuss both direction and magnitude of any potential bias	11-12	Discussion paragraph 2
Interpretation	20	Give a cautious overall interpretation of results considering objectives, limitations, multiplicity of analyses, results from similar studies, and other relevant evidence	12	Discussion section paragraph 3-4
Generalisability	21	Discuss the generalisability (external validity) of the study results	12	Discussion section paragraph 3
**Other information**				
Funding	22	Give the source of funding and the role of the funders for the present study and, if applicable, for the original study on which the present article is based	14	Funding statement

For peer review only - http://bmjopen.bmj.com/site/about/guidelines.xhtml
For peer review only

*Give information separately for cases and controls in case-control studies and, if applicable, for exposed and unexposed groups in cohort and cross-sectional studies.

Note: An Explanation and Elaboration article discusses each checklist item and gives methodological background and published examples of transparent reporting. The STROBE checklist is best used in conjunction with this article (freely available on the Web sites of PLoS Medicine at http://www.plosmedicine.org/, Annals of Internal Medicine at http://www.annals.org/, and Epidemiology at http://www.epidem.com/). Information on the STROBE Initiative is available at www.strobe-statement.org.

For peer review only

BMJ Open

Mixed-methods study on pharmacies as contraception providers to Kenyan young people: who uses them and why?

Journal:	*BMJ Open*
Manuscript ID	bmjopen-2019-034769.R1
Article Type:	Original research
Date Submitted by the Author:	24-Jan-2020
Complete List of Authors:	Gonsalves, Lianne; World Health Organization, Department of Sexual and Reproductive Health and Research including UNDP/UNFPA/UNICEF/WHO/World Bank Special Programme of Research, Development and Research Training in Human Reproduction (HRP); Swiss Tropical and Public Health Institute<br>Wyss, Kaspar; Swiss Tropical and Public Health Institute; University of Basel<br>Cresswell , Jenny; World Health Organization, Department of Sexual and Reproductive Health and Research including UNDP/UNFPA/UNICEF/WHO/World Bank Special Programme of Research, Development and Research Training in Human Reproduction (HRP)<br>Waithaka, Michael; International Centre for Reproductive Health Kenya<br>Gichangi, Peter; International Centre for Reproductive Health Kenya; University of Nairobi, Department of Human Anatomy<br>Martin Hilber, Adriane; Swiss Tropical and Public Health Institute; University of Basel
**Primary Subject Heading**:	Sexual health
Secondary Subject Heading:	Respiratory medicine, Public health
Keywords:	REPRODUCTIVE MEDICINE, Community child health < PAEDIATRICS, PUBLIC HEALTH, QUALITATIVE RESEARCH

SCHOLARONE™
Manuscripts

I, the Submitting Author has the right to grant and does grant on behalf of all authors of the Work (as defined in the below author licence), an exclusive licence and/or a non-exclusive licence for contributions from authors who are: i) UK Crown employees; ii) where BMJ has agreed a CC-BY licence shall apply, and/or iii) in accordance with the terms applicable for US Federal Government officers or employees acting as part of their official duties; on a worldwide, perpetual, irrevocable, royalty-free basis to BMJ Publishing Group Ltd ("BMJ") its licensees and where the relevant Journal is co-owned by BMJ to the co-owners of the Journal, to publish the Work in this journal and any other BMJ products and to exploit all rights, as set out in our licence.

The Submitting Author accepts and understands that any supply made under these terms is made by BMJ to the Submitting Author unless you are acting as an employee on behalf of your employer or a postgraduate student of an affiliated institution which is paying any applicable article publishing charge ("APC") for Open Access articles. Where the Submitting Author wishes to make the Work available on an Open Access basis (and intends to pay the relevant APC), the terms of reuse of such Open Access shall be governed by a Creative Commons licence – details of these licences and which Creative Commons licence will apply to this Work are set out in our licence referred to above.

Other than as permitted in any relevant BMJ Author's Self Archiving Policies, I confirm this Work has not been accepted for publication elsewhere, is not being considered for publication elsewhere and does not duplicate material already published. I confirm all authors consent to publication of this Work and authorise the granting of this licence.

For peer review only

Mixed-methods study on pharmacies as contraception providers to Kenyan young people: who uses them and why?

Lianne Gonsalves[123], Kaspar Wyss[23], Jenny A Cresswell[1], Michael Waithaka[4], Peter Gichangi[456], Adriane Martin Hilber[23]

[1]Department of Reproductive Health and Research including UNDP/UNFPA/UNICEF/WHO/World Bank Special Programme of Research, Development and Research Training in Human Reproduction (HRP), World Health Organization, Geneva, Switzerland
[2]Swiss Tropical and Public Health Institute (Swiss TPH), Basel, Switzerland
[3]University of Basel, Basel, Switzerland
[4]International Centre for Reproductive Health Kenya, Mombasa, Kenya
[5]Department of Human Anatomy, University of Nairobi, Nairobi, Kenya
[6]Ghent University, Ghent, Belgium

Corresponding author information:

Lianne Gonsalves
Address: World Health Organization, Avenue Appia 20, 1211 Geneva, Switzerland
Telephone: +41 22 791 29 70
Email address: gonsalvesl@who.int

Word count, excluding title page, abstract, references, figures and tables

3961

Abstract: 272 words

Objectives

This study sought to answer two questions: 1) what are the characteristics of young Kenyans aged 18-24 who use contraception obtained at pharmacies, and 2) why are pharmacies appealing sources of contraception?

Design and Setting

This was a mixed-methods study in one peri-urban part of Kwale County, Kenya. Methods included: cross-sectional survey (N=740); six focus group discussions; 18 in-depth interviews; and 25 key informant interviews. Quantitative data analysis identified factors pushing young people to pharmacies for modern contraception versus other sources. Qualitative data analysis identified reasons pharmacies were perceived to be appealing to young clients.

Participants

Participants were: 1) young people aged 18-24 from the study area, including a subset who had recently purchased contraception from a pharmacy; or 2) pharmacy personnel and pharmacy stakeholders.

For peer review only - http://bmjopen.bmj.com/site/about/guidelines.xhtml

Results

Among surveyed participants who had ever had sexual intercourse and had used modern contraception at last sexual intercourse, 59% obtained it from a pharmacy. In multivariable analysis, participants who used a condom or emergency contraception as well as those living alone were significantly more likely to get contraception from pharmacies. Pharmacies were valued for their: convenience; privacy; non-judgmental and personable staff; service speed; and predictable, affordable prices.

Conclusions

Our findings indicate a high percentage of young people in Coastal Kenya use pharmacies for contraception. Our inclusion of emergency contraception users partially explains this. Pharmacies were perceived to be everything that health facilities are not: fast, private and non-limiting. Policymakers should recognize the role of pharmacies as contraception providers and look for opportunities to link pharmacies to the public health system. This would create a network of accessible and appealing contraception services for young people.

Strengths and limitations of this study

- Participants were asked to specify where they or their partner had obtained the contraception used at last sex. This is a standard question for studies looking to establish contraception prevalence. Our not further ascertaining who specifically obtained the contraception affected our ability to distinguish differences in preferences of young men versus young women.
- One participant group (young people who had recently purchased contraception from a pharmacy) was recruited from five purposively selected pharmacies: this may limit the generalizability of the findings.
- This study is strengthened by its mixed methods design and inclusion of both pharmacy personnel and young people to triangulate research findings on a sensitive subject.

For peer review only - http://bmjopen.bmj.com/site/about/guidelines.xhtml

INTRODUCTION

Young people need access to contraception. However, around the world, and in low- and middle-income countries in particular, public sector contraceptive services are not meeting this need. Data from 61 low- and middle-income countries estimated that 33 million young women aged 15-24 had an unmet need for family planning.[1] Adolescents (ages 10-19 years) and youth (15-24 years) are often reluctant to access contraception at public health facilities where they may encounter a lack of privacy, biased providers, and limited contraceptive options, in addition to broader financial, legal, social, and cultural barriers. [2, 3]

Other parts of the health system may be able to step in to help fill this gap. In Kenya (where this study took place) and in the region, private pharmacies have become a source of modern contraception for young people [4-7]. Additional research has indicated that when contraception is introduced in pharmacies, access improved for young people.[8, 9] An analysis of 33 sub-Saharan African countries found that commercial drug sellers, including pharmacies, were the source of the most recent contraceptive method for nearly one in five young people between 15-24 years of age. [8] When also factoring in other informal and non-medical providers, including shops, these sources together serviced nearly half of women age 15-19. [8]

Kenya's National Family Planning Guidelines allow for the provision of several kinds of modern methods [10] of contraception to be dispensed by pharmacists or pharmaceutical technologists [11](colloquially referred to as 'chemists'). These include barrier methods like male and female condoms, as well as short-acting methods including emergency contraception (ECP), oral contraceptive pills, and injectable contraception. Injectables can be dispensed but not administered. These permissions mean that outside of health facilities, private retail pharmacies have the largest selection of modern methods available (shopkeepers can also sell condoms, per the guidelines). Private retail pharmacies must be opened and should always operate under the supervision of either a pharmacist or pharmaceutical technologist.[12]

Despite their demonstrable popularity among young people, there is little data on the individual-level circumstances or characteristics of young people that would drive them to pharmacies for contraception. Therefore, this mixed methods study sought to answer two questions: 1) what are the characteristics of young people who use contraception obtained at pharmacies, and 2) why are pharmacies appealing sources of contraception to young people?

METHODS

This analysis was part of a broader, mixed-methods study describing how young people (aged 18-24) in Kwale County obtain contraception from pharmacies. Kwale County is one of six counties in Kenya's former Coast region. The study took place in the peri-urban areas of Kwale Town and Ukunda, as well as the stretch of highway connecting the two towns. Young people between the ages of 15-24 were projected to make up 19% of the county's population by 2018.[13] In 2014, contraception prevalence in the county was 38%, lower than the national average of 53%. [14]

Data collection took place between October 2017 and March 2018. We used several methods (captured in Table 1) to understand the experiences of pharmacy personnel and young people themselves. This study was partly-nested in the ARMADILLO randomized controlled trial (RCT)[15], which assessed the effect of an unrelated digital health intervention on sexual and reproductive health-related outcomes for young people aged 18-24.

Table 1 Study Methods

For peer review only - http://bmjopen.bmj.com/site/about/guidelines.xhtml

Method	N	Eligibility criteria	Relevant topics addressed
**Cross-sectional survey***	**740**	• Age 18-24<br>• Literate<br>• Have their own mobile phone (with them at time of recruitment) and report regular use<br>• Report current use of text messaging	• Contraception used at last sexual intercourse and source<br>• Demographic and behavioral characteristics
**Focus group discussions***	**6 (58 participants)**	• Age 18-24<br>• Community members	• Sources of contraception for young people<br>• Characteristics of young people who use each source
**In-depth interviews**	**18**	• Age 18-24<br>• Recently purchased contraception at pharmacy	• Reasons for having purchased contraception from pharmacy<br>• What was valued (and not valued) about experience
**Key-informant interviews**	**19 (pharmacy personnel)<br>6 (stakeholders)**	• Age 18+<br>• Pharmacy personnel (any role) OR<br>• Pharmacy-related stakeholder (Ministry of Health; regulatory agency; professional association; non-governmental organization)	• Characteristics of young people who purchase contraception<br>• What clients appreciate about experience

* Methods which were nested in the broader ARMADILLO Study, a digital health intervention RCT. Inclusion/exclusion criteria for these nested methods were determined by ARMADILLO's objectives.

To capture the perspectives of young people, a cross-sectional survey of young people age 18-24 captured demographic information and contraceptive use patterns, including source of last contraception (these questions were one section of a broader survey conducted as part of the baseline assessment for the ARMADILLO trial). The sample size was calculated based on the ARMADILLO trial's primary outcome – the full protocol for the trial has been previously published[15], along with details of participants recruited.[16]

To identify participants, we obtained a map of the study area from the Kenya National Bureau of Statistics. The KNBS the country into so-called 'enumeration areas' (EAs) in preparation for the country's 2019 census. EAs consist of blocks of households. Each EA had approximately 100 households. In October 2017, data collectors enumerated all age-eligible young people in every household using a random selection of 21 EAs in the study area. From this list of age-eligible youth, a random selection of households and random selection of one youth per household was generated. Data collectors visited the selected households to recruit participants (who met eligibility criteria captured in Table 1) starting in February 2018.

Additionally, six Focus Group Discussions were conducted with young people age 18-24, purposively recruited from the community by data collectors. Finally, we conducted in-depth interviews with 18

For peer review only

young people aged 18-24 who had recently purchased contraception from pharmacies. We purposively recruited these young participants in one of two ways. First, we stationed a young data collector outside of well-trafficked pharmacies over three evenings, who recruited young people purchasing contraception. Second, several pharmacists in the study area were provided with leaflets with study information and requested to provide these to young contraception purchasers at the end of a transaction.

To capture the perspectives of pharmacy personnel, data collectors mapped all private, retail pharmacies in the study area using a digital form with an embedded geolocator. A random subset of pharmacies was generated using the random number generator in Excel. Pharmacies were well-distributed across the study area. In each selected pharmacies, data collectors were instructed to approach the first person behind the counter, regardless of rank or level of training, explain the study and ask if they would be interested in participating. Nineteen interviews in total were conducted. An additional six key-informant interviews were conducted with stakeholders from the regulatory Pharmacy and Poisons Board, Ministry of Health, professional associations, and non-governmental organizations. These were conducted in the individuals' offices in either Ukunda, Mombasa, or Nairobi. Stakeholder participants were contacted first by phone or email, the studied explained, and a convenient time for an in-person visit set.

Data collection and management

We obtained informed consent from all participants prior to participation. All data was collected in English, Swahili, or a mix of the two, depending on participants' preference. Quantitative surveys were close-ended and administered using webforms on a tablet. Data collectors entered responses save for the questions related to participants' sexual and contraceptive use history; here, to reduce potential discomfort and response bias, participants entered their own responses. Interviews and FGDs used semi-structured guides: FGD (S1), in-depth interview (S2), and key-informant interview (S3), in addition to relevant survey components (S4) are provided as supplementary material. We adopted an iterative approach to data collection, allowing question guides to be modified based on emerging themes. Qualitative data collection ceased upon reaching saturation. All qualitative methods used audio-recording (with participant permission). All study activities were conducted in a private location. Data collectors, speaking both English and Swahili, were recruited from the study area and specifically trained for this study.

This study received ethics approval from the Ethikkommission Nordwest- und Zentralschweiz (EKNZ) (Req-2017-00389) in Basel, Switzerland, as well as the University of Nairobi/Kenyatta National Hospital in Nairobi, Kenya (P274/05/2017). The ARMADILLO RCT also received ethics approval from the World Health Organization (Protocol WHO A65892) and is registered with the ISRCTN Registry (ISRCTN85156148).

Patient and public involvement

Our population (young people) were directly involved in parts of the study's design and implementation. Our survey data collection team consisted of young people recruited from the study area (Kwale County). Qualitative method data collectors were also young people recruited from both Kwale and Mombasa Counties. We relied on their insight and lived experience to determine how young people would feel most comfortable being recruited. We jointly designed our recruitment and consenting procedures. A dissemination meeting involving local, county, and national stakeholders (including some pharmacy stakeholder participants) took place in June, 2019. Several young data collectors were invited to attend and they provided commentary on the findings.

For peer review only - http://bmjopen.bmj.com/site/about/guidelines.xhtml

Researcher characteristics and reflexivity

Data collectors were young people (nearly even numbers of men and women – 24 in total) recruited from Kwale and Mombasa counties. Kwale County data collectors were familiar with the study area and recognized within their communities, which facilitated enumerating pharmacies, recruiting youth participants, and getting consent to interview pharmacy personnel. They were also less educated and less experienced than data collectors from Mombasa County. This, at times, resulted in a subordinate dynamic with some pharmacy personnel participants who were university-educated. The first author conducted all interviews with pharmacy stakeholders. She is from the United States (from a racial minority group different from the study population) and presented as an outsider (someone not from Kenya) to interviewees. Her position (leading the study and professional affiliations) resulted in respondents treating her collegially and being open to participate.

Analysis

Quantitative data was analyzed in Stata Version 14. The subject of the analyses (as described in Figure 1) were survey participants who reported using one of four contraception commodities available in pharmacies (either male or female condom, ECP, daily contraceptive pills, or injectable contraception) at last sexual intercourse *and* who reported their source. Sexual intercourse was presumed to be penetrative vaginal sex. Excluded were those participants who had not used contraception at last sexual intercourse, who had not used a contraceptive commodity (withdrawal method, calendar days), who could not remember where they had obtained their method and/or who had obtained it from a partner or friend. We developed a dichotomous 'source of family planning' outcome, distinguishing between 'pharmacy' and 'any other source'. The latter included any public or private health facility, community-based distributors, non-governmental organizations, shops, schools, supermarkets. Following descriptive statistics, bivariate log binomial regressions assessed the association between the outcome and each behavioral/sociodemographic variable of interest. Any analysis showing a $p<.2$ moved the variable into a multivariable Poisson regression model with robust 95% CIs.

Figure 1 Flow Diagram of Study Participants

All qualitative data was transcribed verbatim and then translated (if necessary) into English. For a sub-section of Swahili-language interviews, English-language transcripts were compared against the original Swahili-language interview audio file by another member of the research team to ensure consistency. Qualitative analysis for the broader study was guided by the five, *WHO-defined dimensions of quality health services to adolescents*: equity, accessibility, acceptability, appropriateness, and effectiveness. [17] Qualitative analysis was conducted in Atlas.ti Version 8 and relied on thematic analysis, with deductive and then inductive coding of a subset of transcripts to develop and refine a coding framework. Deductive coding was informed by the 'accessibility' and 'acceptability' dimensions and broadly captured any reference to pharmacies being 'appealing'. Inductive coding of these data then identified specific reasons for appeal, subsequently grouping these into broad categories related to pharmacy outlet, personnel, and service appeal. These broad categories and individual reasons structure the presentation of the qualitative results.

RESULTS

Survey sample characteristics

A total of 1170 youth were approached for participation, of which 740 (63%) consented to participate and completed the survey. Reasons for non-participation are captured in Figure 1. As seen in Table 2, of

For peer review only - http://bmjopen.bmj.com/site/about/guidelines.xhtml

the 740 young people aged 18-24 who participated in the cross-sectional survey, 512 (69%) had ever had sexual intercourse. Male condoms were the most popular form of contraception purchased, used by 190 of the 274 (69%) participants who used contraception at last sexual intercourse (hereafter 'at last sex'). Of the participants indicating that they used a modern contraceptive at last sex (N=263), 154 (59%, data not shown) had obtained it from a private, retail pharmacy (hereafter, 'pharmacy').

Table 2 Baseline characteristics

	All surveyed participants (N=740)		
	Female	Male	Total
Ever had sexual intercourse	231/347	281/393	512/740 (69%)
Used any contraception at last sex	126/231 (55%)	148/281 (53%)	274/512 (54%)
Used a modern contraceptive at last sex	118/231 (51%)	145/281 (52%)	263/512(51%)
Used pharmacy-available contraception*	116/231 (50%)	143/281 (51%)	259/512 (51%)
Where contraception was obtained	(N=116)	(N=143)	(N=259)
Pharmacy	63%	56%	59%
Shop	5%	17%	11%
Public dispensary or health centre	13%	7%	10%
Hospital	11%	6%	8%
NGO, private doctor	3%	4%	4%
Community-based distributor, school, supermarket	1%	2%	2%
*Other person***	1%	4%	*3%*
*Other source (not specified)/Don't know ***	3%	3%	*3%*
	**Included participants using pharmacy-available contraception (N=243)**		
	Female (N=111)	Male (N=132)	Total (N=243)
Age			
18-19	17%	18%	18%
20-24	83%	82%	82%
Education (highest level attended)			
Primary or below	54%	27%	40%
Secondary	38%	55%	47%
Post-secondary	8%	18%	14%
Relationship status			
Single	23%	42%	33%
Friends with benefits	3%	8%	5%
Dating	42%	42%	42%
Cohabiting	3%	1%	2%
Engaged	9%	5%	7%
Married	20%	3%	11%
Any children			
No	74%	92%	84%
Yes	26%	8%	16%
Living situation			
Lives alone	8%	23%	16%
Lives with family (dependent)	66%	73%	70%
Lives with child or partner	26%	4%	14%

For peer review only

For peer review only - http://bmjopen.bmj.com/site/about/guidelines.xhtml

Contraception used***			
Male condom	56%	86%	72%
Female condom	4%	2%	2%
ECP	20%	6%	12%
Daily contraceptive pills	5%	2%	3%
Injection	16%	5%	10%

*these included male or female condom, emergency contraception (ECP), daily contraceptive pills, and injectable contraception

**these were excluded from analysis

***Participants could enter one contraceptive method

Of the 243 participants who were included in bivariate and multivariable analyses, 54% were male, 61% had attended secondary school or higher, and 70% were dependents (living with parents, grandparents, or other older family members). A higher proportion of female participants than male participants were cohabiting, engaged, or married and had at least one child. Male participants had attended higher levels of schooling than female participants. Supplementary Table 1 presents selected characteristics of the 243 participants disaggregated by whether they obtained contraception at a pharmacy, shop, or any other source: most shop users were male and purchased condoms.

Who accesses contraception from pharmacies?

Bivariate analyses (Table 3) indicated there was no evidence of an association between either age, sex, or education and a young person's contraception being from a pharmacy. There was an association between pharmacy-purchased contraception and a participant's relationship status, and whether they had children. The greatest predictors of whether a young person had visited a pharmacy were the type of contraception they purchased and with whom they lived. Following multivariate analysis (Table 3), there remained strong evidence of an association between pharmacy purchase of contraception and a young person's relationship status, living situation, as well as the type of contraception they used. Young people living alone were almost twice as likely to have sourced contraception from a pharmacy as those living with a child or partner (Adjusted PR 1.96, 95% CI [1.07-3.59]). Use of ECP remained the greatest predictor of a pharmacy purchase (Adjusted PR 2.27 as compared with pill/injection use 95% CI [1.21-4.27]).

Table 3 Bivariate and multivariable analysis to identify personal characteristics that may be associated with a young person obtaining contraception from a pharmacy (vs any other source)

	Purchased contraception from pharmacy	Unadjusted Prevalence Ratio (PR) [95% CI]	p-value*	Adjusted Prevalence Ratio (PR) [95% CI]	p-value
All	153/243 (63%)				
Age					
18-19	27/43 (63%)	Ref			
20-24	126/200 (63%)	1.00 [0.78-1.29]	0.979		
Sex					
Male	80/132 (61%)	Ref			
Female	73/111 (66%)	1.09 [0.90-1.32]	0.405		
Education					
Primary or below	60/96 (63%)	Ref			

For peer review only - http://bmjopen.bmj.com/site/about/guidelines.xhtml

Secondary or above	93/147 (63%)	1.01 [0.83-1.23]	0.904		
Relationship status					
Single	46/81 (57%)	0.76 [0.61-0.94]	0.0013	0.75 [0.61-0.93]	0.0284
Dating/'Friends with benefits'	86/115 (75%)	Ref		Ref	
Married/Engaged/Cohabiting	21/47 (45%)	0.60 [0.43-0.84]		0.95 [0.67-1.35]	
Children					
No	139/204 (68%)	1.89 [1.24-2.92]	0.003	1.25 [0.80-1.97]	0.318
Yes	14/39 (36%)	Ref		Ref	
Living situation					
Lives alone	30/39 (77%)	2.62 [1.51-4.53]	0.0024	1.96 [1.07-3.59]	0.0119
Lives with family (dependent)	113/170 (66%)	2.26 [1.33-3.85]		1.53 [0.84-2.82]	
Lives with child or partner	10/34 (29%)	Ref		Ref	
Contraception used					
Condom (m/f)	120/181 (66%)	2.36 [1.34-4.14]	0.0014	1.87 [1.02- 3.43]	0.0224
ECP	24/30 (80%)	2.84 [1.59-5.09]		2.27 [1.21-4.27]	
Pills/Injection	9/32 (28%)	Ref		Ref	

*any variable with p-values <.2 in bivariate analysis were included in the multivariable analysis

Qualitative methods participant characteristics

Three FGDs were held with young men, and three with young women – each FGD had approximately ten participants. Of the 18 in-depth interview participants, ten were young women and eight were young men. Female IDI participants had most recently purchased emergency contraception (n=7), injection (n=2), and condom (n=1). Male IDI participants had most recently purchased condom (n=6), and emergency contraception (n=2).

Of the 19 key informant participants, 10 interviewed pharmacy personnel were women, 9 were men. Participants were not probed in detail on their formal training (and therefore whether they should be operating in their current role). That said, we could ascertain that 13 of the participants had an appropriate amount of training for their reported tasks, and four did not (two were unclear). Self-reported education ranged from having some secondary education to full training as a pharmacist or pharmaceutical technologist. One participant was a nurse. Stakeholders demographics are not described to ensure they remain unidentifiable.

Why are pharmacies appealing?

Participants indicated that it was a combination of the pharmacy *outlet,* the pharmacy *personnel* themselves, and the *services* provided by the pharmacy which together made these establishments the preferred source of contraception for many young people (Table 4).

Table 4 Reasons why pharmacies are appealing (selected excerpts from qualitative data)

**Outlet appeal**	*The physical pharmacy environment and its operation*
Convenience (locations and hours)	"The chemist is near and whenever you want it [family planning] you can access it, anytime." Female pharmacy purchaser: injection

For peer review only

For peer review only - http://bmjopen.bmj.com/site/about/guidelines.xhtml

	"The good thing with chemist is that they are many of them…when you missed a certain contraceptive at a certain chemist you can go to the next chemist because they are several of them, not like the hospital" – Female community member (FGD)<br><br>"Yes, majority of them [young people] don't live near health centres. Second, health centres are usually busy. And it's not every day they [can be] attended to: there are specific days they have clinics… [The client] won't be able to make it there…even if the treatment was free. But there is a chemist - [they] can go for similar services." – Pharmacist
Anonymity	"At the chemist there are not many people. I may go to Diani dispensary [a local public health facility], and there is someone who knows me and I go for family planning. I saw it would be better to go the chemist because I know that will be my secret and the attendant." Female pharmacy purchaser: emergency contraception<br><br>"When you go to the facility, when you go to the FP room, everyone knows that you've gone to get FP. For young people [especially] because no one will want to see me - I'm 18, I'm 16 and I'm already using family planning. I'm not supposed to be sexually active. The kind of population that is in those FP areas, around those FP areas it's your mothers who are either breastfeeding, or they're pregnant and have gone for ANC." – Ministry of Health official, County level
**Personnel appeal**	*The person behind the counter*
Interpersonal relationship	"the chemist is just within the neighborhood and I know the guy he is my friend outside job so it wasn't stressful for me in fact it was really fast and easy." – Male pharmacy purchaser: ECP and condoms<br><br>"The person in charge is my friend, I can go to him with my problems and he would assist me, he is not that far for me to reach him with my phone - he is my neighbor I could have a problem even at night and be able to reach out to him." -Male pharmacy purchaser: ECP
Seen as part of the community	"I chose it because it has been there for many years even before I was born till the time I finished school. The attendants are just normal. Many people get help from there so I saw it good to also go there." – Female pharmacy purchaser: ECP and injection<br><br>"What I had said about the hospital, when you get there you will find the person who served you before is transferred but when you come to the chemist you will find the person that served you before." – Female community member (FGD)
Non-judgmental	"I thought at the chemist they will understand me and I would talk to them [better] than at the hospital where they will say I do not need to use those things or even talk to me harshly." –Male pharmacy purchaser: ECP and condoms<br><br>"At the chemist, that person wants - since it is a business – [to] just give, as compared to the hospital where when you get there you will find nurses who are arrogant or other doctors who will insult you." Male community member (FGD)
**Service appeal**	*The contraception-purchasing transaction*
Speed	You know at the dispensary it is a must you meet with the doctor for more explanation. And maybe there is a service you need to pay for, the expenses are

For peer review only - http://bmjopen.bmj.com/site/about/guidelines.xhtml

	many at the dispensary unlike the chemist where everything is fast, when you get there you get what you want and leave. – Youth female, has purchased ECP and condoms<br><br>"You get in a hospital, there are so many people queueing outside that are waiting to see a doctor. Here comes a young lady who is in a hurry. That particular person will find it more convenient to go to a chemist shop rather than going to a hospital." – Pharmacist
Cost	It is not easy for the government hospital. It is best, if you have money, you go to private hospitals. Now that is why you see if someone does not have money, or us the young people, we just go to the chemist because there is no cash to see a doctor for Ksh 600. At the chemist you just go direct and you are served. – Male pharmacy purchaser: ECP and condoms<br><br>Chemists are not expensive like hospitals. In hospital you can be told it is a government hospital but you end up being asked to give out a lot of money. In [the] chemist the money you get asked is for[paying for] P2 [an emergency contraceptive], yah but in hospital you will be told to do some test because we think it is this and this.– Female pharmacy purchaser: ECP<br><br>Free does not always mean free. Sometimes, something will be free, but by the time you get it, the process is a lot. Because for us, we don't just offer family planning, we do [mandatory] counselling. The person who is going to a chemist is someone who has made up his or her mind. But in the public facilities, you are counselled, you are explained to, you are told the different methods, then you are given a chance to make an informed choice. So, I think that…is a barrier somehow. – Ministry of Health Official, County level

Pharmacy outlets were appealing because of the convenience and anonymity they offered young clients. Pharmacies were located where young people lived, worked, and spent time, making them easy contraception access points. If one pharmacy lacked what a young person was looking for, it was a short trip to the next one. 'Convenience' also extended to the days and hours pharmacies were open. This made them especially important on days where health facilities were known to be busy, or evening and weekend hours when young people might need contraception.

Additionally, the relative privacy offered by pharmacies was especially important to young clients. Participants perceived pharmacies, with interactions limited to a pharmacy attendant and a client, to be far more discreet than similar services offered at public health facilities. Public health facilities had public waiting areas where young people may see someone they knew. Additionally, services in the health facility might be categorized by service type (for example, contraceptive services separated from immunization services, etc). This left young clients feeling particularly exposed should they need to walk up to a labeled 'family planning' window or step forward if a public announcement about contraceptive services was made.

The individuals behind the counter, and how they interacted with young people, were additional reasons young people preferred to obtain contraception from pharmacies. Pharmacy personnel were perceived to be established, fellow community members. Young clients appreciated seeing the same familiar faces, with less of the personnel turnover associated with public health facilities. When personnel were a similar age to young clients (a very strong preference of all young participants), many

For peer review only - http://bmjopen.bmj.com/site/about/guidelines.xhtml

reported being able to communicate openly with pharmacy personnel and being more comfortable interacting with them.

Pharmacy personnel were perceived to be non-judgmental compared with those working in health facilities. There was a perception that a trip to a facility would result in difficult questions, and a possible refusal to provide the desired contraceptive. Pharmacy personnel, by contrast, would treat young people well. That is, they would provide the desired contraceptive without interrogation. Several participants speculated that the for-profit aspect of pharmacies could be a reason that they were treated better and not refused services.

Finally, pharmacy contraception services themselves were appreciated for being fast and cheap. Participants routinely referenced the queueing for services and long wait times driving young people away from health facilities and into pharmacies instead. Services were also perceived to be cheaper than both private health facility services as well as public health facility services. Private health facilities were considered out of financial reach for most young people – making a chemist a more affordable option. However, at public health facilities, where contraception-related services are meant to be free, participants indicated that this was often not the case in practice. Expenses related to travel, or 'tests' (for example, a pregnancy test) ordered by health care providers prior to dispensing contraception made real costs related to public services difficult to predict. Finally, as one government official acknowledged, even when services were free, the time and processes required could deter young people who knew what they wanted from going to facilities.

DISCUSSION

This mixed-method study determined pharmacies to be the most popular source of contraception for young people in a peri-urban area of Kwale County. In total, 59% of participants (and 63% female participants) who had ever had sex and self-reported use of a modern contraceptive at last sex had obtained their contraception from a pharmacy. This is higher than previously reported for Kenya as a whole. [8] Multivariable analyses indicated that young people who were still living at home with family relied more heavily on pharmacies for contraception more than their peers. That said, the strongest predictor of young people's contraception coming from pharmacies was the type of contraception they used, specifically emergency contraception. Qualitative findings demonstrated that young people valued pharmacies for their convenience, anonymity, non-judgmental and personable staff, service speed, as well as predictable and affordable prices.

Together, these mixed methods indicate that pharmacies provide a valued source of contraception for those young people who may face increased scrutiny or gatekeeping in health facilities. For young people using condoms or ECP, the reported convenience and speed of service explains the strong preference for pharmacies. Following unprotected sex, a young person needing ECP would understandably prefer to pay for it at a nearby pharmacy instead of traveling to a health care facility, waiting in line, and negotiating with a possibly reluctant health worker to obtain it for free (assuming the public facility stocked ECP [18]).

This study had several limitations. In the survey, participants were asked to specify where they or their partner had obtained the contraception used at last sex. This question is standard in studies looking to establish contraception prevalence. However, our not further ascertaining whether it was the respondent or their partner *who picked up the contraception* affected our ability to distinguish differences in preferred sources between young men who obtain contraception versus young women who obtain contraception. Second, to recruit young people who had recently purchased contraception from pharmacies, we relied on assistance from five pharmacies, purposively selected. It is possible that

For peer review only - http://bmjopen.bmj.com/site/about/guidelines.xhtml

young purchasers patronizing different pharmacies might have had different experiences than those captured here. Finally, our youth participants in focus group discussions may have felt uncomfortable discussing contraceptive use in a group; we attempted to mediate this by structuring discussion around vignettes of 'typical' young people. This study is strengthened by its mixed methods design and its use of multiple qualitative methods, and inclusion of both pharmacy persaonnel and young people to triangulate research findings on a sensitive subject.

Our quantitative findings differ substantially from an analysis of Kenya's DHS (KDHS) data, which found that nationwide, 13% of Kenyan women aged 15-24 currently using contraception reported obtaining it at a commercial drug seller. [8] There may be several reasons for this, in addition to the four years between the KDHS and our own data collection. Our study area was a peri-urban setting while the DHS analysis uses nationwide data. Over 70% of Kenya's population is rural. [19] Finally, our study's inclusion of emergency contraception and measuring contraception use at last sex (rather than 'current use') is also a likely contributor. Twelve percent of participants in this study used emergency contraception at last sex, and the KDHS did not specifically capture emergency contraception use [20]. The DHS's measures of contraception 'current use' in general has been previously critiqued for not being able to capture contraceptive methods which may be used periodically, including ECP.[21] Our link between ECP purchasers and pharmacies are in line with earlier data from urban Kenya, which indicated that upwards of 96% of adult women needing emergency contraception obtained it at a pharmacy.[22]

By contrast, our qualitative findings were largely in line with previous research. One systematic review featuring studies mostly from high-income countries (HICs) affirms that young people appreciate pharmacies for their convenience, speed of service and ease of contraception access.[9] However, this review also reported mixed evidence (all from HICs) as to whether pharmacy services were considered 'private'[9], while our study found an almost universal appreciation of pharmacies for their anonymity/privacy. This difference may be a result of different dispensing protocols and establishment layouts in pharmacies and public health facilities in HICs vs LMICs. Preliminary evidence from other LMICs corroborates our findings that among young people[23], and the general population[24], pharmacies' contraception services are appreciated for the privacy offered.

While this study focused on pharmacies, its findings also cover perceptions around how contraception services are delivered to young people in public health facilities. Pharmacies were naturally contrasted with health facilities when participants explained young people's preferences and were perceived to be everything that health facilities were not: fast, private and non-limiting. The extra 'procedures' required to obtain contraception in health facilities – which in many cases are unnecessary [25] and have been demonstrated in other settings to limit access[26, 27] - were especially unwelcome for young people, who were uninterested in extended counselling and wary of laboratory tests. As a result, pharmacy services were deemed more 'predictable' than those obtained in health facilities (public or private).

For Kenya, pharmacies are likely to remain a preferred choice of contraception as long as barrier methods and short-acting forms of contraception are popular with young people[20]. Policymakers should therefore recognize their role as contraception providers, especially for a community's younger members. Finding ways to link the myriad licensed pharmacies to focal points in public health facilities could strengthen a supportive 'network' of accessible and appealing contraception services available to young people. A similar hub-and-spoke approach is used in the implementation of Kenya's broader Community Health Strategy, where community health volunteers are embedded within the community and report back to a facility-based community health extension worker.[28] Such a system, complemented by improved adolescent-friendliness of public health facilities, would also enable easier referral of young people to providers who can offer them more effective forms of contraception.

For peer review only - http://bmjopen.bmj.com/site/about/guidelines.xhtml

However none of this can succeed without taking needed steps to improve pharmacy regulation, personnel training, and the overall quality of services.[29]

Our data revealed that shops were the second most popular source of contraception for young men. The reliance on shops and lower-level drug dispensaries is seen elsewhere in the region: one survey in Nigeria found that among young people age 15 to 24, around half sourced their contraception from 'chemists/patent medicine shops' (a cadre of establishment below pharmacies, which does not exist in Kenya).[30] Unfortunately, exploring shops in further detail was beyond the scope of our data collection. Additional research is needed to understand how to incorporate these more informal sources into contraception interventions. That said, integrating these source into the broader 'network' of contraception providers for young people will be even more challenging: lower-level drug dispensers are only peripherally associated with the health system in many settings, while shops are not associated at all.

Finally, we must acknowledge those still left behind. Of participants who reported ever having sex, almost half of them (49%) had *not* used any modern contraception at last sex. These are young people who are not being reached by the current network of public and private health facilities, pharmacies, and even neighborhood shops. They are a reminder that improving the quality of services in these outlets is necessary but not sufficient to addressing young people's contraceptive needs. There is a continued need for multi-sectoral interventions, including comprehensive sexuality education, to increase demand for contraception among youth (dispelling myths, addressing taboos and stigma, and increasing agency) [31], address barriers to accessing it (including community norms around acceptability) [3], and promote uptake of highly effective forms of contraception.

Young people in Coastal Kenya steadily rely on pharmacies for contraception and often prefer them to health facility services. Many of the pharmacy qualities most appreciated by young participants are also hallmarks of youth-friendly health services, which should be available in any outlet a young person accesses health services. [17, 32] If a young person chooses to use modern contraception, their selection of an outlet will be determined by several factors, including the type of contraception desired, living situation, and relationship status. Collaboration between health facilities and retail pharmacies at local levels can exchange operational strengths between these providers. Then, wherever a young person presents for contraceptive services, they encounter one part of a supportive network of quality providers.

For peer review only

ACKNOWLEDGEMENTS

We appreciate the support of Jefferson Mwaisaka and Winnie Wangari. Our sincere thanks to all data collectors and participants. The manuscript represents the view of the named authors only.

FUNDING STATEMENT

This work was partially supported by the UNDP/UNFPA/UNICEF/WHO/World Bank Special Programme of Research, Development and Research Training in Human Reproduction (HRP).

COMPETING INTERESTS

None declared

For peer review only - http://bmjopen.bmj.com/site/about/guidelines.xhtml

DATA AVAILABILITY STATEMENT

The full deidentified quantitative dataset can be made available on request to corresponding author. Qualitative data cannot be shared publicly, as consent procedures for participants did not include making full interview and focus group discussion transcripts publicly available. However, transcript excerpts are available to researchers on request from the corresponding author and following approval from the University of Nairobi/Kenyatta National Hospital Ethics Committee (contact via uonknh_erc@uonbi.ac.ke).

AUTHOR CONTRIBUTORSHIP

LG conceived of the study and developed the protocol with heavy input from KW and AMH. PG, was Principal Investigator of the AMADILLO study and thereby supported LG in setting up this study's infrastructure in Kenya. LG trained and supervised data collectors, with guidance from PG. JAC and MW developed the statistical analysis plan. LG led the manuscript writing with heavy input from KW and AMH. All authors reviewed and edited drafts.

REFERENCES

1. MacQuarrie KLD. Unmet need for family planning among young women: Levels and trends. Rockville, MD: ICF International; 2014.
2. Chandra-Mouli V, McCarraher DR, Phillips SJ, Williamson NE, Hainsworth G. Contraception for adolescents in low and middle income countries: needs, barriers, and access. Reproductive Health. 2014;11(1):1.
3. High-Impact Practices in Family Planning (HIPs). Adolescent-friendly contraceptive services: mainstreaming adolescent-friendly elements into existing contraceptive services. . USAID; 2015.
4. (JSI) JSI. Private Shops and Pharmacies as Providers of Family Planning. 2016.
5. Corroon M, Kebede E, Spektor G, Speizer I. Key Role of Drug Shops and Pharmacies for Family Planning in Urban Nigeria and Kenya. Global Health: Science and Practice. 2016;4(4):594-609.
6. High-Impact Practices in Family Planning (HIP). Drug Shops and Pharmacies: Sources for family planning commodities and information. Washington DC: USAID; 2013.
7. Ostola J, Corroon M, Speizer I, Kebede E, Spektor G. Private sector distribution of family planning: The role of drug shops and pharmacies in urban Kenya and Nigeria. 2015.
8. Radovich E, Dennis ML, Wong KLM, Ali M, Lynch CA, Cleland J, et al. Who Meets the Contraceptive Needs of Young Women in Sub-Saharan Africa? Journal of Adolescent Health. 2018;62(3):273-80.
9. Gonsalves L, Hindin MJ. Pharmacy provision of sexual and reproductive health commodities to young people: a systematic literature review and synthesis of the evidence. Contraception. 2017;95(4):339-63.
10. World Health Organization. Family planning/Contraception Geneva, Switzerland: World Health Organization; 2018 [Available from: https://www.who.int/news-room/fact-sheets/detail/family-planning-contraception.

For peer review only - http://bmjopen.bmj.com/site/about/guidelines.xhtml

11. Ministry of Health/Kenya. National family planning guidelines for service providers: Updated to reflect the 2009 Medical Eligibility Criteria of the World Health Organization. Nairobi, Kenya: Division of Reproductive Health; 2010.
12. National Council for Law Reporting with the Authority of the Attorney-General/Kenya. Pharmacy and Poisons Act. Chapter 244. 1957 (revised 2012)(Cap 244).
13. Kwale County Government. Kwale County Integrated Development Plan (2018-2022): Continuing Kwale's Transformation Together 2018.
14. Kenya Ministry of Health. Kwale county: Health at a glance. 2015.
15. Gonsalves L, Hindin MJ, Bayer A, Carcamo CP, Gichangi P, Habib N, et al. Protocol of an open, three-arm, individually randomized trial assessing the effect of delivering sexual and reproductive health information to young people (aged 13–24) in Kenya and Peru via mobile phones: adolescent/youth reproductive mobile access and delivery initiative for love and life outcomes (ARMADILLO) study stage 2. Reproductive Health. 2018;15(1):126.
16. Gonsalves L, Njeri WW, Schroeder M, Mwaisaka J, Gichangi P. Research and Implementation Lessons Learned From a Youth-Targeted Digital Health Randomized Controlled Trial (the ARMADILLO Study). JMIR Mhealth Uhealth. 2019;7(8):e13005.
17. World Health Organization. Making health services adolescent friendly: developing national quality standards for adolescent friendly health services. Geneva, Switzerland: World Health Organization; 2012.
18. Riley C, Garfinkel D, Thanel K, Esch K, Workalemahu E, Anyanti J, et al. Getting to FP2020: Harnessing the private sector to increase modern contraceptive access and choice in Ethiopia, Nigeria, and DRC. PLOS ONE. 2018;13(2):e0192522.
19. The World Bank. Rural population (% of total population): World Bank staff estimates based on the United Nations Population Division's World Urbanization Prospects: 2018 Revision. New York, USA: World Bank Group; 2018 [Available from: https://data.worldbank.org/indicator/SP.RUR.TOTL.ZS.
20. Kenya National Bureau of Statistics, Ministry of Health/Kenya, National AIDS Control Council/Kenya, Kenya Medical Research Institute, Population NCf, Development/Kenya. Kenya Demographic and Health Survey 2014. Rockville, MD, USA; 2015.
21. Marston C, Renedo A, Nyaaba GN, Machiyama K, Tapsoba P, Cleland J. Improving the Measurement of Fertility Regulation Practices: Findings from Qualitative Research in Ghana. Int Perspect Sexual Reprodud Health. 2017;43(3):111-9.
22. International Consortium for Emergency Contraception. Counting what counts: Tracking access to emergency contraception - Kenya. 2013.
23. Cartwright A, Otai J, Maytan-Joneydi A, McGuire C, Sullivan E, Olumide A, et al. Access to family planning for youth: perspectives of young family planning leaders from 40 countries [version 1; peer review: 1 approved with reservations]. Gates Open Research. 2019;3(1513).
24. Peterson J, Brunie A, Diop I, Diop S, Stanback J, Chin-Quee D. Over the counter: The potential for easing pharmacy provision of family planning in urban Senegal [version 3; peer review: 1 approved, 2 approved with reservations]. Gates Open Research. 2019;2(29).
25. World Health Organization. Selected practice recommendations for contraceptive use: Third edition. Geneva, Switzerland; 2016.
26. Grossman D, Ellertson C, Abuabara K, Blanchard K, Rivas FT. Barriers to contraceptive use in product labeling and practice guidelines. Am J Public Health. 2006;96(5):791-9.
27. Leeman L. Medical Barriers to Effective Contraception. Obstetrics and Gynecology Clinics of North America. 2007;34(1):19-29.
28. Oliver M, Geniets A, Winters N, Rega I, Mbae SM. What do community health workers have to say about their work, and how can this inform improved programme design? A case study with CHWs within Kenya. Global Health Action. 2015;8(1):27168.

For peer review only - http://bmjopen.bmj.com/site/about/guidelines.xhtml

29. Gonsalves L, Wyss K, Gichangi P, Say L, Martin Hilber A. Regulating pharmacists as contraception providers: A qualitative study from Coastal Kenya on injectable contraception provision to youth. PLOS ONE. 2019;14(12):e0226133.
30. Oye-Adeniran BA, Adewole IF, Umoh AV, Oladokun A, Gbadegesin A, Odeyemi KA, et al. Sources of Contraceptive Commodities for Users in Nigeria. PLOS Medicine. 2005;2(11):e306.
31. World Health Organization. Family planning evidence brief: Reducing early and unintended pregnancies among adolescents. Geneva, Switzerland: World Health Organization; 2017.
32. Erulkar AS, Onoka CJ, Phiri, A;. What is youth-friendly? Adolescents' preferences for reproductive health services in Kenya and Zimbabwe. African Journal of Reproductive Health. 2005;9(3):51-8.

For peer review only

For peer review only - http://bmjopen.bmj.com/site/about/guidelines.xhtml

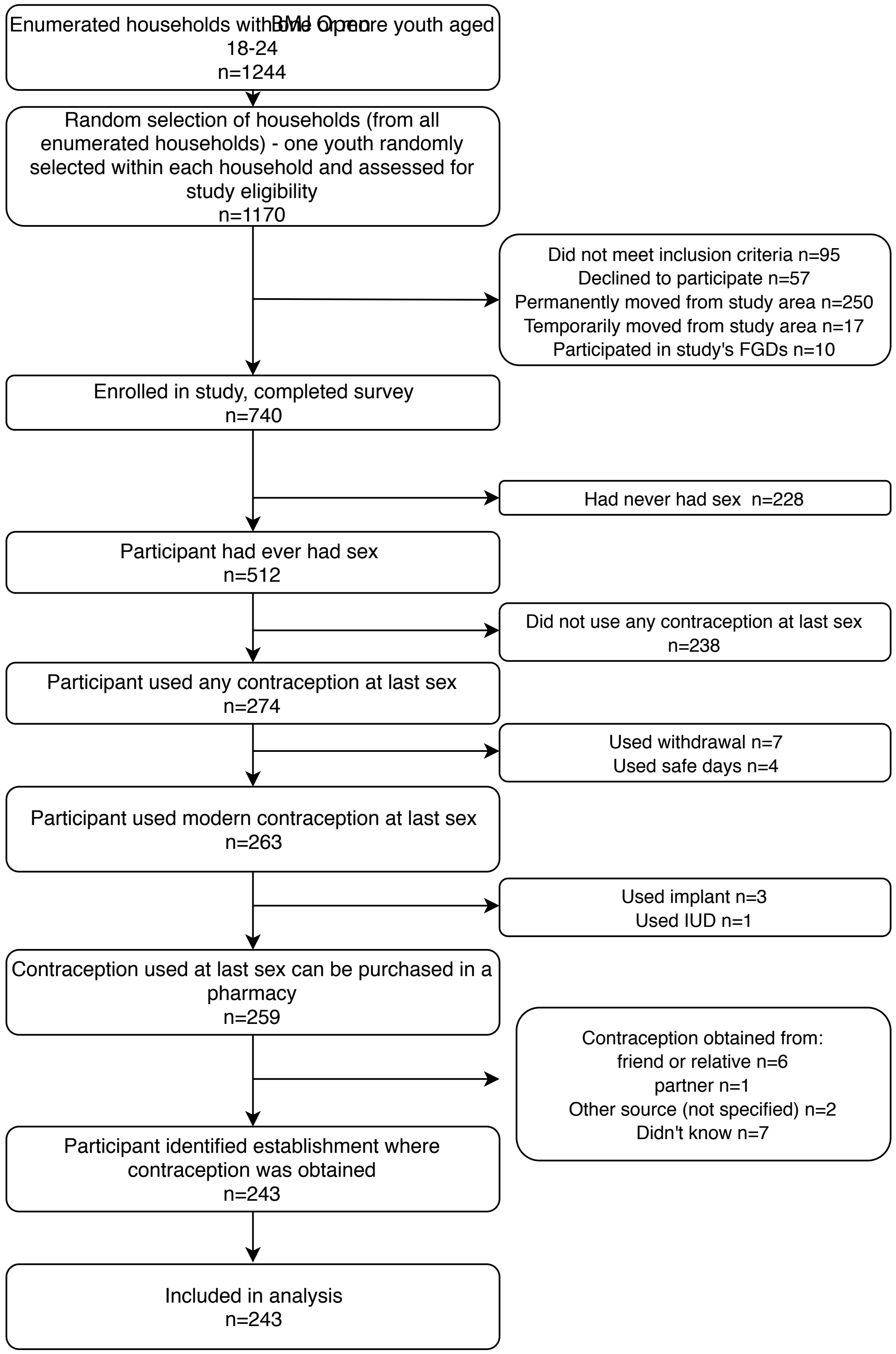
Enumerated households with one or more youth aged 18-24
n=1244
Random selection of households (from all enumerated households) - one youth randomly selected within each household and assessed for study eligibility
n=1170
Did not meet inclusion criteria n=95
Declined to participate n=57
Permanently moved from study area n=250
Temporarily moved from study area n=17
Participated in study's FGDs n=10
Enrolled in study, completed survey
n=740
Had never had sex n=228
Participant had ever had sex
n=512
Did not use any contraception at last sex
n=238
Participant used any contraception at last sex
n=274
Used withdrawal n=7
Used safe days n=4
Participant used modern contraception at last sex
n=263
Used implant n=3
Used IUD n=1
Contraception used at last sex can be purchased in a pharmacy
n=259
Contraception obtained from:
friend or relative n=6
partner n=1
Other source (not specified) n=2
Didn't know n=7
Participant identified establishment where contraception was obtained
n=243
Included in analysis
n=243

Supplementary Table 1 Selected characteristics of young people purchasing contraception at a pharmacy, shop, or any other source

	Pharmacy (N=153)	Shop (N=29)	Any other source (N=61)
Age			
18-19	27 (18%)	6 (21%)	10 (16%)
20+	126 (82%)	23 (79%)	51 (84%)
Sex			
Male	80 (52%)	24 (83%)	28 (46%)
Female	73 (48%)	5 (17%)	33 (54%)
Education			
Primary or below	60 (39%)	7 (24%)	29 (48%)
Secondary or above	93 (61%)	22 (76%)	32 (52%)
Relationship status			
Single	46 (30%)	10 (34%)	25 (41%)
Dating	86 (56%)	18 (62%)	11 (18%)
Cohabiting/Married	21 (14%)	1 (3%)	25 (41%)
Children			
No	139 (91%)	28 (97%)	37 (61%)
Yes	14 (9%)	1 (3%)	24 (39%)
Living situation			
Lives alone	30 (20%)	3 (10%)	6 (10%)
Lives with family (dependent)	113 (74%)	25 (86%)	32 (53%)
Lives with child or partner	10 (7%)	1 (3%)	23 (38%)
Contraception purchased			
Condom	120 (78%)	28 (97%)	33 (54%)
ECP	24 (16%)	1 (3%)	5 (8%)
Pills/Injections	9 (6%)	0 (0%)	23 (38%)

For peer review only

For peer review only - http://bmjopen.bmj.com/site/about/guidelines.xhtml

S1. Focus Group Discussion Guide

Today we're going to discuss what young people in this community think about contraceptives and where they go when they need it.

Warm-up

- Tell me what "contraceptive" means to you?
    - Tell me the kinds of contraceptives you've heard of

Myths and misinformation around contraception

Vignette: XXX [name determined by FGD participants] is 21 and her boyfriend YYY [name determined by FGD participants] is 23. They have been dating for awhile and are thinking about using contraceptives. However, there are things about contraceptives that they have heard from friends and family members which make them uncertain.

What are some of the things which they may have heard?

Ask participants to write down on sticky notes at least three things that XX and YY may have heard which would make them nervous. NoteTaker and Facilitator 3 will post these on the board, grouping together the similar ones. After they are all posted, moderator can ask:

- *[read out the reasons listed on the board]:* Are there any additional reasons XX and YY may feel uncertain that you can think of?
- *[also probe on certain reasons that are vague or broad]*

Where young people get contraceptives

- Tell me about all the places in _____ (study site town), where a young person can get contraceptives? *(Facilitator 3 writes out a list)*
- Describe all the different kinds of young people you could find in your community. *(keep this short)*

For each listed contraception source:

- Describe the kind of young person who would go to a _______ if he/she needed contraceptives? *(Draw stick figure under each source name, probe on and label with identifiers: gender, marital status, etc)*
  Facilitator 3 stops drawing after question above
- When would a young person choose to go to a _____ to get contraceptives?
    - [Note]: what kind of contraceptives are they getting
- Why would this young person choose to go to a ________ to get contraceptives over another source?

For peer review only

- [Probe] What are the best qualities about _______ as a resource for contraceptives?
- What might other young people *dislike* about _________ as a resource for contraceptives?

Qualities of ideal FP-dispensing in *non-service sources*

- What are the most important qualities a chemist or a shop needs to have for a young person to be comfortable obtaining contraceptives? [*Probe on person working vs the shop itself]*
- What could be some reasons why young people would not be comfortable going to chemists or shops?
    - What could be done to increase the comfort of young people who might not be comfortable going to chemists or shops?
- What **other** information and services would a young person needing contraceptives from a chemist or a shop also need?
    - [*be sure to probe on information AND services separately]*
- What could be done to make sure that young people can get the extra information and services (*that group mentioned in previous question*) that they need from chemists and shops without being uncomfortable and without sacrificing their privacy and speed (*or whatever is mentioned as an important quality).*

Close and thank people for their time

For peer review only

S2. In-Depth Interview guide for young contraception purchasers

Warm-up

- Tell me about what life is like for young people (people your age) in this community.
- What are some of the challenges that young people face?

As you told us earlier, you recently purchased family planning from a chemist shop nearby. I want to ask you about this experience

- Tell me about what your experience was like when purchasing FP from the chemist – how did it go, from beginning to end? *[looking for information on environment, interaction with chemists, how they were treated]*
    - How did you feel at each step?
    - What was the most difficult part of the experience?
    - What was the easiest part of the experience?
- Describe your interaction with the chemist attendant *[probe on: how were you treated? Did they give you advice]*
    - How did he/she react to your request
- Tell me about the information you were given by the chemist *[probe on: counselling, life advice, side effects, referrals other FP]*
- Tell me about what else was going on in the chemist shop while you were purchasing FP.
- How did you feel after you left the shop?
- Given the experience you've just described to me, how did that compare with what you *thought* would happen when you first walked in the chemist shop? *

Thinking about the time that you purchased family planning at the chemist, help me understand how you made that decision:

- What situation made you decide that you needed family planning? *[Probe on whether others were involved in this decision]*
- How did you decide what kind of family planning you wanted?
- Why did you decide to go to a chemist for family planning instead of other places?
    - Why did you select that particular chemist?
- How did this experience compare with other times you have bought family planning?

As a young person who has purchased FP from a chemist, I am interested to hear your ideas for how chemist shops could be improved for young people:

- Were there any parts of your experience that you liked?
- Based on your experience, was there anything you would've <u>liked</u> to happen that didn't?
    - *Probe (if necessary):* Based on these, is there any part of the experience that you want changed?
- If you worked at the chemist, what would you do to make young clients buying family planning feel comfortable?
- If you worked at the chemist, how could you improve the shop to be more friendly to young people needing family planning?

For peer review only

- What else do young people need to feel comfortable getting FP from chemists?

For peer review only

S3. Key Informant Interview guide (for a person working in a pharmacy)

Group 1 (Background – Personal)

- Tell me about yourself and how you came to work in the chemist?
    - Probe if they are from the area
    - What is their current title?
- Tell me about the roles and responsibilities of your job - describe a typical day of work
- What are the things that you enjoy about your job?
- What are the things you do not enjoy about your job?

Group 2 (Background – Shop)

- Tell me about who else works at this chemist
    - Probe: what are their roles and how are they different from yours?
- Describe how the chemist shop is organized?
- When are you busiest?
    - Probe: opening hours

Group 3 (Family planning)

- Tell me about the family planning in this chemist shop
    - Probe: what kinds are available, most popular, price
- Tell me about the kinds of people from the community who buy these family planning
    - Probe: Describe them, what they are looking for
- Why are chemist shops like yours important in providing family planning to the community?
    - Probe: How is this job different from health facilities that also have family planning?
- If a young person comes in asking for family planning, what are some of things you look at that help you decide what to recommend?
- What are the rules for dispensing family planning?
    - Probe: are there any exceptions to these rules?
- Describe the kinds training (either from your boss or from previous training) you received about family planning?

Group 4 (Feelings about selling family planning to young people)

- Think about the last time that young person (18-24) came to this chemist for some kind of family planning. Can you describe the interaction, from beginning to end?
    - Probe: what happens, what would they say, what would you say, what do you give them?
- How do young customers feel coming to ask for family planning (Probe: what do they say)
- What could chemist shops like this one do to improve the comfort of young people in the community who need family planning?
- When a young customer comes in asking for family planning, how do you feel?

For peer review only

- Are things you would like to tell them?
- If you had the power, what would you do to improve the confidence of chemists to provide family planning to young people?

For peer review only

S4. Tool 2 – Survey Instrument (Excerpt)

SOCIO-DEMOGRAPHIC INFORMATION. First we're going to talk about who you are.

1. What is your sex? Mark ONLY ONE.

 0 Male
 1 Female

2. What is your birthdate?

 Day |__|__| Month |__|__| Year |__|__|__|__|

3. What is the highest level of school you attended? Mark ONLY ONE.

 0 I've never gone to school
 1 Primary school
 2 Secondary school
 3 Post-secondary education **– GO TO 5**

4. What is the highest grade you completed at that level?

 |__|__| grade/form/level **– GO TO 6**

5. What type of post-secondary education did you attend/are you attending? Mark ONLY ONE.

 1 Technical post-secondary education
 2 University education

6. Who do you currently live with? Mark ALL possible options.

 0 I live alone
 1 Father/stepfather
 2 Mother/stepmother
 3 Siblings
 4 Grandparents
 5 Other relatives

For peer review only

6 Husband or wife **– *NOTE: Be sure to ask whether husband/wife or cohabiting partner.***
7 Cohabiting partner
8 In-laws
9 Children
10 Friends

7. What is your current relationship status?
   0 Single
   1 Friends with benefits
   2 Dating
   3 Cohabiting
   4 Engaged
   5 Married
   6 Other (specify)

8. How many children do you have?

   0 I have no children **– GO TO 10**

   |__|__| child/children

9. How old were you when you had your first child?

   |__|__| years old

…
[SURVEY CONTINUES]

ARMADILLO-RELATED BEHAVIORS. Now we're going to talk about sexual activity in order to gain a better understanding of some important life issues. Let me assure you again that your answers are completely confidential and will not be told to anyone.

29. How old were you when you had sexual intercourse for the very first time?

   |__|__| years old

   0 I have never had sexual intercourse **– GO TO 46**

30. Have you ever used any method to prevent pregnancy? By use, I mean that either you, yourself, have used the method or that a partner of yours used the method when having sex with you.

For peer review only

YES 1
NO...0
DON'T KNOW...8
REFUSED 9

31. When was the last time you had sex?

|__|__| days ago

|__|__| weeks ago

|__|__| months ago

|__|__| years ago

32. The last time you had sex, what was your relationship to this person with whom you had sexual intercourse?

1 Boyfriend not living with respondent
2 Girlfriend not living with respondent
3 Male cohabiting partner
4 Female cohabiting partner
5 Husband
6 Wife
7 Male casual acquaintance
8 Female casual acquaintance
9 Male sex worker
10 Female sex worker
11 Female client (respondent is male sex worker)
12 Male client (respondent is female sex worker)
13 Male relative
14 Female relative

33. The last time you had sex, did you or your partner use a contraceptive method?

0 No **– GO TO 45**
1 Yes

34. The last time you had sex, which contraceptive method did you or your partner use?

For peer review only

1 Male condom
2 Female condom
3 Birth control pill
4 Injectable
5 Implant
6 Intrauterine device (IUD)
7 Emergency contraception (the morning after pill)
8 Female sterilization
9 Male sterilization (vasectomy)
a. Withdrawal **– GO TO 44**
10 Rhythm method **– GO TO 44**

35. The last time you had sex, where did you or your partner obtain the contraceptive method you used?

1 A pharmacy or chemist
2 County Hospital
3 Health centres
4 A NGO
5 A private doctor or clinic
6 A shop/market
7 A community-based distributor
8 A peer educator
9 A traditional healer **– GO TO 44**
10 A friend or relative **– GO TO 44**
11 A partner **– GO TO 44**
12 Other **– GO TO 44**

36. When you obtained your [MOST RECENT CONTRACEPTIVE METHOD], were you told by the provider about side effects of problems you might have with a method to delay or avoid getting pregnant?
1 Yes
2 No

37. Were you told what to do if you experienced side effects or problems?
1 Yes
2 No

38. At that time, were you told by the family planning provider about methods of family planning other than [MOST RECENT CONTRACEPTIVE METHOD] that you could use?
1 Yes
2 No

39. During that visit did you obtain the method you wanted to delay or avoid getting pregnant?
1 Yes **– GO TO 42**

For peer review only

2 No

40. Why didn't you obtain the method you wanted?
    1 Method out of stock that day
    2 Method not available at all
    3 Provider not trained to provide the method
    4 Provider recommended a different method
    5 Not eligible for method
    6 Decided not to adopt a method
    7 Too costly
    8 Other

41. During that visit who made the final decision about what method you got?
    1 You alone
    2 Provider
    3 Partner
    4 You and provider
    5 You and partner
    6 Other

42. Would you return to this provider?
    1 Yes
    2 No

43. Would you refer your relative or friend to this provider/facility?
    1 Yes
    2 No

44. How important were each of the following characteristics to you in deciding which birth control method to use? *(read item, asking)* Would you say: not at all important, slightly important, quite important or extremely important to you in choosing a method?

	Not at all important	Slightly important	Quite important	Extremely important
A. It is very effective at preventing pregnancy				
B. It has a low cost.				

For peer review only

C. It is easy to use.				
D. It doesn't contain hormones.				
E. It is acceptable to my partner<br><br>F. It doesn't interrupt sex.				
G. It is effective at preventing HIV or STIs.				

For peer review only

Standards for Reporting Qualitative Research (SRQR)*

http://www.equator-network.org/reporting-guidelines/srqr/

Page/line no(s).

Title and abstract

**Title** - Concise description of the nature and topic of the study Identifying the study as qualitative or indicating the approach (e.g., ethnography, grounded theory) or data collection methods (e.g., interview, focus group) is recommended	Page 1/Line 1&2 (identified as mixed methods)
**Abstract** - Summary of key elements of the study using the abstract format of the intended publication; typically includes background, purpose, methods, results, and conclusions	Page 1-2

Introduction

**Problem formulation** - Description and significance of the problem/phenomenon studied; review of relevant theory and empirical work; problem statement	Page 3/Line 67-73, 87-89
**Purpose or research question** - Purpose of the study and specific objectives or questions	Page 3/Line 89-91

Methods

**Qualitative approach and research paradigm** - Qualitative approach (e.g., ethnography, grounded theory, case study, phenomenology, narrative research) and guiding theory if appropriate; identifying the research paradigm (e.g., postpositivist, constructivist/ interpretivist) is also recommended; rationale**	(see response to reviewers Page 3)
**Researcher characteristics and reflexivity** - Researchers' characteristics that may influence the research, including personal attributes, qualifications/experience, relationship with participants, assumptions, and/or presuppositions; potential or actual interaction between researchers' characteristics and the research questions, approach, methods, results, and/or transferability	Page 5/Line 166-175
**Context** - Setting/site and salient contextual factors; rationale**	Page 3/Line 93-98
**Sampling strategy** - How and why research participants, documents, or events were selected; criteria for deciding when no further sampling was necessary (e.g., sampling saturation); rationale**	Page 4/113-147
**Ethical issues pertaining to human subjects** - Documentation of approval by an appropriate ethics review board and participant consent, or explanation for lack thereof; other confidentiality and data security issues	Page 5/Line151-155
**Data collection methods** - Types of data collected; details of data collection procedures including (as appropriate) start and stop dates of data collection and analysis, iterative process, triangulation of sources/methods, and modification of procedures in response to evolving study findings; rationale**	Page5/Line141-151

For peer review only - http://bmjopen.bmj.com/site/about/guidelines.xhtml

**Data collection instruments and technologies** - Description of instruments (e.g., interview guides, questionnaires) and devices (e.g., audio recorders) used for data collection; if/how the instrument(s) changed over the course of the study	Page 5/Line 141-151 (and Supplementary materials)
**Units of study** - Number and relevant characteristics of participants, documents, or events included in the study; level of participation (could be reported in results)	Page 4/Table 1 Page 9/Line 243-254
**Data processing** - Methods for processing data prior to and during analysis, including transcription, data entry, data management and security, verification of data integrity, data coding, and anonymization/de-identification of excerpts	Page 6/Line192-194
**Data analysis** - Process by which inferences, themes, etc., were identified and developed, including the researchers involved in data analysis; usually references a specific paradigm or approach; rationale**	Page6/Line194-203
**Techniques to enhance trustworthiness** - Techniques to enhance trustworthiness and credibility of data analysis (e.g., member checking, audit trail, triangulation); rationale**	Page 6/Line193-194

Results/findings

**Synthesis and interpretation** - Main findings (e.g., interpretations, inferences, and themes); might include development of a theory or model, or integration with prior research or theory	Page 9/From Line 255 to end of section
**Links to empirical data** - Evidence (e.g., quotes, field notes, text excerpts, photographs) to substantiate analytic findings	Page 9/Table 4

Discussion

**Integration with prior work, implications, transferability, and contribution(s) to the field -** Short summary of main findings; explanation of how findings and conclusions connect to, support, elaborate on, or challenge conclusions of earlier scholarship; discussion of scope of application/generalizability; identification of unique contribution(s) to scholarship in a discipline or field	Page 12/Entire discussion section
**Limitations** - Trustworthiness and limitations of findings	Page12/Line316-328

Other

**Conflicts of interest** - Potential sources of influence or perceived influence on study conduct and conclusions; how these were managed	Page 14/Line 405
**Funding** - Sources of funding and other support; role of funders in data collection, interpretation, and reporting	Page 14/Line402-403

*The authors created the SRQR by searching the literature to identify guidelines, reporting standards, and critical appraisal criteria for qualitative research; reviewing the reference lists of retrieved sources; and contacting experts to gain feedback. The SRQR aims to improve the transparency of all aspects of qualitative research by providing clear standards for reporting qualitative research.

For peer review only

**The rationale should briefly discuss the justification for choosing that theory, approach, method, or technique rather than other options available, the assumptions and limitations implicit in those choices, and how those choices influence study conclusions and transferability. As appropriate, the rationale for several items might be discussed together.

Reference:

O'Brien BC, Harris IB, Beckman TJ, Reed DA, Cook DA. **Standards for reporting qualitative research: a synthesis of recommendations.** *Academic Medicine*, Vol. 89, No. 9 / Sept 2014 DOI: 10.1097/ACM.0000000000000388

For peer review only

For peer review only - http://bmjopen.bmj.com/site/about/guidelines.xhtml

STROBE Statement—checklist of items that should be included in reports of observational studies

	Item No.	Recommendation	Page No.	Relevant text from manuscript
**Title and abstract**	1	(*a*) Indicate the study's design with a commonly used term in the title or the abstract	1	Title
		(*b*) Provide in the abstract an informative and balanced summary of what was done and what was found	1-2	Abstract
**Introduction**				
Background/rationale	2	Explain the scientific background and rationale for the investigation being reported	3	Paragraph 1-4 of Introduction
Objectives	3	State specific objectives, including any prespecified hypotheses	3	"Therefore, this mixed methods study sought to answer two questions…"
**Methods**				
Study design	4	Present key elements of study design early in the paper	3-4	"This analysis was part of a broader, mixed-methods study describing how young people (aged 18-24) in Kwale County obtain contraception from pharmacies." + Table 1
Setting	5	Describe the setting, locations, and relevant dates, including periods of recruitment, exposure, follow-up, and data collection	4-5	Table 1 + Methods text
Participants	6	(*a*) *Cohort study*—Give the eligibility criteria, and the sources and methods of selection of participants. Describe methods of follow-up<br>*Case-control study*—Give the eligibility criteria, and the sources and methods of case ascertainment and control selection. Give the rationale for the choice of cases and controls<br>*Cross-sectional study*—Give the eligibility criteria, and the sources and methods of selection of participants	4 (cross-sectional)	Table 1, "In October 2017, data collectors enumerated all households…"
		(*b*) *Cohort study*—For matched studies, give matching criteria and number of exposed and unexposed<br>*Case-control study*—For matched studies, give matching criteria and the number of controls per case		N/A

For peer review only - http://bmjopen.bmj.com/site/about/guidelines.xhtml

Variables	7	Clearly define all outcomes, exposures, predictors, potential confounders, and effect modifiers. Give diagnostic criteria, if applicable	6	Analysis section
Data sources/ measurement	8*	For each variable of interest, give sources of data and details of methods of assessment (measurement). Describe comparability of assessment methods if there is more than one group	N/A	Not included beyond primary outcome (to leave space to discuss qual methods)
Bias	9	Describe any efforts to address potential sources of bias	5	"Data collectors entered responses save…"
Study size	10	Explain how the study size was arrived at	4	"The sample size was calculated based on the ARMADILLO trial's primary outcome…"
Quantitative variables	11	Explain how quantitative variables were handled in the analyses. If applicable, describe which groupings were chosen and why	6	Analysis section – for primary outcome
Statistical methods	12	(*a*) Describe all statistical methods, including those used to control for confounding	6	Analysis section
		(*b*) Describe any methods used to examine subgroups and interactions	N/A	Based on primary outcome, no subgroups were examined
		(*c*) Explain how missing data were addressed	N/A	Not included (to leave space to discuss qual methods)
		(*d*) *Cohort study*—If applicable, explain how loss to follow-up was addressed<br>*Case-control study*—If applicable, explain how matching of cases and controls was addressed<br>*Cross-sectional study*—If applicable, describe analytical methods taking account of sampling strategy	N/A	N/A
		(*e*) Describe any sensitivity analyses	N/A	N/A
**Results**				
Participants	13*	(a) Report numbers of individuals at each stage of study—eg numbers potentially eligible, examined for eligibility, confirmed eligible, included in the study, completing follow-up, and analysed	N/A	Not included (to leave space to discuss qual methods – reference describing this in detail is included [15] on page 4)
		(b) Give reasons for non-participation at each stage	N/A	Not applicable (cross-section)

For peer review only - http://bmjopen.bmj.com/site/about/guidelines.xhtml

		(c) Consider use of a flow diagram	N/A	Not included (to leave space to discuss qual methods – reference to open source article with this information is included [15] on page 4)
Descriptive data	14*	(a) Give characteristics of study participants (eg demographic, clinical, social) and information on exposures and potential confounders	6-7	Table 2
		(b) Indicate number of participants with missing data for each variable of interest	N/A	None for primary outcome
		(c) *Cohort study*—Summarise follow-up time (eg, average and total amount)		
Outcome data	15*	*Cohort study*—Report numbers of outcome events or summary measures over time		
		Case-control study—Report numbers in each exposure category, or summary measures of exposure		
		Cross-sectional study—Report numbers of outcome events or summary measures	8	Table 3
Main results	16	(*a*) Give unadjusted estimates and, if applicable, confounder-adjusted estimates and their precision (eg, 95% confidence interval). Make clear which confounders were adjusted for and why they were included	8-9	Table 3
		(*b*) Report category boundaries when continuous variables were categorized	8-9	Table 3
		(*c*) If relevant, consider translating estimates of relative risk into absolute risk for a meaningful time period	N/A	N/A
Other analyses	17	Report other analyses done—eg analyses of subgroups and interactions, and sensitivity analyses	N/A	N/A
**Discussion**				
Key results	18	Summarise key results with reference to study objectives	11-12	Discussion paragraph 1
Limitations	19	Discuss limitations of the study, taking into account sources of potential bias or imprecision. Discuss both direction and magnitude of any potential bias	11-12	Discussion paragraph 2
Interpretation	20	Give a cautious overall interpretation of results considering objectives, limitations, multiplicity of analyses, results from similar studies, and other relevant evidence	12	Discussion section paragraph 3-4
Generalisability	21	Discuss the generalisability (external validity) of the study results	12	Discussion section paragraph 3
**Other information**				
Funding	22	Give the source of funding and the role of the funders for the present study and, if applicable, for the original study on which the present article is based	14	Funding statement

For peer review only

*Give information separately for cases and controls in case-control studies and, if applicable, for exposed and unexposed groups in cohort and cross-sectional studies.

Note: An Explanation and Elaboration article discusses each checklist item and gives methodological background and published examples of transparent reporting. The STROBE checklist is best used in conjunction with this article (freely available on the Web sites of PLoS Medicine at http://www.plosmedicine.org/, Annals of Internal Medicine at http://www.annals.org/, and Epidemiology at http://www.epidem.com/). Information on the STROBE Initiative is available at www.strobe-statement.org.

For peer review only

BMJ Open

Mixed-methods study on pharmacies as contraception providers to Kenyan young people: who uses them and why?

Journal:	*BMJ Open*
Manuscript ID	bmjopen-2019-034769.R2
Article Type:	Original research
Date Submitted by the Author:	10-Mar-2020
Complete List of Authors:	Gonsalves, Lianne; World Health Organization, Department of Sexual and Reproductive Health and Research including UNDP/UNFPA/UNICEF/WHO/World Bank Special Programme of Research, Development and Research Training in Human Reproduction (HRP); Swiss Tropical and Public Health Institute<br>Wyss, Kaspar; Swiss Tropical and Public Health Institute; University of Basel<br>Cresswell , Jenny; World Health Organization, Department of Sexual and Reproductive Health and Research including UNDP/UNFPA/UNICEF/WHO/World Bank Special Programme of Research, Development and Research Training in Human Reproduction (HRP)<br>Waithaka, Michael; International Centre for Reproductive Health Kenya<br>Gichangi, Peter; International Centre for Reproductive Health Kenya; University of Nairobi, Department of Human Anatomy<br>Martin Hilber, Adriane; Swiss Tropical and Public Health Institute; University of Basel
**Primary Subject Heading**:	Sexual health
Secondary Subject Heading:	Respiratory medicine, Public health
Keywords:	REPRODUCTIVE MEDICINE, Community child health < PAEDIATRICS, PUBLIC HEALTH, QUALITATIVE RESEARCH

SCHOLARONE™
Manuscripts

I, the Submitting Author has the right to grant and does grant on behalf of all authors of the Work (as defined in the below author licence), an exclusive licence and/or a non-exclusive licence for contributions from authors who are: i) UK Crown employees; ii) where BMJ has agreed a CC-BY licence shall apply, and/or iii) in accordance with the terms applicable for US Federal Government officers or employees acting as part of their official duties; on a worldwide, perpetual, irrevocable, royalty-free basis to BMJ Publishing Group Ltd ("BMJ") its licensees and where the relevant Journal is co-owned by BMJ to the co-owners of the Journal, to publish the Work in this journal and any other BMJ products and to exploit all rights, as set out in our licence.

The Submitting Author accepts and understands that any supply made under these terms is made by BMJ to the Submitting Author unless you are acting as an employee on behalf of your employer or a postgraduate student of an affiliated institution which is paying any applicable article publishing charge ("APC") for Open Access articles. Where the Submitting Author wishes to make the Work available on an Open Access basis (and intends to pay the relevant APC), the terms of reuse of such Open Access shall be governed by a Creative Commons licence – details of these licences and which Creative Commons licence will apply to this Work are set out in our licence referred to above.

Other than as permitted in any relevant BMJ Author's Self Archiving Policies, I confirm this Work has not been accepted for publication elsewhere, is not being considered for publication elsewhere and does not duplicate material already published. I confirm all authors consent to publication of this Work and authorise the granting of this licence.

For peer review only

Mixed-methods study on pharmacies as contraception providers to Kenyan young people: who uses them and why?

Lianne Gonsalves[123], Kaspar Wyss[23], Jenny A Cresswell[1], Michael Waithaka[4], Peter Gichangi[456], Adriane Martin Hilber[23]

[1]Department of Reproductive Health and Research including UNDP/UNFPA/UNICEF/WHO/World Bank Special Programme of Research, Development and Research Training in Human Reproduction (HRP), World Health Organization, Geneva, Switzerland
[2]Swiss Tropical and Public Health Institute (Swiss TPH), Basel, Switzerland
[3]University of Basel, Basel, Switzerland
[4]International Centre for Reproductive Health Kenya, Mombasa, Kenya
[5]Department of Human Anatomy, University of Nairobi, Nairobi, Kenya
[6]Ghent University, Ghent, Belgium

Corresponding author information:

Lianne Gonsalves
Address: World Health Organization, Avenue Appia 20, 1211 Geneva, Switzerland
Telephone: +41 22 791 29 70
Email address: gonsalvesl@who.int

Word count, excluding title page, abstract, references, figures and tables

3961

Abstract: 272 words

Objectives

This study sought to answer two questions: 1) what are the characteristics of young Kenyans aged 18-24 who use contraception obtained at pharmacies, and 2) why are pharmacies appealing sources of contraception?

Design and Setting

This was a mixed-methods study in one peri-urban part of Kwale County, Kenya. Methods included: cross-sectional survey (N=740); six focus group discussions; 18 in-depth interviews; and 25 key informant interviews. Quantitative data analysis identified factors pushing young people to pharmacies for modern contraception versus other sources. Qualitative data analysis identified reasons pharmacies were perceived to be appealing to young clients.

Participants

Participants were: 1) young people aged 18-24 from the study area, including a subset who had recently purchased contraception from a pharmacy; or 2) pharmacy personnel and pharmacy stakeholders.

For peer review only - http://bmjopen.bmj.com/site/about/guidelines.xhtml

Results

Among surveyed participants who had ever had sexual intercourse and had used modern contraception at last sexual intercourse, 59% obtained it from a pharmacy. In multivariable analysis, participants who used a condom or emergency contraception as well as those living alone were significantly more likely to get contraception from pharmacies. Pharmacies were valued for their: convenience; privacy; non-judgmental and personable staff; service speed; and predictable, affordable prices.

Conclusions

Our findings indicate a high percentage of young people in Coastal Kenya use pharmacies for contraception. Our inclusion of emergency contraception users partially explains this. Pharmacies were perceived to be everything that health facilities are not: fast, private and non-limiting. Policymakers should recognize the role of pharmacies as contraception providers and look for opportunities to link pharmacies to the public health system. This would create a network of accessible and appealing contraception services for young people.

Strengths and limitations of this study

- Participants were asked to specify where they or their partner had obtained the contraception used at last sexual intercourse. This is a standard question for studies looking to establish contraception prevalence. Our not further ascertaining who specifically obtained the contraception affected our ability to distinguish differences in preferences of young men versus young women.
- One participant group (young people who had recently purchased contraception from a pharmacy) was recruited from five purposively selected pharmacies: this may limit the generalizability of the findings.
- This study is strengthened by its mixed methods design and inclusion of both pharmacy personnel and young people to triangulate research findings on a sensitive subject.

For peer review only

For peer review only - http://bmjopen.bmj.com/site/about/guidelines.xhtml

INTRODUCTION

Young people need access to contraception. However, around the world, and in low- and middle-income countries in particular, public sector contraceptive services are not meeting this need. Data from 61 low- and middle-income countries estimated that 33 million young women aged 15-24 had an unmet need for family planning.[1] Adolescents (ages 10-19 years) and youth (15-24 years) are often reluctant to access contraception at public health facilities where they may encounter a lack of privacy, biased providers, and limited contraceptive options, in addition to broader financial, legal, social, and cultural barriers. [2, 3]

Other parts of the health system may be able to step in to help fill this gap. In Kenya (where this study took place) and in the region, private pharmacies have become a source of modern contraception for young people. [4-7] Additional research has indicated that when contraception is introduced in pharmacies, access improves for young people.[8, 9] An analysis of 33 sub-Saharan African countries found that commercial drug sellers, including pharmacies, were the source of the most recent contraceptive method for nearly one in five young people between 15-24 years of age. [8] When also factoring in other informal and non-medical providers, including shops, these sources together serviced nearly half of women age 15-19. [8]

Kenya's National Family Planning Guidelines allow for the provision of several kinds of modern methods [10] of contraception to be dispensed by pharmacists or pharmaceutical technologists [11](colloquially referred to as 'chemists'). These include barrier methods like male and female condoms, as well as short-acting methods including emergency contraception (ECP), oral contraceptive pills, and injectable contraception. Injectables can be dispensed but not administered. These permissions mean that outside of health facilities, private retail pharmacies have the largest selection of modern methods available (shopkeepers can also sell condoms, per the guidelines). Private retail pharmacies must be opened by and should always operate under the supervision of either a pharmacist or pharmaceutical technologist.[12]

Despite their demonstrable popularity among young people, there is little data on the individual-level circumstances or characteristics of young people that would drive them to pharmacies for contraception. Therefore, we conducted a mixed-methods study describing how young people (aged 18-24) in Kwale County obtain contraception from pharmacies. Kwale County is one of six counties in Kenya's former Coast region. Young people between the ages of 15-24 were projected to make up 19% of the county's population by 2018.[13] In 2014, contraception prevalence in the county was 38%, lower than the national average of 53%. [14]

In this analysis, we sought to answer two questions: 1) what are the characteristics of young people who use contraception obtained at pharmacies, and 2) why are pharmacies appealing sources of contraception to young people?

METHODS

The study took place in the peri-urban areas of Kwale Town and Ukunda, as well as the stretch of highway connecting the two towns. Data collection took place between October 2017 and March 2018. We used several methods (captured in Table 1) to understand the experiences of pharmacy personnel and young people themselves. This study was partly-nested in the ARMADILLO randomized controlled trial (RCT)[15], which assessed the effect of an unrelated digital health intervention on sexual and reproductive health-related outcomes for young people aged 18-24.

Table 1 Study Methods

For peer review only - http://bmjopen.bmj.com/site/about/guidelines.xhtml

Method	N	Eligibility criteria	Relevant topics addressed
**Cross-sectional survey***	**740**	• Age 18-24<br>• Literate<br>• Have their own mobile phone (with them at time of recruitment) and report regular use<br>• Report current use of text messaging	• Contraception used at last sexual intercourse and source<br>• Demographic and behavioral characteristics
**Focus group discussions***	**6 (58 participants)**	• Age 18-24<br>• Community members	• Sources of contraception for young people<br>• Characteristics of young people who use each source
**In-depth interviews**	**18**	• Age 18-24<br>• Recently purchased contraception at pharmacy	• Reasons for having purchased contraception from pharmacy<br>• What was valued (and not valued) about experience
**Key-informant interviews**	**19 (pharmacy personnel)<br>6 (stakeholders)**	• Age 18+<br>• Pharmacy personnel (any role) OR<br>• Pharmacy-related stakeholder (Ministry of Health; regulatory agency; professional association; non-governmental organization)	• Characteristics of young people who purchase contraception<br>• What clients appreciate about experience

107 * Methods which were nested in the broader ARMADILLO Study, a digital health intervention RCT. Inclusion/exclusion criteria for these nested methods were determined by ARMADILLO's objectives.

To capture the perspectives of young people, a cross-sectional survey of young people age 18-24 captured demographic information and contraceptive use patterns, including source of last contraception (these questions were one section of a broader survey conducted as part of the baseline assessment for the ARMADILLO trial). The sample size was calculated based on the ARMADILLO trial's primary outcome – the full protocol for the trial has been previously published[15], along with details of participants recruited.[16]

To identify participants, we obtained a map of the study area from the Kenya National Bureau of Statistics. The KNBS divides the country into so-called 'enumeration areas' (EAs) in preparation for the country's 2019 census. EAs consist of blocks of households. Each EA had approximately 100 households. In October 2017, data collectors enumerated all age-eligible young people in every household using a random selection of 21 EAs in the study area. From this list of age-eligible youth, a random selection of households and random selection of one youth per household was generated. Data collectors visited the selected households to recruit participants (who met eligibility criteria captured in Table 1) starting in February 2018.

Additionally, six Focus Group Discussions were conducted with young people age 18-24, purposively recruited from the community by data collectors. Finally, we conducted in-depth interviews with 18

For peer review only - http://bmjopen.bmj.com/site/about/guidelines.xhtml

young people aged 18-24 who had recently purchased contraception from pharmacies. We purposively recruited these young participants in one of two ways. First, we stationed a young data collector outside of well-trafficked pharmacies over three evenings, who recruited young people purchasing contraception. Second, several pharmacists in the study area were provided with leaflets with study information and requested to provide these to young contraception purchasers at the end of a transaction.

To capture the perspectives of pharmacy personnel, data collectors mapped all private, retail pharmacies in the study area using a digital form with an embedded geolocator. A random subset of pharmacies was generated using the random number generator in Excel. Pharmacies were well-distributed across the study area. In each selected pharmacy, data collectors were instructed to approach the first person behind the counter, regardless of rank or level of training, explain the study and ask if they would be interested in participating. Nineteen interviews in total were conducted. An additional six key-informant interviews were conducted with stakeholders from the regulatory Pharmacy and Poisons Board, Ministry of Health, professional associations, and non-governmental organizations. These were conducted in the individuals' offices in either Ukunda, Mombasa, or Nairobi. Stakeholder participants were contacted first by phone or email, the studied explained, and a convenient time for an in-person visit set.

Data collection and management

We obtained informed consent from all participants prior to participation. All data were collected in English, Swahili, or a mix of the two, depending on participants' preference. Quantitative surveys were close-ended and administered using webforms on a tablet. Data collectors entered responses save for the questions related to participants' sexual and contraceptive use history; here, to reduce potential discomfort and response bias, participants entered their own responses. Interviews and FGDs used semi-structured guides: FGD (S1), in-depth interview (S2), and key-informant interview (S3) guides are provided as supplementary material, as are relevant survey components (S4). Qualitative data collection was informed by ground theory [17], allowing us to adopt an iterative approach, with question guides modified based on emerging themes. Qualitative data collection ceased upon reaching saturation. All qualitative methods used audio-recording (with participant permission). All study activities were conducted in a private location. Data collectors, speaking both English and Swahili, were recruited from the study area and specifically trained for this study.

This study received ethics approval from the Ethikkommission Nordwest- und Zentralschweiz (EKNZ) (Req-2017-00389) in Basel, Switzerland, as well as the University of Nairobi/Kenyatta National Hospital in Nairobi, Kenya (P274/05/2017). The ARMADILLO RCT also received ethics approval from the World Health Organization (Protocol WHO A65892) and is registered with the ISRCTN Registry (ISRCTN85156148).

Patient and public involvement

Our population (young people) were directly involved in parts of the study's design and implementation. Our survey data collection team consisted of young people recruited from the study area (Kwale County). Qualitative method data collectors were also young people recruited from both Kwale and Mombasa Counties. We relied on their insight and lived experience to determine how young people would feel most comfortable being recruited. We jointly designed our recruitment and consenting procedures. A dissemination meeting involving local, county, and national stakeholders (including some pharmacy stakeholder participants) took place in June, 2019. Several young data collectors were invited to attend and they provided commentary on the findings.

For peer review only - http://bmjopen.bmj.com/site/about/guidelines.xhtml

Researcher characteristics and reflexivity

Data collectors were young people (nearly even numbers of men and women – 24 in total) recruited from Kwale and Mombasa counties. Kwale County data collectors were familiar with the study area and recognized within their communities, which facilitated enumerating pharmacies, recruiting youth participants, and getting consent to interview pharmacy personnel. They were also less educated and less experienced than data collectors from Mombasa County. This, at times, resulted in a subordinate dynamic with some pharmacy personnel participants who were university-educated. The first author conducted all interviews with pharmacy stakeholders. She is from the United States (from a racial minority group different from the study population) and presented as an outsider (someone not from Kenya) to interviewees. Her position (leading the study and professional affiliations) resulted in respondents treating her collegially and being open to participate.

Analysis

Quantitative data were analyzed in Stata Version 14. The subject of the analyses (as described in Figure 1) were survey participants who reported using one of four contraception commodities available in pharmacies (either male or female condom, ECP, daily contraceptive pills, or injectable contraception) at last sexual intercourse *and* who reported their source. Sexual intercourse was presumed to be penetrative vaginal sex. Excluded were those participants who had not used contraception at last sexual intercourse, who had not used a contraceptive commodity (withdrawal method, calendar days), who could not remember where they had obtained their method and/or who had obtained it from a partner or friend. We developed a dichotomous 'source of family planning' outcome, distinguishing between 'pharmacy' and 'any other source'. The latter included any public or private health facility, community-based distributors, non-governmental organizations, shops, schools, supermarkets. Following descriptive statistics, bivariate log binomial regressions assessed the association between the outcome and each behavioral/sociodemographic variable of interest. Any analysis showing a $p<.2$ moved the variable into a multivariable Poisson regression model with robust 95% CIs.

Figure 1 Flow Diagram of Study Participants

All qualitative data were analyzed using the Framework Method. [18] Data were first transcribed verbatim and then translated (if necessary) into English. For a sub-section of Swahili-language interviews, English-language transcripts were compared against the original Swahili-language interview audio file by another member of the research team to ensure consistency. Qualitative analysis for the broader study was guided by the five, *WHO-defined dimensions of quality health services to adolescents*: equity, accessibility, acceptability, appropriateness, and effectiveness. [19] All transcripts were read once to improve familiarity with the data. Then, qualitative analysis was conducted in Atlas.ti Version 8, with deductive and then inductive coding of a subset of transcripts to develop and refine a coding framework. Deductive coding was informed by the 'accessibility' and 'acceptability' dimensions and broadly captured any reference to pharmacies being 'appealing'. Inductive coding of these data then identified specific reasons for appeal, subsequently grouping these into broad categories related to pharmacy outlet, personnel, and service appeal. These broad categories and individual reasons structure the presentation of the qualitative results.

For peer review only - http://bmjopen.bmj.com/site/about/guidelines.xhtml

RESULTS

Survey sample characteristics

A total of 1170 youth were approached for participation, of which 740 (63%) consented to participate and completed the survey. Reasons for non-participation are captured in Figure 1. As seen in Table 2, of the 740 young people aged 18-24 who participated in the cross-sectional survey, 512 (69%) had ever had sexual intercourse. Male condoms were the most popular form of contraception purchased, used by 190 of the 274 (69%) participants who used contraception at last sexual intercourse. Of the participants indicating that they used a modern contraceptive at last sexual intercourse (N=263), 154 (59%, data not shown) had obtained it from a private, retail pharmacy (hereafter, 'pharmacy').

Table 2 Baseline characteristics

	**All surveyed participants (N=740)**		
	Female	Male	Total
Ever had sexual intercourse	231/347	281/393	512/740 (69%)
Used any contraception at last sexual intercourse	126/231 (55%)	148/281 (53%)	274/512 (54%)
Used a modern contraceptive at last sexual intercourse	118/231 (51%)	145/281 (52%)	263/512(51%)
Used pharmacy-available contraception*	116/231 (50%)	143/281 (51%)	259/512 (51%)
Where contraception was obtained	(N=116)	(N=143)	(N=259)
Pharmacy	63%	56%	59%
Shop	5%	17%	11%
Public dispensary or health centre	13%	7%	10%
Hospital	11%	6%	8%
NGO, private doctor	3%	4%	4%
Community-based distributor, school, supermarket	1%	2%	2%
*Other person***	1%	4%	*3%*
*Other source (not specified)/Don't know ***	3%	3%	*3%*
	**Included participants using pharmacy-available contraception (N=243)**		
	Female (N=111)	Male (N=132)	Total (N=243)
Age			
18-19	17%	18%	18%
20-24	83%	82%	82%
Education (highest level attended)			
Primary or below	54%	27%	40%
Secondary	38%	55%	47%
Post-secondary	8%	18%	14%
Relationship status			
Single	23%	42%	33%
Friends with benefits	3%	8%	5%
Dating	42%	42%	42%
Cohabiting	3%	1%	2%
Engaged	9%	5%	7%
Married	20%	3%	11%
Any children			

For peer review only - http://bmjopen.bmj.com/site/about/guidelines.xhtml

No	74%	92%	84%
Yes	26%	8%	16%
Living situation			
Lives alone	8%	23%	16%
Lives with family (dependent)	66%	73%	70%
Lives with child or partner	26%	4%	14%
Contraception used***			
Male condom	56%	86%	72%
Female condom	4%	2%	2%
ECP	20%	6%	12%
Daily contraceptive pills	5%	2%	3%
Injection	16%	5%	10%

*these included male or female condom, emergency contraception (ECP), daily contraceptive pills, and injectable contraception

**these were excluded from analysis

***Participants could enter one contraceptive method

Of the 243 participants who were included in bivariate and multivariable analyses, 54% were male, 61% had attended secondary school or higher, and 70% were dependents (living with parents, grandparents, or other older family members). A higher proportion of female participants than male participants were cohabiting, engaged, or married and had at least one child. Male participants had attended higher levels of schooling than female participants. Supplementary Table 1 presents selected characteristics of the 243 participants disaggregated by whether they obtained contraception at a pharmacy, shop, or any other source: most shop users were male and purchased condoms.

Who accesses contraception from pharmacies?

Bivariate analyses (Table 3) indicated there was no evidence of an association between either age, sex, or education and a young person's contraception being from a pharmacy. There was an association between pharmacy-purchased contraception and a participant's relationship status, and whether they had children. The greatest predictors of whether a young person had visited a pharmacy were the type of contraception they purchased and with whom they lived. Following multivariate analysis (Table 3), there remained strong evidence of an association between pharmacy purchase of contraception and a young person's relationship status, living situation, as well as the type of contraception they used. Young people living alone were almost twice as likely to have sourced contraception from a pharmacy as those living with a child or partner (Adjusted PR 1.96, 95% CI [1.07-3.59]). Use of ECP remained the greatest predictor of a pharmacy purchase (Adjusted PR 2.27 as compared with pill/injection use 95% CI [1.21-4.27]).

Table 3 Bivariate and multivariable analysis to identify personal characteristics that may be associated with a young person obtaining contraception from a pharmacy (vs any other source)

	Purchased contraception from pharmacy	Unadjusted Prevalence Ratio (PR) [95% CI]	p-value*	Adjusted Prevalence Ratio (PR) [95% CI]	p-value
All	153/243 (63%)				
Age					
18-19	27/43 (63%)	Ref			

For peer review only - http://bmjopen.bmj.com/site/about/guidelines.xhtml

20-24	126/200 (63%)	1.00 [0.78-1.29]	0.979		
Sex					
Male	80/132 (61%)	Ref			
Female	73/111 (66%)	1.09 [0.90-1.32]	0.405		
Education					
Primary or below	60/96 (63%)	Ref			
Secondary or above	93/147 (63%)	1.01 [0.83-1.23]	0.904		
Relationship status					
Single	46/81 (57%)	0.76 [0.61-0.94]	0.0013	0.75 [0.61-0.93]	0.0284
Dating/'Friends with benefits'	86/115 (75%)	Ref		Ref	
Married/Engaged/Cohabiting	21/47 (45%)	0.60 [0.43-0.84]		0.95 [0.67-1.35]	
Children					
No	139/204 (68%)	1.89 [1.24-2.92]	0.003	1.25 [0.80-1.97]	0.318
Yes	14/39 (36%)	Ref		Ref	
Living situation					
Lives alone	30/39 (77%)	2.62 [1.51-4.53]	0.0024	1.96 [1.07-3.59]	0.0119
Lives with family (dependent)	113/170 (66%)	2.26 [1.33-3.85]		1.53 [0.84-2.82]	
Lives with child or partner	10/34 (29%)	Ref		Ref	
Contraception used					
Condom (m/f)	120/181 (66%)	2.36 [1.34-4.14]	0.0014	1.87 [1.02- 3.43]	0.0224
ECP	24/30 (80%)	2.84 [1.59-5.09]		2.27 [1.21-4.27]	
Pills/Injection	9/32 (28%)	Ref		Ref	

*any variable with p-values <.2 in bivariate analysis were included in the multivariable analysis

Qualitative methods participant characteristics

Three FGDs were held with young men, and three with young women – each FGD had approximately ten participants. Of the 18 in-depth interview participants, ten were young women and eight were young men. Female IDI participants had most recently purchased emergency contraception (n=7), injection (n=2), and condom (n=1). Male IDI participants had most recently purchased condom (n=6), and emergency contraception (n=2).

Of the 19 key informant participants, 10 interviewed pharmacy personnel were women, 9 were men. Participants were not probed in detail on their formal training (and therefore whether they should be operating in their current role). That said, we could ascertain that 13 of the participants had an appropriate amount of training for their reported tasks, and four did not (the final two were unclear). Self-reported education ranged from having some secondary education to full training as a pharmacist or pharmaceutical technologist. One participant was a nurse. Stakeholders demographics are not described to ensure they remain unidentifiable.

Why are pharmacies appealing?

Participants indicated that it was a combination of the pharmacy *outlet,* the pharmacy *personnel* themselves, and the *services* provided by the pharmacy which together made these establishments the preferred source of contraception for many young people (Table 4).

Table 4 Reasons why pharmacies are appealing (selected excerpts from qualitative data)

For peer review only - http://bmjopen.bmj.com/site/about/guidelines.xhtml

**Outlet appeal**	*The physical pharmacy environment and its operation*
Convenience (locations and hours)	"The chemist is near and whenever you want it [family planning] you can access it, anytime." Female pharmacy purchaser: injection<br><br>"The good thing with chemist is that they are many of them…when you missed a certain contraceptive at a certain chemist you can go to the next chemist because they are several of them, not like the hospital" – Female community member (FGD)<br><br>"Yes, majority of them [young people] don't live near health centres. Second, health centres are usually busy. And it's not every day they [can be] attended to: there are specific days they have clinics… [The client] won't be able to make it there…even if the treatment was free. But there is a chemist - [they] can go for similar services." – Pharmacist
Anonymity	"At the chemist there are not many people. I may go to Diani dispensary [a local public health facility], and there is someone who knows me and I go for family planning. I saw it would be better to go the chemist because I know that will be my secret and the attendant." Female pharmacy purchaser: emergency contraception<br><br>"When you go to the facility, when you go to the FP room, everyone knows that you've gone to get FP. For young people [especially] because no one will want to see me - I'm 18, I'm 16 and I'm already using family planning. I'm not supposed to be sexually active. The kind of population that is in those FP areas, around those FP areas it's your mothers who are either breastfeeding, or they're pregnant and have gone for ANC." – Ministry of Health official, County level
**Personnel appeal**	*The person behind the counter*
Interpersonal relationship	"the chemist is just within the neighborhood and I know the guy he is my friend outside job so it wasn't stressful for me in fact it was really fast and easy." – Male pharmacy purchaser: ECP and condoms<br><br>"The person in charge is my friend, I can go to him with my problems and he would assist me, he is not that far for me to reach him with my phone - he is my neighbor I could have a problem even at night and be able to reach out to him." -Male pharmacy purchaser: ECP
Seen as part of the community	"I chose it because it has been there for many years even before I was born till the time I finished school. The attendants are just normal. Many people get help from there so I saw it good to also go there." – Female pharmacy purchaser: ECP and injection<br><br>"What I had said about the hospital, when you get there you will find the person who served you before is transferred but when you come to the chemist you will find the person that served you before." – Female community member (FGD)
Non-judgmental	"I thought at the chemist they will understand me and I would talk to them [better] than at the hospital where they will say I do not need to use those things or even talk to me harshly." –Male pharmacy purchaser: ECP and condoms

For peer review only - http://bmjopen.bmj.com/site/about/guidelines.xhtml

	"At the chemist, that person wants - since it is a business – [to] just give, as compared to the hospital where when you get there you will find nurses who are arrogant or other doctors who will insult you." Male community member (FGD)
**Service appeal**	*The contraception-purchasing transaction*
Speed	You know at the dispensary it is a must you meet with the doctor for more explanation. And maybe there is a service you need to pay for, the expenses are many at the dispensary unlike the chemist where everything is fast, when you get there you get what you want and leave. – Youth female, has purchased ECP and condoms<br><br>"You get in a hospital, there are so many people queueing outside that are waiting to see a doctor. Here comes a young lady who is in a hurry. That particular person will find it more convenient to go to a chemist shop rather than going to a hospital." – Pharmacist
Cost	It is not easy for the government hospital. It is best, if you have money, you go to private hospitals. Now that is why you see if someone does not have money, or us the young people, we just go to the chemist because there is no cash to see a doctor for Ksh 600. At the chemist you just go direct and you are served. – Male pharmacy purchaser: ECP and condoms<br><br>Chemists are not expensive like hospitals. In hospital you can be told it is a government hospital but you end up being asked to give out a lot of money. In [the] chemist the money you get asked is for[paying for] P2 [an emergency contraceptive], yah but in hospital you will be told to do some test because we think it is this and this.– Female pharmacy purchaser: ECP<br><br>Free does not always mean free. Sometimes, something will be free, but by the time you get it, the process is a lot. Because for us, we don't just offer family planning, we do [mandatory] counselling. The person who is going to a chemist is someone who has made up his or her mind. But in the public facilities, you are counselled, you are explained to, you are told the different methods, then you are given a chance to make an informed choice. So, I think that…is a barrier somehow. – Ministry of Health Official, County level

Pharmacy outlets were appealing because of the convenience and anonymity they offered young clients. Pharmacies were located where young people lived, worked, and spent time, making them easy contraception access points. If one pharmacy lacked what a young person was looking for, it was a short trip to the next one. 'Convenience' also extended to the days and hours pharmacies were open. This made them especially important on days where health facilities were known to be busy, or evening and weekend hours when young people might need contraception.

Additionally, the relative privacy offered by pharmacies was especially important to young clients. Participants perceived pharmacies, with interactions limited to a pharmacy attendant and a client, to be far more discreet than similar services offered at public health facilities. Public health facilities had public waiting areas where young people may see someone they knew. Additionally, services in the health facility might be categorized by service type (for example, contraceptive services separated from immunization services, etc). This left young clients feeling particularly exposed should they need to walk up to a labeled 'family planning' window or step forward if a public announcement about contraceptive services was made.

For peer review only - http://bmjopen.bmj.com/site/about/guidelines.xhtml

The individuals behind the counter, and how they interacted with young people, were additional reasons young people preferred to obtain contraception from pharmacies. Pharmacy personnel were perceived to be established, fellow community members. Young clients appreciated seeing the same familiar faces, with less of the personnel turnover associated with public health facilities. When personnel were a similar age to young clients (a very strong preference of all young participants), many reported being able to communicate openly with pharmacy personnel and being more comfortable interacting with them.

Pharmacy personnel were perceived to be non-judgmental compared with those working in health facilities. There was a perception that a trip to a facility would result in difficult questions, and a possible refusal to provide the desired contraceptive. Pharmacy personnel, by contrast, would treat young people "well". That is, they would provide the desired contraceptive without interrogation. Several participants speculated that the for-profit aspect of pharmacies could be a reason that they were treated better and not refused services.

Finally, pharmacy contraception services themselves were appreciated for being fast and cheap. Participants routinely referenced the queueing for services and long wait times driving young people away from health facilities and into pharmacies instead. Services were also perceived to be cheaper than both private health facility services as well as public health facility services. Private health facilities were considered out of financial reach for most young people – making a pharmacy a more affordable option. However, at public health facilities, where contraception-related services are meant to be free, participants indicated that this was often not the case in practice. Expenses related to travel, or 'tests' (for example, a pregnancy test) ordered by health care providers prior to dispensing contraception made real costs related to public services difficult to predict. Finally, as one government official acknowledged, even when services were free, the time and processes required could deter young people who knew what they wanted from going to facilities.

DISCUSSION

This mixed-method study determined pharmacies to be the most popular source of contraception for young people in a peri-urban area of Kwale County. In total, 59% of participants (and 63% female participants) who had ever had sex and self-reported use of a modern contraceptive at last sexual intercourse had obtained their contraception from a pharmacy. This is higher than previously reported for Kenya as a whole. [8] Multivariable analyses indicated that young people who were still living at home with family relied more heavily on pharmacies for contraception more than their peers. That said, the strongest predictor of young people's contraception coming from pharmacies was the type of contraception they used, specifically emergency contraception. Qualitative findings demonstrated that young people valued pharmacies for their convenience, anonymity, non-judgmental and personable staff, service speed, as well as predictable and affordable prices.

Together, these mixed methods indicate that pharmacies provide a valued source of contraception for those young people who may face increased scrutiny or gatekeeping in health facilities. For young people using condoms or ECP, the reported convenience and speed of service explains the strong preference for pharmacies. Following unprotected sex, a young person needing ECP would understandably prefer to pay for it at a nearby pharmacy instead of traveling to a health care facility, waiting in line, and negotiating with a possibly reluctant health worker to obtain it for free (assuming the public facility stocked ECP [20]).

This study had several limitations. In the survey, participants were asked to specify where they or their partner had obtained the contraception used at last sexual intercourse. This question is standard in

For peer review only - http://bmjopen.bmj.com/site/about/guidelines.xhtml

studies looking to establish contraception prevalence. However, our not further ascertaining whether it was the respondent or their partner *who picked up the contraception* affected our ability to distinguish differences in preferred sources between young men who obtain contraception versus young women who obtain contraception. Second, to recruit young people who had recently purchased contraception from pharmacies, we relied on assistance from five pharmacies, purposively selected. It is possible that young purchasers patronizing different pharmacies might have had different experiences than those captured here. Finally, our youth participants in focus group discussions may have felt uncomfortable discussing contraceptive use in a group; we attempted to mediate this by structuring discussion around vignettes of 'typical' young people. This study is strengthened by its mixed methods design and its use of multiple qualitative methods, and inclusion of both pharmacy personnel and young people to triangulate research findings on a sensitive subject.

Our quantitative findings differ substantially from an analysis of Kenya's DHS (KDHS) data, which found that nationwide, 13% of Kenyan women aged 15-24 currently using contraception reported obtaining it at a commercial drug seller. [8] There may be several reasons for this, in addition to the four years between the KDHS and our own data collection. Our study area was a peri-urban setting while the DHS analysis uses nationwide data. Over 70% of Kenya's population is rural. [21] Finally, our study's inclusion of emergency contraception and measuring contraception use at last sexual intercourse (rather than 'current use') is also a likely contributor. Twelve percent of participants in this study used emergency contraception at last sexual intercourse, and the KDHS did not specifically capture emergency contraception use [22]. The DHS's measures of contraception 'current use' in general has been previously critiqued for not being able to capture contraceptive methods which may be used periodically, including ECP.[23] Our link between ECP purchasers and pharmacies are in line with earlier data from urban Kenya, which indicated that upwards of 96% of adult women needing emergency contraception obtained it at a pharmacy.[24]

By contrast, our qualitative findings were largely in line with previous research. One systematic review featuring studies mostly from high-income countries (HICs) affirms that young people appreciate pharmacies for their convenience, speed of service and ease of contraception access.[9] However, this review also reported mixed evidence (all from HICs) as to whether pharmacy services were considered 'private'[9], while our study found an almost universal appreciation of pharmacies for their anonymity/privacy. This difference may be a result of different dispensing protocols and establishment layouts in pharmacies and public health facilities in HICs vs LMICs. Preliminary evidence from other LMICs corroborates our findings that among young people[25], and the general population[26], pharmacies' contraception services are appreciated for the privacy offered.

While this study focused on pharmacies, its findings also cover perceptions around how contraception services are delivered to young people in public health facilities. Pharmacies were naturally contrasted with health facilities when participants explained young people's preferences and were perceived to be everything that health facilities were not: fast, private and non-limiting. The extra 'procedures' required to obtain contraception in health facilities – which in many cases are unnecessary [27] and have been demonstrated in other settings to limit access[28, 29] - were especially unwelcome for young people, who were uninterested in extended counselling and wary of laboratory tests. As a result, pharmacy services were deemed more 'predictable' than those obtained in health facilities (public or private).

For Kenya, pharmacies are likely to remain a preferred choice of contraception as long as barrier methods and short-acting forms of contraception are popular with young people[22]. Policymakers should therefore recognize their role as contraception providers, especially for a community's younger members. Finding ways to link the myriad licensed pharmacies to focal points in public health facilities

For peer review only - http://bmjopen.bmj.com/site/about/guidelines.xhtml

could strengthen a supportive 'network' of accessible and appealing contraception services available to young people. A similar hub-and-spoke approach is used in the implementation of Kenya's broader Community Health Strategy, where community health volunteers are embedded within the community and report back to a facility-based community health extension worker.[30] Such a system, complemented by improved adolescent-friendliness of public health facilities, would also enable easier referral of young people to providers who can offer them more effective forms of contraception. However none of this can succeed without taking needed steps to improve pharmacy regulation, personnel training, and the overall quality of services.[31]

Our data revealed that shops were the second most popular source of contraception for young men. The reliance on shops and lower-level drug dispensaries is seen elsewhere in the region: one survey in Nigeria found that among young people age 15 to 24, around half sourced their contraception from 'chemists/patent medicine shops' (a cadre of establishment below pharmacies, which does not exist in Kenya).[32] Unfortunately, exploring shops in further detail was beyond the scope of our data collection. Additional research is needed to understand how to incorporate these more informal sources into contraception interventions. That said, integrating them into the broader 'network' of contraception providers for young people will be even more challenging: lower-level drug dispensers are only peripherally associated with the health system in many settings, while shops are not associated at all.

Finally, we must acknowledge those still left behind. Of participants who reported ever having sex, almost half of them (49%) had *not* used any modern contraception at last sexual intercourse. These are young people who are not being reached by the current network of public and private health facilities, pharmacies, and even neighborhood shops. They are a reminder that improving the quality of services in these outlets is necessary but not sufficient to address young people's contraceptive needs. There is a continued need for multi-sectoral interventions, including comprehensive sexuality education, to increase demand for contraception among youth (dispelling myths, addressing taboos and stigma, and increasing agency) [33], address barriers to accessing it (including community norms around acceptability) [3], and promote uptake of highly effective forms of contraception.

Young people in Coastal Kenya steadily rely on pharmacies for contraception and often prefer them to health facility services. Many of the pharmacy qualities most appreciated by young participants are also hallmarks of youth-friendly health services, which should be available in any outlet a young person accesses health services. [19, 34] If a young person chooses to use modern contraception, their selection of an outlet will be determined by several factors, including the type of contraception desired, living situation, and relationship status. Collaboration between health facilities and retail pharmacies at local levels can exchange operational strengths between these providers. Then, wherever a young person presents for contraceptive services, they encounter one part of a supportive network of quality providers.

ACKNOWLEDGEMENTS

We appreciate the support of Jefferson Mwaisaka and Winnie Wangari. Our sincere thanks to all data collectors and participants. The manuscript represents the view of the named authors only.

For peer review only - http://bmjopen.bmj.com/site/about/guidelines.xhtml

FUNDING STATEMENT

This work was partially supported by the UNDP/UNFPA/UNICEF/WHO/World Bank Special Programme of Research, Development and Research Training in Human Reproduction (HRP).

COMPETING INTERESTS

None declared

DATA AVAILABILITY STATEMENT

The full deidentified quantitative dataset can be made available on request to corresponding author. Qualitative data cannot be shared publicly, as consent procedures for participants did not include making full interview and focus group discussion transcripts publicly available. However, transcript excerpts are available to researchers on request from the corresponding author and following approval from the University of Nairobi/Kenyatta National Hospital Ethics Committee (contact via uonknh_erc@uonbi.ac.ke).

AUTHOR CONTRIBUTORSHIP

LG conceived of the study and developed the protocol with substantive input from KW and AMH. PG, was Principal Investigator of the AMADILLO study and thereby supported LG in setting up this study's infrastructure in Kenya. LG trained and supervised data collectors, with guidance from PG. JAC and MW developed the statistical analysis plan. LG led the manuscript writing with substantive input from KW and AMH. All authors reviewed and edited drafts.

REFERENCES

1. MacQuarrie KLD. Unmet need for family planning among young women: Levels and trends. Rockville, MD: ICF International; 2014.
2. Chandra-Mouli V, McCarraher DR, Phillips SJ, Williamson NE, Hainsworth G. Contraception for adolescents in low and middle income countries: needs, barriers, and access. Reproductive Health. 2014;11(1):1.
3. High-Impact Practices in Family Planning (HIPs). Adolescent-friendly contraceptive services: mainstreaming adolescent-friendly elements into existing contraceptive services. . USAID; 2015.
4. (JSI) JSI. Private Shops and Pharmacies as Providers of Family Planning. 2016.
5. Corroon M, Kebede E, Spektor G, Speizer I. Key Role of Drug Shops and Pharmacies for Family Planning in Urban Nigeria and Kenya. Global Health: Science and Practice. 2016;4(4):594-609.
6. High-Impact Practices in Family Planning (HIP). Drug Shops and Pharmacies: Sources for family planning commodities and information. Washington DC: USAID; 2013.
7. Ostola JC, M., Speizer I, Kebede E, Spektor G. Private sector distribution of family planning: The role of drug shops and pharmacies in urban Kenya and Nigeria. 2015.

For peer review only

For peer review only - http://bmjopen.bmj.com/site/about/guidelines.xhtml

8. Radovich E, Dennis ML, Wong KLM, Ali M, Lynch CA, Cleland J, et al. Who Meets the Contraceptive Needs of Young Women in Sub-Saharan Africa? Journal of Adolescent Health. 2018;62(3):273-80.
9. Gonsalves L, Hindin MJ. Pharmacy provision of sexual and reproductive health commodities to young people: a systematic literature review and synthesis of the evidence. Contraception. 2017;95(4):339-63.
10. World Health Organization. Family planning/Contraception Geneva, Switzerland: World Health Organization; 2018 [Available from: https://www.who.int/news-room/fact-sheets/detail/family-planning-contraception.
11. Ministry of Health/Kenya. National family planning guidelines for service providers: Updated to reflect the 2009 Medical Eligibility Criteria of the World Health Organization. Nairobi, Kenya: Division of Reproductive Health; 2010.
12. National Council for Law Reporting with the Authority of the Attorney-General/Kenya. Pharmacy and Poisons Act. Chapter 244. 1957 (revised 2012)(Cap 244).
13. Kwale County Government. Kwale County Integrated Development Plan (2018-2022): Continuing Kwale's Transformation Together 2018.
14. Kenya Ministry of Health. Kwale county: Health at a glance. 2015.
15. Gonsalves L, Hindin MJ, Bayer A, Carcamo CP, Gichangi P, Habib N, et al. Protocol of an open, three-arm, individually randomized trial assessing the effect of delivering sexual and reproductive health information to young people (aged 13–24) in Kenya and Peru via mobile phones: adolescent/youth reproductive mobile access and delivery initiative for love and life outcomes (ARMADILLO) study stage 2. Reproductive Health. 2018;15(1):126.
16. Gonsalves L, Njeri WW, Schroeder M, Mwaisaka J, Gichangi P. Research and Implementation Lessons Learned From a Youth-Targeted Digital Health Randomized Controlled Trial (the ARMADILLO Study). JMIR Mhealth Uhealth. 2019;7(8):e13005.
17. Charmaz K. Constructing grounded theory: A practical guide through qualitative analysis. London, UK: SAGE Publications; 2006.
18. Gale NK, Heath G, Cameron E, Rashid S, Redwood S. Using the framework method for the analysis of qualitative data in multi-disciplinary health research. BMC Medical Research Methodology. 2013;13(1):117.
19. World Health Organization. Making health services adolescent friendly: developing national quality standards for adolescent friendly health services. Geneva, Switzerland: World Health Organization; 2012.
20. Riley C, Garfinkel D, Thanel K, Esch K, Workalemahu E, Anyanti J, et al. Getting to FP2020: Harnessing the private sector to increase modern contraceptive access and choice in Ethiopia, Nigeria, and DRC. PLOS ONE. 2018;13(2):e0192522.
21. The World Bank. Rural population (% of total population): World Bank staff estimates based on the United Nations Population Division's World Urbanization Prospects: 2018 Revision. New York, USA: World Bank Group; 2018 [Available from: https://data.worldbank.org/indicator/SP.RUR.TOTL.ZS.
22. Kenya National Bureau of Statistics, Ministry of Health/Kenya, National AIDS Control Council/Kenya, Kenya Medical Research Institute, Population NCf, Development/Kenya. Kenya Demographic and Health Survey 2014. Rockville, MD, USA; 2015.
23. Marston C, Renedo A, Nyaaba GN, Machiyama K, Tapsoba P, Cleland J. Improving the Measurement of Fertility Regulation Practices: Findings from Qualitative Research in Ghana. Int Perspect Sexual Reprodud Health. 2017;43(3):111-9.
24. International Consortium for Emergency Contraception. Counting what counts: Tracking access to emergency contraception - Kenya. 2013.

For peer review only - http://bmjopen.bmj.com/site/about/guidelines.xhtml

25. Cartwright A, Otai J, Maytan-Joneydi A, McGuire C, Sullivan E, Olumide A, et al. Access to family planning for youth: perspectives of young family planning leaders from 40 countries [version 1; peer review: 1 approved with reservations]. Gates Open Research. 2019;3(1513).
26. Peterson J, Brunie A, Diop I, Diop S, Stanback J, Chin-Quee D. Over the counter: The potential for easing pharmacy provision of family planning in urban Senegal [version 3; peer review: 1 approved, 2 approved with reservations]. Gates Open Research. 2019;2(29).
27. World Health Organization. Selected practice recommendations for contraceptive use: Third edition. Geneva, Switzerland; 2016.
28. Grossman D, Ellertson C, Abuabara K, Blanchard K, Rivas FT. Barriers to contraceptive use in product labeling and practice guidelines. Am J Public Health. 2006;96(5):791-9.
29. Leeman L. Medical Barriers to Effective Contraception. Obstetrics and Gynecology Clinics of North America. 2007;34(1):19-29.
30. Oliver M, Geniets A, Winters N, Rega I, Mbae SM. What do community health workers have to say about their work, and how can this inform improved programme design? A case study with CHWs within Kenya. Global Health Action. 2015;8(1):27168.
31. Gonsalves L, Wyss K, Gichangi P, Say L, Martin Hilber A. Regulating pharmacists as contraception providers: A qualitative study from Coastal Kenya on injectable contraception provision to youth. PLOS ONE. 2019;14(12):e0226133.
32. Oye-Adeniran BA, Adewole IF, Umoh AV, Oladokun A, Gbadegesin A, Odeyemi KA, et al. Sources of Contraceptive Commodities for Users in Nigeria. PLOS Medicine. 2005;2(11):e306.
33. World Health Organization. Family planning evidence brief: Reducing early and unintended pregnancies among adolescents. Geneva, Switzerland: World Health Organization; 2017.
34. Erulkar AO, CJ; Phiri, A;. What is youth-friendly? Adolescents' preferences for reproductive health services in Kenya and Zimbabwe. African Journal of Reproductive Health. 2005;9(3):51-8.

For peer review only - http://bmjopen.bmj.com/site/about/guidelines.xhtml

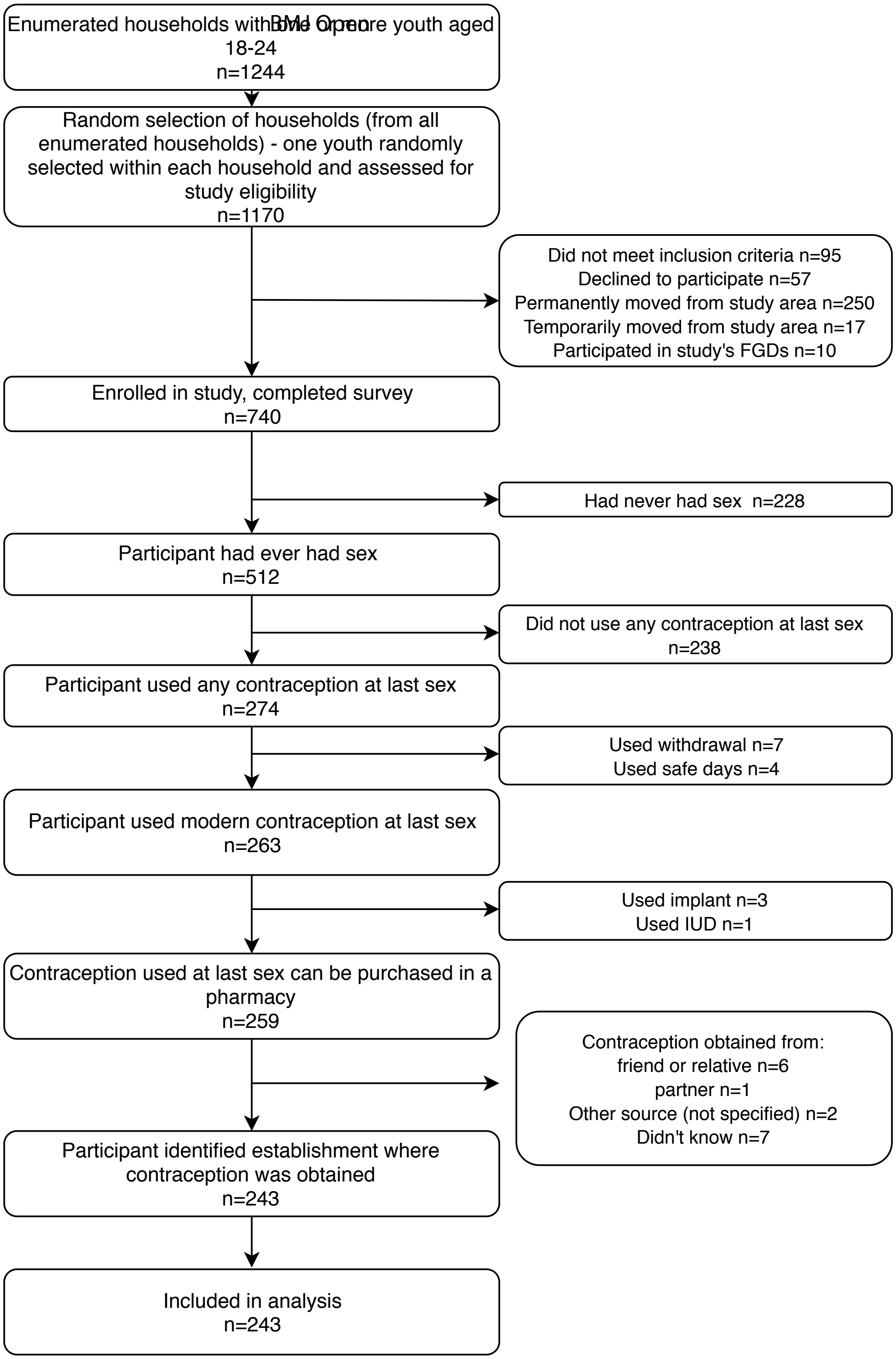
Enumerated households with one or more youth aged 18-24
n=1244
Random selection of households (from all enumerated households) - one youth randomly selected within each household and assessed for study eligibility
n=1170
Did not meet inclusion criteria n=95
Declined to participate n=57
Permanently moved from study area n=250
Temporarily moved from study area n=17
Participated in study's FGDs n=10
Enrolled in study, completed survey
n=740
Had never had sex n=228
Participant had ever had sex
n=512
Did not use any contraception at last sex
n=238
Participant used any contraception at last sex
n=274
Used withdrawal n=7
Used safe days n=4
Participant used modern contraception at last sex
n=263
Used implant n=3
Used IUD n=1
Contraception used at last sex can be purchased in a pharmacy
n=259
Contraception obtained from:
friend or relative n=6
partner n=1
Other source (not specified) n=2
Didn't know n=7
Participant identified establishment where contraception was obtained
n=243
Included in analysis
n=243

Supplementary Table 1 Selected characteristics of young people purchasing contraception at a pharmacy, shop, or any other source

	Pharmacy (N=153)	Shop (N=29)	Any other source (N=61)
Age			
18-19	27 (18%)	6 (21%)	10 (16%)
20+	126 (82%)	23 (79%)	51 (84%)
Sex			
Male	80 (52%)	24 (83%)	28 (46%)
Female	73 (48%)	5 (17%)	33 (54%)
Education			
Primary or below	60 (39%)	7 (24%)	29 (48%)
Secondary or above	93 (61%)	22 (76%)	32 (52%)
Relationship status			
Single	46 (30%)	10 (34%)	25 (41%)
Dating	86 (56%)	18 (62%)	11 (18%)
Cohabiting/Married	21 (14%)	1 (3%)	25 (41%)
Children			
No	139 (91%)	28 (97%)	37 (61%)
Yes	14 (9%)	1 (3%)	24 (39%)
Living situation			
Lives alone	30 (20%)	3 (10%)	6 (10%)
Lives with family (dependent)	113 (74%)	25 (86%)	32 (53%)
Lives with child or partner	10 (7%)	1 (3%)	23 (38%)
Contraception purchased			
Condom	120 (78%)	28 (97%)	33 (54%)
ECP	24 (16%)	1 (3%)	5 (8%)
Pills/Injections	9 (6%)	0 (0%)	23 (38%)

For peer review only - http://bmjopen.bmj.com/site/about/guidelines.xhtml

S1. Focus Group Discussion Guide

Today we're going to discuss what young people in this community think about contraceptives and where they go when they need it.

Warm-up

- Tell me what "contraceptive" means to you?
  - Tell me the kinds of contraceptives you've heard of

Myths and misinformation around contraception

Vignette: XXX [name determined by FGD participants] is 21 and her boyfriend YYY [name determined by FGD participants] is 23. They have been dating for awhile and are thinking about using contraceptives. However, there are things about contraceptives that they have heard from friends and family members which make them uncertain.

What are some of the things which they may have heard?

Ask participants to write down on sticky notes at least three things that XX and YY may have heard which would make them nervous. NoteTaker and Facilitator 3 will post these on the board, grouping together the similar ones. After they are all posted, moderator can ask:

- *[read out the reasons listed on the board]:* Are there any additional reasons XX and YY may feel uncertain that you can think of?
- *[also probe on certain reasons that are vague or broad]*

Where young people get contraceptives

- Tell me about all the places in _____ (study site town), where a young person can get contraceptives? *(Facilitator 3 writes out a list)*
- Describe all the different kinds of young people you could find in your community. *(keep this short)*

For each listed contraception source:

- Describe the kind of young person who would go to a _______ if he/she needed contraceptives? *(Draw stick figure under each source name, probe on and label with identifiers: gender, marital status, etc)*
  Facilitator 3 stops drawing after question above
- When would a young person choose to go to a _____ to get contraceptives?
  - [Note]: what kind of contraceptives are they getting
- Why would this young person choose to go to a ________ to get contraceptives over another source?

For peer review only

- [Probe] What are the best qualities about _______ as a resource for contraceptives?
- What might other young people *dislike* about _________ as a resource for contraceptives?

Qualities of ideal FP-dispensing in *non-service sources*

- What are the most important qualities a chemist or a shop needs to have for a young person to be comfortable obtaining contraceptives? [*Probe on person working vs the shop itself]*
- What could be some reasons why young people would not be comfortable going to chemists or shops?
  - What could be done to increase the comfort of young people who might not be comfortable going to chemists or shops?
- What **other** information and services would a young person needing contraceptives from a chemist or a shop also need?
  - [*be sure to probe on information AND services separately]*
- What could be done to make sure that young people can get the extra information and services (*that group mentioned in previous question*) that they need from chemists and shops without being uncomfortable and without sacrificing their privacy and speed (*or whatever is mentioned as an important quality).*

Close and thank people for their time

For peer review only

S2. In-Depth Interview guide for young contraception purchasers

Warm-up

- Tell me about what life is like for young people (people your age) in this community.
- What are some of the challenges that young people face?

As you told us earlier, you recently purchased family planning from a chemist shop nearby. I want to ask you about this experience

- Tell me about what your experience was like when purchasing FP from the chemist – how did it go, from beginning to end? *[looking for information on environment, interaction with chemists, how they were treated]*
    - How did you feel at each step?
    - What was the most difficult part of the experience?
    - What was the easiest part of the experience?
- Describe your interaction with the chemist attendant *[probe on: how were you treated? Did they give you advice]*
    - How did he/she react to your request
- Tell me about the information you were given by the chemist *[probe on: counselling, life advice, side effects, referrals other FP]*
- Tell me about what else was going on in the chemist shop while you were purchasing FP.
- How did you feel after you left the shop?
- Given the experience you've just described to me, how did that compare with what you *thought* would happen when you first walked in the chemist shop? *

Thinking about the time that you purchased family planning at the chemist, help me understand how you made that decision:

- What situation made you decide that you needed family planning? *[Probe on whether others were involved in this decision]*
- How did you decide what kind of family planning you wanted?
- Why did you decide to go to a chemist for family planning instead of other places?
    - Why did you select that particular chemist?
- How did this experience compare with other times you have bought family planning?

As a young person who has purchased FP from a chemist, I am interested to hear your ideas for how chemist shops could be improved for young people:

- Were there any parts of your experience that you liked?
- Based on your experience, was there anything you would've liked to happen that didn't?
    - *Probe (if necessary):* Based on these, is there any part of the experience that you want changed?
- If you worked at the chemist, what would you do to make young clients buying family planning feel comfortable?
- If you worked at the chemist, how could you improve the shop to be more friendly to young people needing family planning?

For peer review only

- What else do young people need to feel comfortable getting FP from chemists?

For peer review only

S3. Key Informant Interview guide (for a person working in a pharmacy)

Group 1 (Background – Personal)

- Tell me about yourself and how you came to work in the chemist?
  - Probe if they are from the area
  - What is their current title?
- Tell me about the roles and responsibilities of your job - describe a typical day of work
- What are the things that you enjoy about your job?
- What are the things you do not enjoy about your job?

Group 2 (Background – Shop)

- Tell me about who else works at this chemist
  - Probe: what are their roles and how are they different from yours?
- Describe how the chemist shop is organized?
- When are you busiest?
  - Probe: opening hours

Group 3 (Family planning)

- Tell me about the family planning in this chemist shop
  - Probe: what kinds are available, most popular, price
- Tell me about the kinds of people from the community who buy these family planning
  - Probe: Describe them, what they are looking for
- Why are chemist shops like yours important in providing family planning to the community?
  - Probe: How is this job different from health facilities that also have family planning?
- If a young person comes in asking for family planning, what are some of things you look at that help you decide what to recommend?
- What are the rules for dispensing family planning?
  - Probe: are there any exceptions to these rules?
- Describe the kinds training (either from your boss or from previous training) you received about family planning?

Group 4 (Feelings about selling family planning to young people)

- Think about the last time that young person (18-24) came to this chemist for some kind of family planning. Can you describe the interaction, from beginning to end?
  - Probe: what happens, what would they say, what would you say, what do you give them?
- How do young customers feel coming to ask for family planning (Probe: what do they say)
- What could chemist shops like this one do to improve the comfort of young people in the community who need family planning?
- When a young customer comes in asking for family planning, how do you feel?

For peer review only

  - Are things you would like to tell them?
- If you had the power, what would you do to improve the confidence of chemists to provide family planning to young people?

For peer review only

S4. Tool 2 – Survey Instrument (Excerpt)

SOCIO-DEMOGRAPHIC INFORMATION. First we're going to talk about who you are.

1. What is your sex? Mark ONLY ONE.

   0 Male
   1 Female

2. What is your birthdate?

   Day |__|__| Month |__|__| Year |__|__|__|__|

3. What is the highest level of school you attended? Mark ONLY ONE.

   0 I've never gone to school
   1 Primary school
   2 Secondary school
   3 Post-secondary education **– GO TO 5**

4. What is the highest grade you completed at that level?

   |__|__| grade/form/level **– GO TO 6**

5. What type of post-secondary education did you attend/are you attending? Mark ONLY ONE.

   1 Technical post-secondary education
   2 University education

6. Who do you currently live with? Mark ALL possible options.

   0 I live alone
   1 Father/stepfather
   2 Mother/stepmother
   3 Siblings
   4 Grandparents
   5 Other relatives

For peer review only

6 Husband or wife ***– NOTE: Be sure to ask whether husband/wife or cohabiting partner.***
7 Cohabiting partner
8 In-laws
9 Children
10 Friends

7. What is your current relationship status?
   0 Single
   1 Friends with benefits
   2 Dating
   3 Cohabiting
   4 Engaged
   5 Married
   6 Other (specify)

8. How many children do you have?

   0 I have no children **– GO TO 10**

   |__|__| child/children

9. How old were you when you had your first child?

   |__|__| years old

…
[SURVEY CONTINUES]

ARMADILLO-RELATED BEHAVIORS. Now we're going to talk about sexual activity in order to gain a better understanding of some important life issues. Let me assure you again that your answers are completely confidential and will not be told to anyone.

29. How old were you when you had sexual intercourse for the very first time?

   |__|__| years old

   0 I have never had sexual intercourse **– GO TO 46**

30. Have you ever used any method to prevent pregnancy? By use, I mean that either you, yourself, have used the method or that a partner of yours used the method when having sex with you.

For peer review only

YES 1
NO...0
DON'T KNOW...8
REFUSED 9

31. When was the last time you had sex?

|__|__| days ago

|__|__| weeks ago

|__|__| months ago

|__|__| years ago

32. The last time you had sex, what was your relationship to this person with whom you had sexual intercourse?

1 Boyfriend not living with respondent
2 Girlfriend not living with respondent
3 Male cohabiting partner
4 Female cohabiting partner
5 Husband
6 Wife
7 Male casual acquaintance
8 Female casual acquaintance
9 Male sex worker
10 Female sex worker
11 Female client (respondent is male sex worker)
12 Male client (respondent is female sex worker)
13 Male relative
14 Female relative

33. The last time you had sex, did you or your partner use a contraceptive method?

0 No **– GO TO 45**
1 Yes

34. The last time you had sex, which contraceptive method did you or your partner use?

For peer review only

1 Male condom
2 Female condom
3 Birth control pill
4 Injectable
5 Implant
6 Intrauterine device (IUD)
7 Emergency contraception (the morning after pill)
8 Female sterilization
9 Male sterilization (vasectomy)
a. Withdrawal **– GO TO 44**
10 Rhythm method **– GO TO 44**

35. The last time you had sex, where did you or your partner obtain the contraceptive method you used?

1 A pharmacy or chemist
2 County Hospital
3 Health centres
4 A NGO
5 A private doctor or clinic
6 A shop/market
7 A community-based distributor
8 A peer educator
9 A traditional healer **– GO TO 44**
10 A friend or relative **– GO TO 44**
11 A partner **– GO TO 44**
12 Other **– GO TO 44**

36. When you obtained your [MOST RECENT CONTRACEPTIVE METHOD], were you told by the provider about side effects of problems you might have with a method to delay or avoid getting pregnant?
1 Yes
2 No

37. Were you told what to do if you experienced side effects or problems?
1 Yes
2 No

38. At that time, were you told by the family planning provider about methods of family planning other than [MOST RECENT CONTRACEPTIVE METHOD] that you could use?
1 Yes
2 No

39. During that visit did you obtain the method you wanted to delay or avoid getting pregnant?
1 Yes **– GO TO 42**

For peer review only

2 No

40. Why didn't you obtain the method you wanted?
  1 Method out of stock that day
  2 Method not available at all
  3 Provider not trained to provide the method
  4 Provider recommended a different method
  5 Not eligible for method
  6 Decided not to adopt a method
  7 Too costly
  8 Other

41. During that visit who made the final decision about what method you got?
  1 You alone
  2 Provider
  3 Partner
  4 You and provider
  5 You and partner
  6 Other

42. Would you return to this provider?
  1 Yes
  2 No

43. Would you refer your relative or friend to this provider/facility?
  1 Yes
  2 No

44. How important were each of the following characteristics to you in deciding which birth control method to use? *(read item, asking)* Would you say: not at all important, slightly important, quite important or extremely important to you in choosing a method?

	Not at all important	Slightly important	Quite important	Extremely important
A. It is very effective at preventing pregnancy				
B. It has a low cost.				

For peer review only

C. It is easy to use.				
D. It doesn't contain hormones.				
E. It is acceptable to my partner<br><br>F. It doesn't interrupt sex.				
G. It is effective at preventing HIV or STIs.				

For peer review only

Standards for Reporting Qualitative Research (SRQR)*

http://www.equator-network.org/reporting-guidelines/srqr/

Page/line no(s).

Title and abstract

**Title** - Concise description of the nature and topic of the study Identifying the study as qualitative or indicating the approach (e.g., ethnography, grounded theory) or data collection methods (e.g., interview, focus group) is recommended	Page 1/Line 1&2 (identified as mixed methods)
**Abstract** - Summary of key elements of the study using the abstract format of the intended publication; typically includes background, purpose, methods, results, and conclusions	Page 1-2

Introduction

**Problem formulation** - Description and significance of the problem/phenomenon studied; review of relevant theory and empirical work; problem statement	Page 3/Line 67-73, 87-89
**Purpose or research question** - Purpose of the study and specific objectives or questions	Page 3/Line 89-91

Methods

**Qualitative approach and research paradigm** - Qualitative approach (e.g., ethnography, grounded theory, case study, phenomenology, narrative research) and guiding theory if appropriate; identifying the research paradigm (e.g., postpositivist, constructivist/ interpretivist) is also recommended; rationale**	(see response to reviewers Page 3)
**Researcher characteristics and reflexivity** - Researchers' characteristics that may influence the research, including personal attributes, qualifications/experience, relationship with participants, assumptions, and/or presuppositions; potential or actual interaction between researchers' characteristics and the research questions, approach, methods, results, and/or transferability	Page 5/Line 166-175
**Context** - Setting/site and salient contextual factors; rationale**	Page 3/Line 93-98
**Sampling strategy** - How and why research participants, documents, or events were selected; criteria for deciding when no further sampling was necessary (e.g., sampling saturation); rationale**	Page 4/113-147
**Ethical issues pertaining to human subjects** - Documentation of approval by an appropriate ethics review board and participant consent, or explanation for lack thereof; other confidentiality and data security issues	Page 5/Line151-155
**Data collection methods** - Types of data collected; details of data collection procedures including (as appropriate) start and stop dates of data collection and analysis, iterative process, triangulation of sources/methods, and modification of procedures in response to evolving study findings; rationale**	Page5/Line141-151

For peer review only

**Data collection instruments and technologies** - Description of instruments (e.g., interview guides, questionnaires) and devices (e.g., audio recorders) used for data collection; if/how the instrument(s) changed over the course of the study	Page 5/Line 141-151 (and Supplementary materials)
**Units of study** - Number and relevant characteristics of participants, documents, or events included in the study; level of participation (could be reported in results)	Page 4/Table 1 Page 9/Line 243-254
**Data processing** - Methods for processing data prior to and during analysis, including transcription, data entry, data management and security, verification of data integrity, data coding, and anonymization/de-identification of excerpts	Page 6/Line192-194
**Data analysis** - Process by which inferences, themes, etc., were identified and developed, including the researchers involved in data analysis; usually references a specific paradigm or approach; rationale**	Page6/Line194-203
**Techniques to enhance trustworthiness** - Techniques to enhance trustworthiness and credibility of data analysis (e.g., member checking, audit trail, triangulation); rationale**	Page 6/Line193-194

Results/findings

**Synthesis and interpretation** - Main findings (e.g., interpretations, inferences, and themes); might include development of a theory or model, or integration with prior research or theory	Page 9/From Line 255 to end of section
**Links to empirical data** - Evidence (e.g., quotes, field notes, text excerpts, photographs) to substantiate analytic findings	Page 9/Table 4

Discussion

**Integration with prior work, implications, transferability, and contribution(s) to the field -** Short summary of main findings; explanation of how findings and conclusions connect to, support, elaborate on, or challenge conclusions of earlier scholarship; discussion of scope of application/generalizability; identification of unique contribution(s) to scholarship in a discipline or field	Page 12/Entire discussion section
**Limitations** - Trustworthiness and limitations of findings	Page12/Line316-328

Other

**Conflicts of interest** - Potential sources of influence or perceived influence on study conduct and conclusions; how these were managed	Page 14/Line 405
**Funding** - Sources of funding and other support; role of funders in data collection, interpretation, and reporting	Page 14/Line402-403

*The authors created the SRQR by searching the literature to identify guidelines, reporting standards, and critical appraisal criteria for qualitative research; reviewing the reference lists of retrieved sources; and contacting experts to gain feedback. The SRQR aims to improve the transparency of all aspects of qualitative research by providing clear standards for reporting qualitative research.

For peer review only

For peer review only - http://bmjopen.bmj.com/site/about/guidelines.xhtml

**The rationale should briefly discuss the justification for choosing that theory, approach, method, or technique rather than other options available, the assumptions and limitations implicit in those choices, and how those choices influence study conclusions and transferability. As appropriate, the rationale for several items might be discussed together.

Reference:
O'Brien BC, Harris IB, Beckman TJ, Reed DA, Cook DA. **Standards for reporting qualitative research: a synthesis of recommendations.** *Academic Medicine*, Vol. 89, No. 9 / Sept 2014 DOI: 10.1097/ACM.0000000000000388

For peer review only

STROBE Statement—checklist of items that should be included in reports of observational studies

	Item No.	Recommendation	Page No.	Relevant text from manuscript
**Title and abstract**	1	(*a*) Indicate the study's design with a commonly used term in the title or the abstract	1	Title
		(*b*) Provide in the abstract an informative and balanced summary of what was done and what was found	1-2	Abstract
**Introduction**				
Background/rationale	2	Explain the scientific background and rationale for the investigation being reported	3	Paragraph 1-4 of Introduction
Objectives	3	State specific objectives, including any prespecified hypotheses	3	"Therefore, this mixed methods study sought to answer two questions…"
**Methods**				
Study design	4	Present key elements of study design early in the paper	3-4	"This analysis was part of a broader, mixed-methods study describing how young people (aged 18-24) in Kwale County obtain contraception from pharmacies." + Table 1
Setting	5	Describe the setting, locations, and relevant dates, including periods of recruitment, exposure, follow-up, and data collection	4-5	Table 1 + Methods text
Participants	6	(*a*) *Cohort study*—Give the eligibility criteria, and the sources and methods of selection of participants. Describe methods of follow-up<br>*Case-control study*—Give the eligibility criteria, and the sources and methods of case ascertainment and control selection. Give the rationale for the choice of cases and controls<br>*Cross-sectional study*—Give the eligibility criteria, and the sources and methods of selection of participants	4 (cross-sectional)	Table 1, "In October 2017, data collectors enumerated all households…"
		(*b*) *Cohort study*—For matched studies, give matching criteria and number of exposed and unexposed<br>*Case-control study*—For matched studies, give matching criteria and the number of controls per case		N/A

For peer review only - http://bmjopen.bmj.com/site/about/guidelines.xhtml

Variables	7	Clearly define all outcomes, exposures, predictors, potential confounders, and effect modifiers. Give diagnostic criteria, if applicable	6	Analysis section
Data sources/ measurement	8*	For each variable of interest, give sources of data and details of methods of assessment (measurement). Describe comparability of assessment methods if there is more than one group	N/A	Not included beyond primary outcome (to leave space to discuss qual methods)
Bias	9	Describe any efforts to address potential sources of bias	5	"Data collectors entered responses save…"
Study size	10	Explain how the study size was arrived at	4	"The sample size was calculated based on the ARMADILLO trial's primary outcome…"
Quantitative variables	11	Explain how quantitative variables were handled in the analyses. If applicable, describe which groupings were chosen and why	6	Analysis section – for primary outcome
Statistical methods	12	(*a*) Describe all statistical methods, including those used to control for confounding	6	Analysis section
		(*b*) Describe any methods used to examine subgroups and interactions	N/A	Based on primary outcome, no subgroups were examined
		(*c*) Explain how missing data were addressed	N/A	Not included (to leave space to discuss qual methods)
		(*d*) *Cohort study*—If applicable, explain how loss to follow-up was addressed<br>*Case-control study*—If applicable, explain how matching of cases and controls was addressed<br>*Cross-sectional study*—If applicable, describe analytical methods taking account of sampling strategy	N/A	N/A
		(*e*) Describe any sensitivity analyses	N/A	N/A
**Results**				
Participants	13*	(a) Report numbers of individuals at each stage of study—eg numbers potentially eligible, examined for eligibility, confirmed eligible, included in the study, completing follow-up, and analysed	N/A	Not included (to leave space to discuss qual methods – reference describing this in detail is included [15] on page 4)
		(b) Give reasons for non-participation at each stage	N/A	Not applicable (cross-section)

For peer review only - http://bmjopen.bmj.com/site/about/guidelines.xhtml

		(c) Consider use of a flow diagram	N/A	Not included (to leave space to discuss qual methods – reference to open source article with this information is included [15] on page 4)
Descriptive data	14*	(a) Give characteristics of study participants (eg demographic, clinical, social) and information on exposures and potential confounders	6-7	Table 2
		(b) Indicate number of participants with missing data for each variable of interest	N/A	None for primary outcome
		(c) *Cohort study*—Summarise follow-up time (eg, average and total amount)		
Outcome data	15*	*Cohort study*—Report numbers of outcome events or summary measures over time		
		Case-control study—Report numbers in each exposure category, or summary measures of exposure		
		Cross-sectional study—Report numbers of outcome events or summary measures	8	Table 3
Main results	16	(*a*) Give unadjusted estimates and, if applicable, confounder-adjusted estimates and their precision (eg, 95% confidence interval). Make clear which confounders were adjusted for and why they were included	8-9	Table 3
		(*b*) Report category boundaries when continuous variables were categorized	8-9	Table 3
		(*c*) If relevant, consider translating estimates of relative risk into absolute risk for a meaningful time period	N/A	N/A
Other analyses	17	Report other analyses done—eg analyses of subgroups and interactions, and sensitivity analyses	N/A	N/A
**Discussion**				
Key results	18	Summarise key results with reference to study objectives	11-12	Discussion paragraph 1
Limitations	19	Discuss limitations of the study, taking into account sources of potential bias or imprecision. Discuss both direction and magnitude of any potential bias	11-12	Discussion paragraph 2
Interpretation	20	Give a cautious overall interpretation of results considering objectives, limitations, multiplicity of analyses, results from similar studies, and other relevant evidence	12	Discussion section paragraph 3-4
Generalisability	21	Discuss the generalisability (external validity) of the study results	12	Discussion section paragraph 3
**Other information**				
Funding	22	Give the source of funding and the role of the funders for the present study and, if applicable, for the original study on which the present article is based	14	Funding statement

For peer review only

For peer review only - http://bmjopen.bmj.com/site/about/guidelines.xhtml

*Give information separately for cases and controls in case-control studies and, if applicable, for exposed and unexposed groups in cohort and cross-sectional studies.

Note: An Explanation and Elaboration article discusses each checklist item and gives methodological background and published examples of transparent reporting. The STROBE checklist is best used in conjunction with this article (freely available on the Web sites of PLoS Medicine at http://www.plosmedicine.org/, Annals of Internal Medicine at http://www.annals.org/, and Epidemiology at http://www.epidem.com/). Information on the STROBE Initiative is available at www.strobe-statement.org.

For peer review only
